# Determinants of the urinary and serum metabolome in children from six European populations

**DOI:** 10.1186/s12916-018-1190-8

**Published:** 2018-11-08

**Authors:** Chung-Ho E. Lau, Alexandros P. Siskos, Léa Maitre, Oliver Robinson, Toby J. Athersuch, Elizabeth J. Want, Jose Urquiza, Maribel Casas, Marina Vafeiadi, Theano Roumeliotaki, Rosemary R. C. McEachan, Rafaq Azad, Line S. Haug, Helle M. Meltzer, Sandra Andrusaityte, Inga Petraviciene, Regina Grazuleviciene, Cathrine Thomsen, John Wright, Remy Slama, Leda Chatzi, Martine Vrijheid, Hector C. Keun, Muireann Coen

**Affiliations:** 1 Division of Computational and Systems Medicine, Department of Surgery and Cancer, Faculty of Medicine, Imperial College London, London, SW7 2AZ UK; 2 Division of Cancer, Department of Surgery and Cancer, Faculty of Medicine, Imperial College London, London, W12 0NN UK; 3 ISGlobal, Barcelona, Spain; 4 Universitat Pompeu Fabra (UPF), Barcelona, Spain; 5 CIBER Epidemiologa y Salud Pública (CIBERESP), Madrid, Spain; 6 MRC-PHE Centre for Environment and Health, School of Public Health, Faculty of Medicine, Imperial College London, London, W2 1PG UK; 7 Department of Social Medicine, Faculty of Medicine, University of Crete, Heraklion, Crete Greece; 8 Inserm, Univ. Grenoble Alpes, CNRS, IAB (Institute of Advanced Biosciences), Grenoble, France; 9 Bradford Institute for Health Research, Bradford Teaching Hospitals NHS Foundation Trust, Bradford, UK; 10 Norwegian Institute of Public Health, Oslo, Norway; 11 Department of Environmental Sciences, Vytautas Magnus University, Kaunas, Lithuania; 12 Department of Preventive Medicine, Keck School of Medicine, University of Southern California, Los Angeles, USA; 13 Oncology Safety, Drug Safety and Metabolism, IMED Biotech Unit, AstraZeneca, 1 Francis Crick Avenue, Cambridge, CB2 0RE UK

**Keywords:** Exposome metabolomics, Metabonomics, Metabolic phenotyping, Epidemiology, Birth cohorts, Paediatrics, NMR spectroscopy, LC-MS, European children, Metabolic profile

## Abstract

**Background:**

Environment and diet in early life can affect development and health throughout the life course. Metabolic phenotyping of urine and serum represents a complementary systems-wide approach to elucidate environment–health interactions. However, large-scale metabolome studies in children combining analyses of these biological fluids are lacking. Here, we sought to characterise the major determinants of the child metabolome and to define metabolite associations with age, sex, BMI and dietary habits in European children, by exploiting a unique biobank established as part of the Human Early-Life Exposome project (http://www.projecthelix.eu).

**Methods:**

Metabolic phenotypes of matched urine and serum samples from 1192 children (aged 6–11) recruited from birth cohorts in six European countries were measured using high-throughput ^1^H nuclear magnetic resonance (NMR) spectroscopy and a targeted LC-MS/MS metabolomic assay (Biocrates Absolute*IDQ* p180 kit).

**Results:**

We identified both urinary and serum creatinine to be positively associated with age. Metabolic associations to BMI z-score included a novel association with urinary 4-deoxyerythreonic acid in addition to valine, serum carnitine, short-chain acylcarnitines (C3, C5), glutamate, BCAAs, lysophosphatidylcholines (lysoPC a C14:0, lysoPC a C16:1, lysoPC a C18:1, lysoPC a C18:2) and sphingolipids (SM C16:0, SM C16:1, SM C18:1). Dietary-metabolite associations included urinary creatine and serum phosphatidylcholines (4) with meat intake, serum phosphatidylcholines (12) with fish, urinary hippurate with vegetables, and urinary proline betaine and hippurate with fruit intake. Population-specific variance (age, sex, BMI, ethnicity, dietary and country of origin) was better captured in the serum than in the urine profile; these factors explained a median of 9.0% variance amongst serum metabolites versus a median of 5.1% amongst urinary metabolites. Metabolic pathway correlations were identified, and concentrations of corresponding metabolites were significantly correlated (*r* > 0.18) between urine and serum.

**Conclusions:**

We have established a pan-European reference metabolome for urine and serum of healthy children and gathered critical resources not previously available for future investigations into the influence of the metabolome on child health. The six European cohort populations studied share common metabolic associations with age, sex, BMI z-score and main dietary habits. Furthermore, we have identified a novel metabolic association between threonine catabolism and BMI of children.

**Electronic supplementary material:**

The online version of this article (10.1186/s12916-018-1190-8) contains supplementary material, which is available to authorized users.

## Background

Under-nutrition during gestation was first proposed in the early 1990s to explain the association observed between low birth weight in infancy and higher mortality rates from cardiovascular disease in male adults [1, 2]. Since then, it has been hypothesised that the origins of many diseases that manifest later in life may be traced back to fetal development—known as the DOHaD (Developmental Origins of Health and Disease) paradigm [3]. In addition, early-life environmental exposures may have wide-ranging consequences for health. Critical windows in development, such as the prenatal period and infancy, have been shown to be particularly susceptible to environmental risk factors that influence disease burden into adulthood [4–6]. For example, prenatal exposure to passive smoke and outdoor air pollutants are acknowledged risk factors for asthma and other allergies including eczema [7, 8], and exposure to endocrine-disrupting and household chemicals have been found to increase obesity risk in children [9, 10]. Moreover, childhood exposure to passive smoke has also been associated with lung cancer risk in adults [11], whilst prenatal infection and exposure to lead have been linked respectively to schizophrenia [12] and attention deficit hyperactivity disorder in children [13]. Growing evidence suggests environmental exposure in early life can also alter molecular phenotypes—such as the epigenome—which then persist throughout life [14, 15]. Consequently, the importance of measuring multiple environmental exposures simultaneously (the exposome) and the impact of this on health at different stages of life are increasingly being recognised [16–20]. Population cohort-based exposome research studies could help address the multi-dimensional interplay between various environmental factors and developmental health outcomes [21]. For example, a recent exposome study conducted in Greece has identified that proximity to landfill waste may impact neurodevelopment in children [22].

Metabolic profiling has been utilised to characterise markers of environmental exposures [23–27] and confer valuable information regarding early life health outcomes; from preterm birth [28] and fetal growth [29] to childhood disease [30–32]. Age, sex, body morphology, and dietary intakes all play important roles in determining the urine and serum metabolome, and whilst their contributions to metabolic phenotypes are relatively well characterised in the adult population [33–42], to date there are only a few studies, of relatively small sample size, in children [43–46]. In addition, epidemiological studies that permit evaluation of the complementarity of urine and serum metabolomics data are also lacking [47].

To address this knowledge gap, metabolomic analyses of serum and urine were performed as part of the Human Early-Life Exposome (HELIX) project, which seeks to define the environmental exposome from pregnancy to childhood, to associate these with child health outcomes and to define molecular ‘omics’ markers [48]. The project gathered samples and data from six longitudinal birth cohort studies across six European countries—France, Greece, Lithuania, Norway, Spain and the UK. Analyses were conducted on biofluid samples from the HELIX subcohort of children between 6 and 11 years of age to perform molecular phenotyping including metabolomics, proteomics, transcriptomics and genomics and also to measure chemical exposure levels in order to identify molecular markers of exposure [49]. Specifically in this current study, we aim to (a) characterise the major determinants of the child metabolome, (b) define metabolite associations to demographic factors, BMI and main dietary intake habits in European children, and (c) evaluate correlation patterns and complementarity between serum and urine metabolic profiles.

## Methods

### HELIX project multilevel study design

The HELIX study is a collaborative project across six established and longitudinal birth cohorts in Europe. A multilevel study design was employed. Level 1—the entire study population of HELIX consists of 31,472 mother-child pairs which were recruited between 1999 and 2010 during their pregnancies by the six cohorts. Level 2—the HELIX subcohort consists of 1301 mother-child pairs from which exposure data, ‘omics’ molecular profiles, and child health outcomes were measured at 6–11 years of age. Level 3—panel studies with repeated sampling periods from a cohort of 150 children and 150 pregnant women to understand temporal variability of the personal exposure data [49].

### Current study sample population—the HELIX children subcohort

The children in the HELIX subcohort were followed up between December 2013 and February 2016; there were approximately 200 mother–child pairs from each of the six cohorts. Follow-up examinations for the subcohort took place either at local hospitals, primary care centres or the National Institute for Public Health (NIPH) in Oslo, during which mothers were interviewed and children checked and examined by trained nurses according to standardised operating procedures. Biological samples were also collected on the day of the examinations. Metabolic phenotypes of 1201 children’s urine and sera samples from the HELIX subcohort were generated, of which complete matching metadata listed in Table 1 were available for 1192 children as follows: Born in Bradford, UK (BiB, *n* = 199) [50]; Study of determinants of pre- and postnatal developmental, France (EDEN, *n* = 157) [51]; Infancia y Medio Ambiente, Environment and Childhood, Spain (INMA, *n* = 207) [52]; Kaunas Cohort, Lithuania (KANC, *n* = 201) [53]; The Norwegian Mother and Child Cohort Study, Norway (MoBa, *n* = 229) [54]; Mother-Child Cohort in Crete, Greece (Rhea, *n* = 199) [55]. Hence, the number of samples carried forward for data analysis was 1192.

**Table 1:** Sample population characteristics in the HELIX subcohort study

	Overall	BiB	EDEN	INMA	KANC	MoBa	Rhea
Sample *n*	1192	199	157	207	201	229	199
Female (%)	45.4	46.2	42.0	44.9	45.8	48.0	44.2
Male (%)	54.6	53.8	58.0	55.1	54.2	52.0	55.8
White European (%)	89.6	42.7	100	100	100	95.6	100
BAME (%)	10.4	57.3	0	0	0	4.4	0
Age (years)	7.4 (6.5–8.9)	6.6 (6.4–6.8)	10.8 (10.3–11.2)	8.8 (8.4–9.3)	6.4 (6.1–6.8)	8.5 (8.2–8.8)	6.5 (6.4–6.6)
BMI (z-score)	0.3 (− 0.4–1.2)	0.1 (− 0.4–0.9)	0.2 (−0.5–1.2)	0.7 (− 0.1–1.7)	0.4 (− 0.3–1.2)	0.1 (− 0.4–0.6)	0.6 (− 0.3–1.6)

NB. BAME indicates Black and Asian Minority Ethnic group. For age and BMI, median values and interquartile range in parentheses are presented

### Body mass index and food dietary frequency data

#### zBMI

During the subcohort follow-up examinations, height and weight were respectively measured with a stadiometer and a digital weight scale both without shoes and with light clothing. Height and weight measurements were converted to body mass index (BMI in kg/m^2^) for age and sex z-scores using the international World Health Organization (WHO) reference curves in order to allow for comparison with other studies [56].

#### Dietary frequency

Data on the food intake frequency of 44 food items from 11 main food groups were collected through a short food frequency questionnaire and the average number of times per week that each food item was consumed was recorded. The 11 main groups were sweets, which include chocolate (bars, bonbon, spreads, cacao), sugar, honey, jam or other sweets; meat, which includes processed meat, poultry and red meat; fish, which includes canned fish, oily fish, white fish and seafood; beverages, which include both high- and low-sugar soda, other soft and fizzy drinks; potatoes, which include also French fries; vegetables, which include both raw and cooked vegetables; dairy products, which include yogurt, cheese, milk and dairy desserts; cereal, which include bread, breakfast cereal, rice and pasta, rusks, crispy bread, rice and corn cakes; fruits, which include fruits, fresh juice, canned and dry fruits; bakery products which include biscuits, cookies and pastries; and total added lipids which include butter, margarine and vegetable oil.

### Biofluid sample collection

Urine and sera samples were collected and processed according to identical pre-defined standardised protocols across all six cohorts. Urine samples were collected by family members at home, kept in a fridge overnight and transported in a temperature controlled environment. Samples were aliquoted and frozen within 3 h of arrival at the clinics. Two urine samples, representing last night-time and first morning voids, were collected on the evening and morning before the clinical examination and were subsequently pooled to generate a more representative sample of the last 24 h for metabolomic analysis (*n* = 1107) [57]. Either the night-time void (*n* = 37) or morning void (*n* = 48) sample was analysed in cases where a pooled sample was missing.

Serum sampling: Blood was collected during the follow-up visit at the end of the clinical examination. Blood samples were drawn using a ‘butterfly’ vacuum clip and local anaesthetic and were collected into 4 mL silica plastic tubes. Samples were inverted gently for 6–7 times and spun down at 2500 g for 15 min at 4 °C. The median serum sample processing time from sample collection to freezing was 1.8 h (IQR: 1.5–2.0), and the median postprandial interval (time between last meal and blood collection) was 3.3 h (IQR: 2.8–4.0, Additional file 1: Figure S1).

### Urine metabolite NMR measurements

^1^H NMR spectroscopy was chosen for urinary analysis for several reasons: it has inherently high reproducibility [58]; urinary metabolite concentrations are high, making the relatively low sensitivity of NMR spectroscopy less of a hindrance; the data processing workflow is well established [59]. One-dimensional 600 MHz ^1^H NMR spectra of all 1192 urine samples were acquired on the same BrukerAvance III spectrometer operating at 14.1 Tesla within a period of 1 month. The spectrometer was equipped with a BrukerSampleJet system, and a 5-mm broad-band inverse configuration probe maintained at 300K. Prior to analysis, cohort samples were randomised to mitigate analytical bias, and individual samples were thawed and homogenised using a vortex mixer and centrifuged at 13,000 g for 10 min at 4 °C to remove insoluble material. Five hundred forty microliters of urine sample was mixed with 60 μL of a buffer solution (1.5 M KH_2_PO_4_, 2 mM NaN_3_, 1% deuterated 3-(trimethylsilyl)-[2,2,3,3-d4]-propionic acid sodium salt (TSP) solution, pH 7.4) and was transferred into an NMR tube (5 mm Bruker SampleJet NMR tubes). Ninety-six-sample tube well plates were kept at 6 °C in the cooled Bruker SampleJet unit. Aliquots of the study quality control (QC) sample, made from pooled urine samples from 20 individuals included in this study, were used to monitor analytical performance throughout the run and were analysed at an interval of every 23 samples (i.e. 4 QC samples per well plate). The ^1^H NMR spectra were acquired using a standard one-dimensional solvent suppression pulse sequence (relaxation delay - 90° pulse - 4 μs delay - 90° pulse - mixing time - 90° pulse - acquire FID). For each sample, 32 transients were collected into 64K data points using a spectral width of 12,000 Hz with a recycle delay of 4 s, a mixing time of 100 ms, and an acquisition time of 2.73 s. A line-broadening function of 0.3 Hz was applied prior to Fourier transformation. All ^1^H NMR spectra were automatically phased and baseline-corrected using Topspin 3.2 software (BrukerBioSpin, Rheinstetten, Germany). The ^1^H NMR urine spectra were referenced to the TSP resonance at 0 ppm. NMR spectra were imported into the MATLAB 2014a (MathWorks, Massachusetts, USA) computing environment and aligned using the recursive segment-wise peak alignment method [60], an algorithm based on cross-correlation. The study QC sample spectrum was used as a reference for spectral alignment. A single representative resonance in the spectrum was selected for each assigned metabolite, based on its presence in a high proportion of the spectra, high signal-to-noise ratio, and limited overlap with other resonances. Metabolite resonance peak areas were estimated using trapezoidal numerical integration and were corrected for local spectral baseline, and 44 metabolites were obtained using this method. Quantification was achieved for 24 metabolites; 20 metabolites were semi-quantified using a method of signal integration and quantification as previously described in Maitre et.al [57]. Probabilistic quotient normalisation [61] was used to adjust for variable urine sample dilution.

Assignment of endogenous urinary metabolites was made by reference to online databases (HMDB) [62], statistical total correlation spectroscopy (STOCSY) [63] and using ChenomxNMRsuite 7.1 profiler (ChenomxInc, Edmonton, Canada) and/or confirmed by 2D NMR experiments on a selected sample including homonuclear ^1^H-^1^H correlation spectroscopy (COSY), and ^1^H-^1^H total correlation spectroscopy (TOCSY) and ^1^H-^13^C heteronuclear single quantum coherence spectroscopy (HSQC). Spike-in experiments using authentic chemical standards were also used to confirm novel metabolite annotations. A summary of signal annotation and assignment is shown in Additional file 1: Table S1.

### Serum metabolite measurements

The Absolute*IDQ* p180 kit [64] was chosen for serum analysis as it is a widely used standardised, targeted LC-MS/MS assay, and its inter-laboratory reproducibility has been demonstrated by several independent laboratories [65]. It is increasingly employed for large-scale epidemiology studies [66] [67, 68], facilitating comparisons to thousands of metabolome profiles across other studies. Serum samples were quantified using the Absolute*IDQ* p180 kit following the manufacturer’s protocol [64] using LC-MS/MS—and Agilent HPLC 1100 liquid chromatography coupled to a SCIEX QTRAP 6500 triple quadrupole mass spectrometer. Briefly, the kit allows for the targeted analysis of 188 metabolites in the classes of amino acids, biogenic amines, acylcarnitines, glycerophospholipids, sphingolipids and sum of hexoses, covering a wide range of analytes and metabolic pathways in one targeted assay. The kit consists of a single sample processing procedure, with two separate analytical runs, a combination of liquid chromatography (LC) and flow injection analysis (FIA) coupled to tandem mass spectrometry (MS/MS). Isotopically labelled and chemically homologous internal standards were used for quantification; in total, 56 analytes were fully quantified and validated. Of the total 188 metabolites measured, 42 metabolites were measured by LC-MS/MS and 146 metabolites by FIA-MS/MS. The amino acids and biogenic amines were analysed quantitatively by LC–ESI-MS/MS, with the use of an external seven-point calibration curve based on isotope-labelled internal standards. The quantification method for all amino acids and amines was fully validated. The acylcarnitines (40), glycerophospholipids (90), sphingolipids (15), and sum of hexoses (1) were analysed by FIA-ESI-MS/MS, using a one-point internal standard calibration with representative internal standards. Metabolites were quantified (results shown in micromolar concentration units) according to the manufacturer’s protocol using the MetIDQ™ Version 5.4.8 Boron software for targeted metabolomic data processing and management. Blank PBS (phosphate-buffered saline) samples (three technical replicates) were used for the calculation of the limits of detection (LOD). The median values of all PBS samples on the plate were calculated as approximation of the background noise per metabolite signal, and 3 times this value was calculated as the LOD.

LC-MS/MS data of serum samples were acquired in 18 batches. Every analytical batch, in a 96-well plate format, is included up to 76 randomised cohort samples. Also in every analytical batch, three sets of quality control samples were included, the NIST SRM 1950 plasma reference material (in 4 replicates), a commercial available serum QC material (CQC in 2 replicates, SeraLab, S-123-M-27485) and the QCs provided by the manufacturer in three concentration levels. The NIST SRM 1950 reference was used as the main quality control sample for the LC-MS/MS analysis.

### Analytical performance of urinary and serum metabolites

Analytical performance in the urinary NMR and serum LC-MS/MS data was assessed by reference to the QC samples measured at regular intervals during the run, with 4 QC samples analysed in every 96-well plate batch. Coefficients of variation (CVs) for each metabolite were calculated based on the pooled QC for the NMR analysis and the NIST SRM 1950 for the LC-MS/MS. Moreover, for the LC-MS/MS serum analysis, the limits of detection (LODs) were also used to assess the analytical performance of individual metabolites. For the LC-MS/MS serum dataset, metabolite exclusion was based on a variable meeting two conditions: (1) CV of over 30% and (2) over 30% of the data are below LOD. Eleven out of the 188 serum metabolites detected were excluded as a result, leaving 177 serum metabolites to be used for further statistical analysis. Mean coefficients of variations across the 44 NMR detected urinary metabolites, and the 177 LC-MS/MS detected serum metabolites carried forward for data analysis were found to be 11 and 15%, respectively (Additional file 1: Tables S2 and S3).

### Statistical analyses

Metabolite concentrations were log_10_ transformed to normalise data prior to statistical analyses, and the resultant distribution of the transformed data can be found in Additional files 2 and 3. To avoid log transform of zero values, the lowest non-zero value was added to the variable distribution as a constant before log transformation. All statistical analyses were performed using R (‘The R Project for Statistical Computing’) software environment (v3.3.1) unless specified otherwise. Metabolome-wide association study (MWAS) analyses were performed using multiple linear regression models in the R package ‘base’. Linear regression models were fitted for each metabolite with concentration as the outcome variable. Covariates included in the regression models were batch, run order, sex, age, zBMI and dietary intake habits of the 11 food groups; in addition, urine data models were adjusted for sampling type (night only, morning only or pooled sample) and the serum data models were adjusted for postprandial interval. Regression models were computed separately for each individual cohort and meta-analysis was used to combine the effect size estimates using a fixed-effect inverse variance weighting from the six cohorts with the R package ‘meta’, and *I*^2^ statistics were used to assess the heterogeneity in the effect estimates between the cohorts. Bonferroni correction (*n* = 177 for serum data, *n* = 44 for urine data) was applied throughout to account for multiple test comparisons (*p* value threshold = 1.1 × 10^−3^ for urine and 2.8 × 10^−4^ for serum metabolites). For variance decomposition, analysis was performed using a partial *R*^2^ approach, the variance in the urinary and serum data was partitioned according to the following 5 main categories: pre-analytical, analytical, demographic, dietary and cohort/country. The analysis was performed on each of the 44 urinary metabolites and 177 serum metabolites. In addition to the covariates used in the MWAS analyses—batch (analytical), run order (analytical), time of sampling (urine pre-analytical), postprandial interval (serum pre-analytical), sex (demographic), age (demographic), BMI z-score (demographic) and dietary intake frequencies; ethnicity (demographic), and serum and urine sample processing time variables (pre-analytical) were also included in the respective serum and urine variance decomposition analyses. For principal components analysis, metabolite data were also mean-centred and univariate scaled prior to PCA modelling. For serum and urine metabolic pairwise correlation analyses, data were pre-adjusted for analytical and pre-analytical variables and Pearson’s correlation coefficients were calculated. Serum correlation networks were drawn using Cytoscape (version 3.5) software [69] and the MetScape plugin application (version 3) [70]. Additionally, we have examined the impact of applying alternative data transformation and imputation strategies on the MWAS analysis results. To this end, Box-Cox transformation [71] was used in conjunction with QRILC imputation (quantile regression approach for left-censored missing) [72] and the modelled results are shown in Additional file 1: Tables S12–S15. Box-Cox transformation and QRILC imputation were performed respectively using R packages ‘MASS’ and ‘imputeLCMD’.

## Results

Characteristics of the study population included in this analysis (*n* = 1192) are shown in Table 1. Around 200 children from each of the six cohorts participated in this study (54.6% male, 45.4% female), and the vast majority of the sample population were of White-European background with the notable exception of BiB (UK) where many were of Black and Asian Minority Ethnic group, mainly of South Asian origin. There were also significant age differences between the cohorts, with the children from the EDEN cohort being the oldest (median age in EDEN was 10.8 years whilst median ages in KANC, BiB, and Rhea were 6.4–6.6 years). In addition, there were substantial differences between the cohorts in BMI z-score and across dietary intake habits in the 11 food groups (Tables 1 and 2).

**Table 2:** Dietary intake of 11 main food groups

	Overall	BiB	EDEN	INMA	KANC	MoBa	Rhea
Cereal	18.5 (12.5–26)	17 (13.1–23.5)	21.5 (13–27.5)	17 (11.4–24.1)	14 (9.3–22)	25 (19–29.6)	16 (12.1–23.5)
Meat	7.5 (5.1–10)	6 (4–9)	7.5 (5.5–10)	8 (7–12)	7.1 (5.1–10)	7.5 (5–10.5)	6.5 (5–7.6)
Fish	2 (1.1–3.5)	2 (1–3.3)	2.1 (1.5–3)	3.6 (2.4–5)	1.1 (0.4–1.6)	2.6 (1.6–5)	1.5 (1–2)
Dairy	19.8 (12.5–27.6)	24 (17.2–31.6)	24 (15–31)	18.1 (13.8–25.8)	11 (8–17)	20 (12.3–27)	22.5 (15.1–28.5)
Lipids	4.5 (1–8.5)	7 (4–10)	6 (3.1–9)	1 (0.3–3.1)	7 (4–11)	7.5 (4–15.5)	1 (0.1–3)
Potatoes	3.5 (2–4)	4 (3.1–6)	3.1 (1.5–4)	3.5 (2–4)	3.1 (3–5.6)	3.1 (1.1–3.1)	3.5 (2–4)
Vegetables	6.5 (4–10)	6 (4–10)	8 (4–11)	6 (3–8.5)	6 (3.5–8.5)	8.5 (6–14)	6.5 (4–10)
Fruits	8.8 (5.9–18)	15.5 (10–21)	6.6 (3.3–13.6)	7.5 (3.7–13.2)	7.4 (3.8–10)	14 (8.6–21)	8.5 (6.2–13.5)
Sweets	6.6 (3.5–10)	6.6 (3.6–10)	8.5 (5–14)	4.5 (2–8.1)	9.5 (7–15.5)	5 (3–7.5)	6 (3.3–7.5)
Bakery products	4 (1.5–6.5)	4 (2–7.5)	5.6 (2–8)	4 (3–6.5)	4 (2–6.5)	1.5 (1–2)	6 (4–8.5)
Beverages	0.5 (0–1)	0.5 (0–1.8)	1 (0.3–3)	0.5 (0–1.3)	0.5 (0.1–1)	0.6 (0.1–1.1)	0.1 (0–0.5)

Data represent portion consumed per week. Median values with interquartile range given in parentheses or percentages as indicated

In our study, ^1^H NMR spectroscopy and targeted LC-MS/MS were respectively used to perform metabolic profiling of the urine and serum samples. Estimates of the concentrations for urinary metabolites using NMR spectroscopy (μmol/mmol of creatinine) are provided in Additional file 1: Table S4 and for serum metabolite measurements using the LC-MS/MS Absolute*IDQ* p180 assay (μmol/L) in Additional file 1: Table S5.

### Metabolic differences between cohorts

**Fig. 1:**
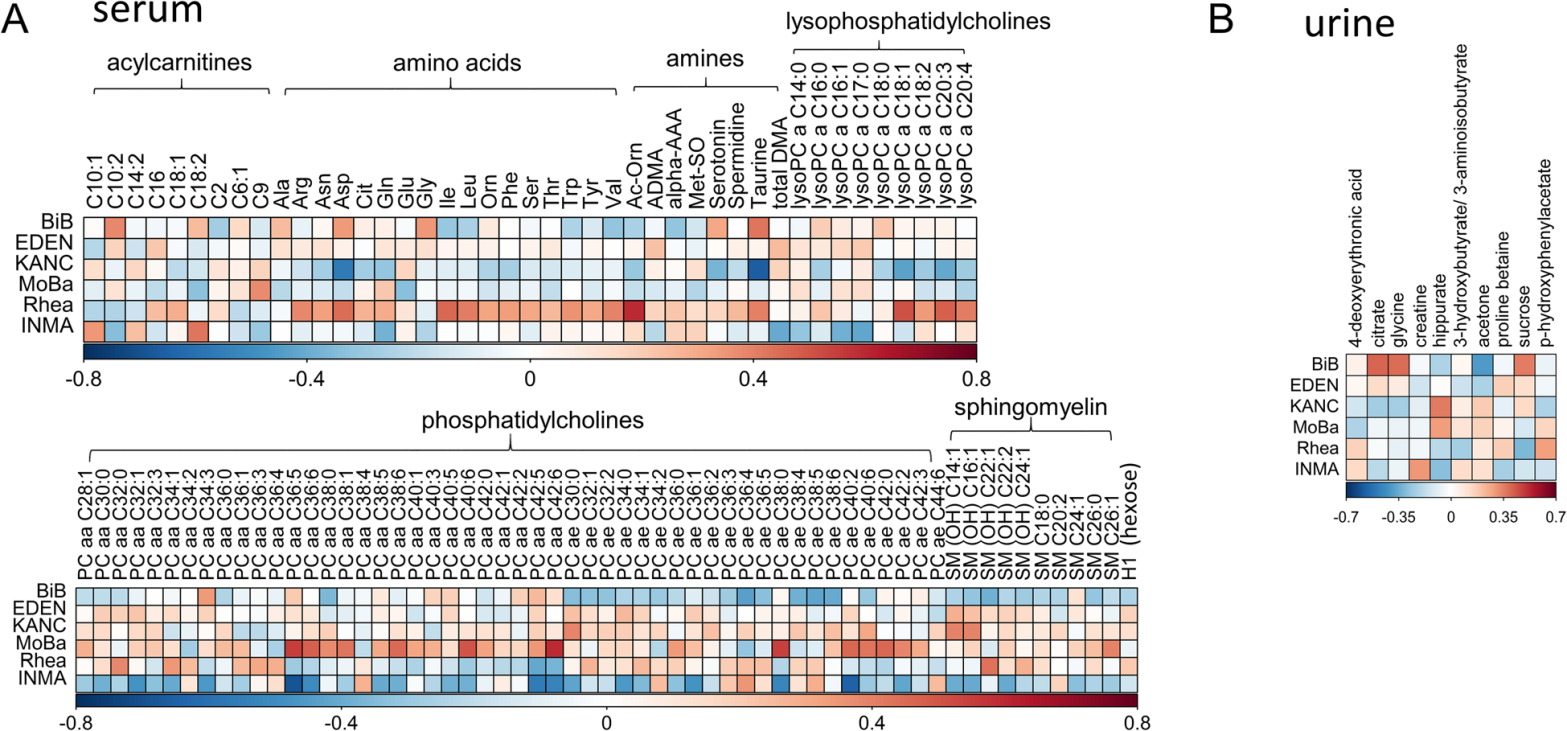
Metabolic differences between the six cohorts. **a** Serum metabolites. **b** Urine metabolites. Colour represents standardised mean difference between cohorts; blue—metabolite levels lower than average, and red—metabolite levels higher than average. *P* values were assessed by ANOVA, and significant metabolites after multiple testing correction are shown. Using multiple linear regression models metabolic data were pre-adjusted for analytical batch and run order, age, sex, zBMI, frequency of weekly dietary intake of the 11 food groups, and a sampling type in the case of urine and postprandial interval in the case of serum, prior to ANOVA analysis. BiB (UK), EDEN (France), KANC (Lithuania), MoBa (Norway), Rhea (Greece), INMA (Spain)

Differences in metabolite concentrations between cohorts were assessed by ANOVA after pre-adjusting for covariates through linear regression models. Metabolites with *p* values below the significance threshold after Bonferroni correction (*p* value threshold = 1.1 × 10^−3^ for urine and 2.8 × 10^−4^ for serum metabolites) are shown in Fig. 1. A large number of metabolites, 104 out of 177 serum metabolites and 10 of the 44 urine metabolites measured, were found to be significantly different between cohorts. In particular, serum amino acid levels were frequently found highest in the Rhea cohort, whilst a disproportionally high number of serum glycerophospholipid species were found to be most abundant in the MoBa cohort samples. Given the stark differences in the metabolic phenotypes between cohorts, we decided to perform stratified analyses followed by meta-analysis to combine the effect estimates from the six individual cohorts in many of the subsequent analyses.

### Pre-analytical factors

None of the 177 serum metabolites were identified from meta-analysis to be significantly affected by serum sample processing time after adjusting for covariates and stratifying by country. Similarly, none of the 44 urinary metabolites were found to be associated with sample processing time. Thus, in subsequent analyses, urine and serum processing time were not included as covariates.

**Fig. 2:**
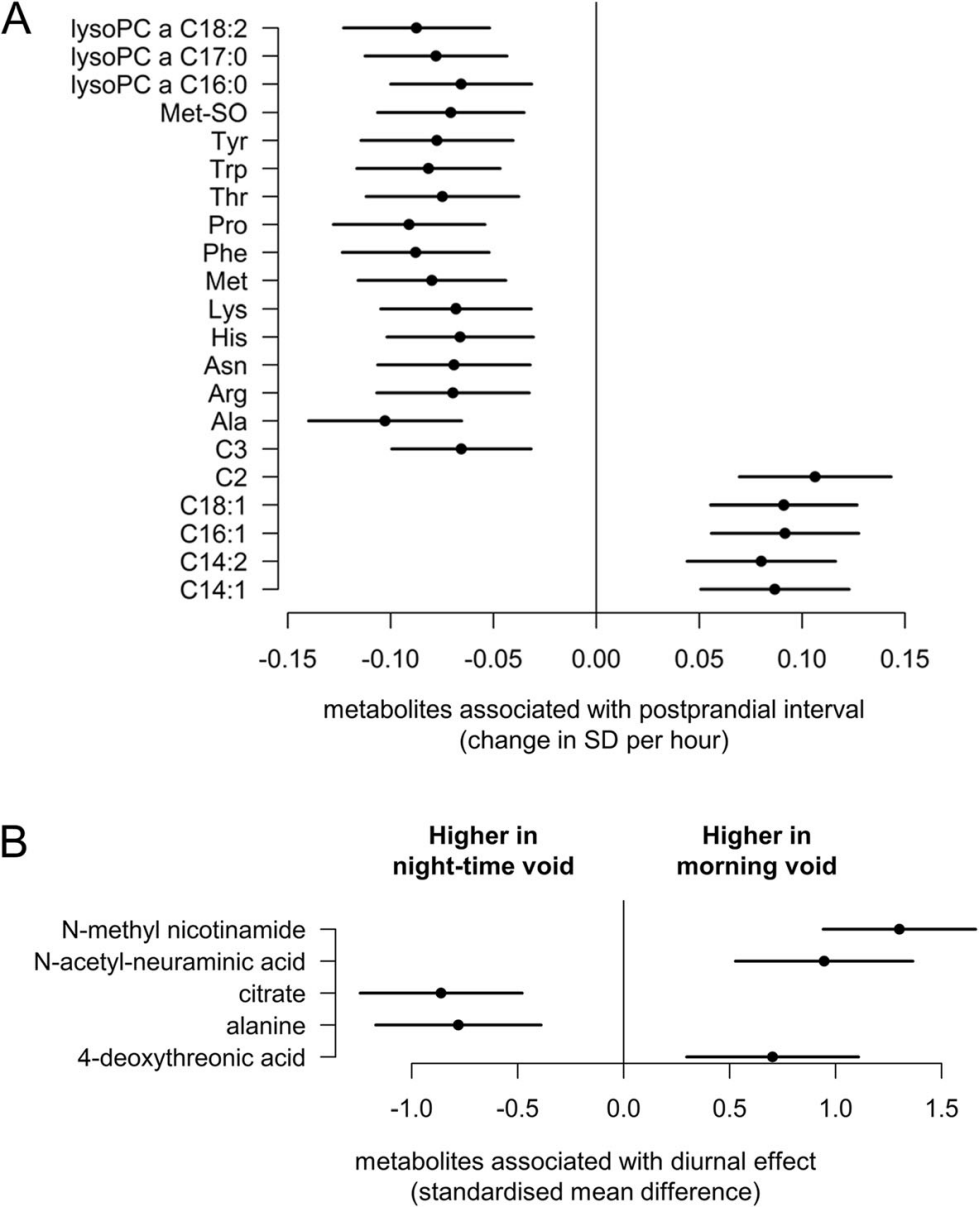
Pre-analytical factor effects on the children’s metabolome. **a** Postprandial effects on serum metabolites (adjusted for age, sex, zBMI)—meta-analysis after stratifying by cohorts with estimates representing the change in metabolite SD per hour postprandial and error bar indicating 95% confidence interval. **b** Diurnal effects on urine metabolites. Only *t* test adjusted *p* < 0.05 are shown (*n* = 48 for morning and *n* = 37 for night samples). The estimates indicate the standardised mean differences between the morning and night samples, with the error bars indicating the 95% confidence intervals. Metabolites found higher in the morning void samples are shown as positive and metabolites found higher in night-time void samples are shown as negative

The majority of serum samples were collected 3 to 4 h postprandial (median was 3.3 h with IQR: 2.8–4.0), and there were no major differences in postprandial interval between the cohorts (Additional file 1: Figure S1). Postprandial effects could be observed in 21 out of 177 metabolites: 11 amino acids, one biogenic amine, two short-chain acylcarnitines, four long-chain acylcarnitines and three lysophosphatidylcholine species were found to be associated with postprandial interval (Fig. 2a). The 11 amino acids were negatively associated whilst the four long-chain acylcarnitines were positively associated with postprandial interval.

Comparing the urinary metabolite levels of night-time void (*n* = 38) and morning void (*n* = 48) samples, we found alanine and citrate concentrations to be elevated in the night-time void samples and *N*-methyl nicotinamide, *N*-acetyl-neuraminic acid and 4-deoxythreonic acid to be higher in the morning void samples (Fig. 2b).

### Demographic factors and BMI for the HELIX children

Both urinary and serum creatinine levels (Additional file 1: Figures S3, S4 and Table S6) were found to be significantly associated with age after adjusting for multiple testing using Bonferroni correction. An increase of 1 year in the child’s age was associated with increases of 0.39 standard deviation (SD) in urinary creatinine level (95% CI 0.26 to 0.53) and 0.30 SD in serum creatinine level (95% CI 0.17 to 0.43). A positive association between creatinine concentration and age was identified as a common phenotype amongst our six different study cohorts (Additional file 1: Figures S3 and S4); effect sizes between urine creatinine level and age were 0.40 SD/year for BiB, 0.27 SD/year for EDEN, 0.35 SD/year for KANC, 0.33 SD/year for MoBa, 0.84 SD/year for Rhea and 0.45 SD/year for INMA. No other urine or serum metabolites measured were associated with age.

**Fig. 3:**
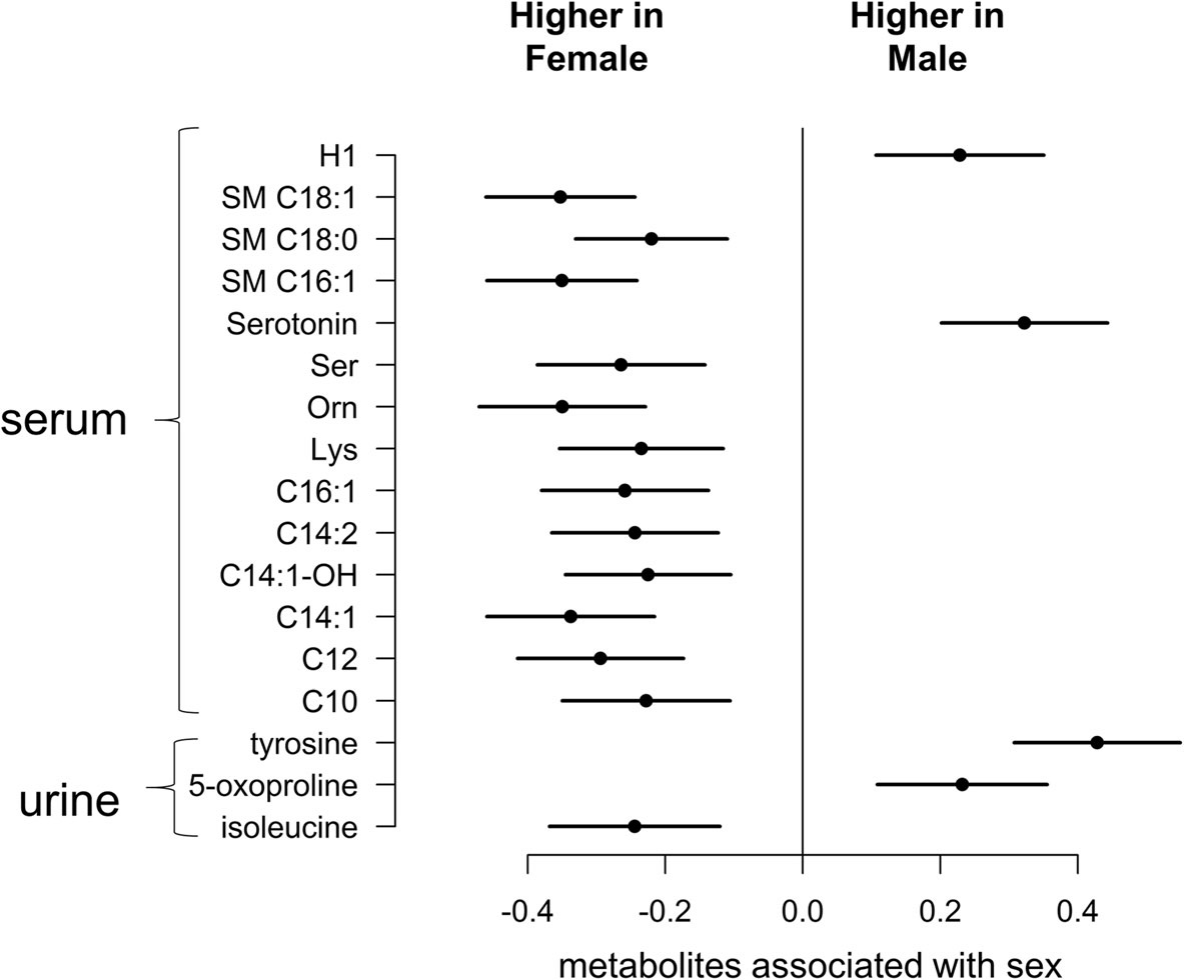
Sex associations with ^1^H NMR urine and serum metabolites in children—meta-analysis after stratifying by cohorts. Regression models were adjusted for covariates, and Bonferroni correction was used to adjust for multiple testing. The estimates represent the metabolite standardised mean difference between males and females with the error bars indicating the 95% confidence intervals. Metabolites found higher in male children are shown as positive, and metabolites found higher in female children are shown as negative

Metabolic associations with sex, adjusted for covariates and multiple testing, are shown in Fig. 3. Variation in effect size between cohorts was assessed using *I*^2^ statistic, which measures the percentage of variation across cohorts that is due to heterogeneity rather than chance. Fifteen out of 18 urine or serum metabolites identified as associated with sex have *I*^2^ < 50% (Additional file 1: Table S7). Urinary isoleucine was found at lower concentrations (− 0.24 SD lower; 95% CI − 0.37 to − 0.12) while 5-oxoproline (0.23 SD higher; CI 0.11 to 0.36) and tyrosine (0.43 SD higher; CI 0.31 to 0.55) were higher in males. Amongst the serum metabolites, the neurotransmitter serotonin (0.32 SD higher; CI 0.20 to 0.44) was found to be higher in males while serine (− 0.26; CI − 0.39 to − 0.14), lysine (− 0.24; CI − 0.35 to − 0.12), ornithine (− 0.35; CI − 0.47 to − 0.23), putrescine (− 0.21; CI − 0.33 to − 0.10), six median-to-long chain acylcarnitines (C10, C12, C14:1, C14:1–OH, C14:2 and C16:1) and three sphingolipids (SM C16:1, SM C18:0, SM C18:1) were found higher in females.

**Fig. 4:**
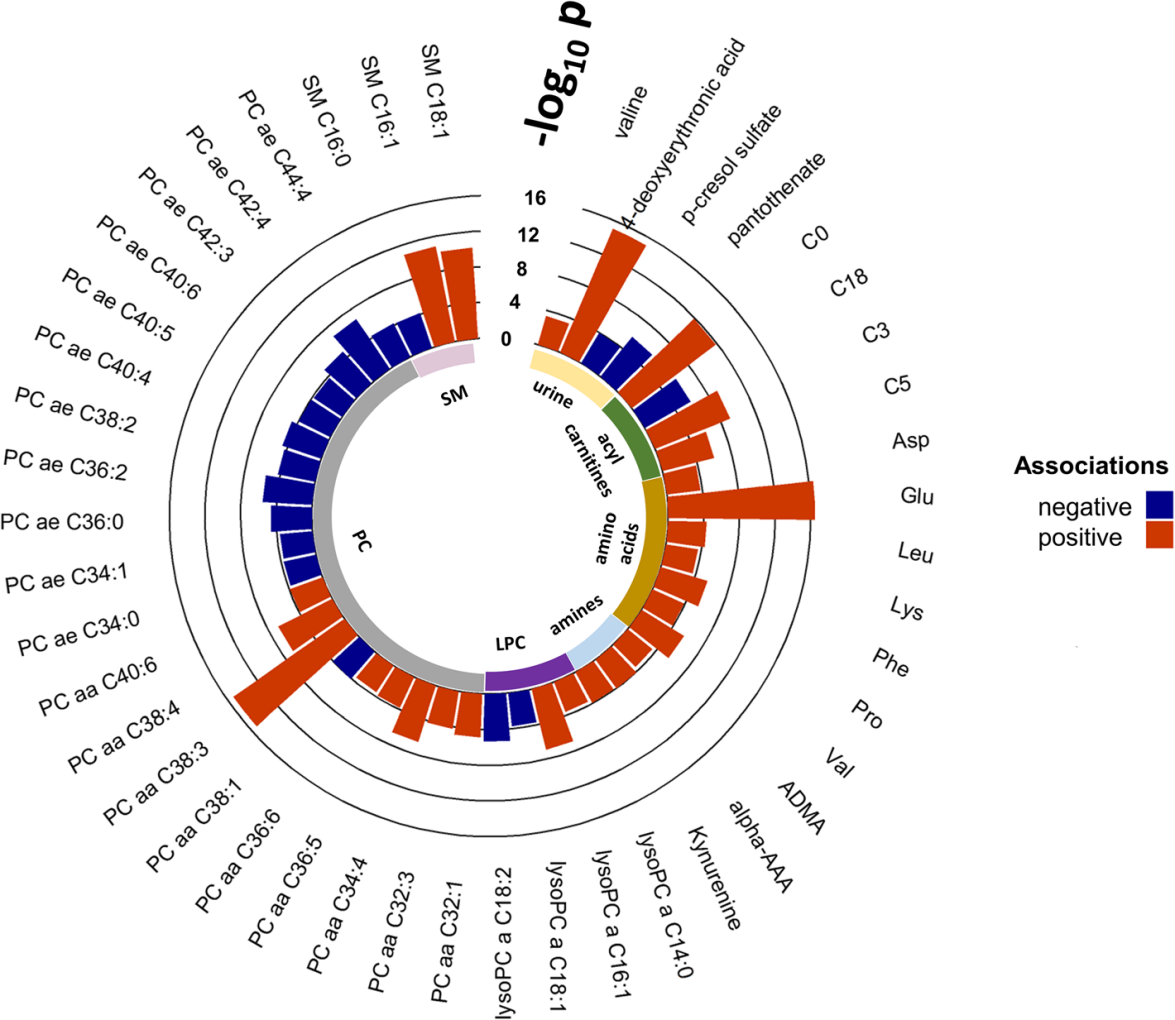
Urine and serum metabolites associated with BMI z-score—meta-analysis after stratifying by cohorts. Regression models were adjusted for analytical batching, postprandial effect (for serum), sampling (urine), age, sex and dietary intakes of the 11 main food groups

Based on regression models adjusted for covariates, we found 45 urine or serum metabolites to be associated with BMI z-score and 44 of the 45 associations have *I*^2^ < 50% (Fig. 4 and Additional file 1: Table S8). Urinary 4-deoxyerythronic acid (metabolite SD per unit zBMI: 0.21; 95% CI 0.16 to 0.26) and valine (BCAA, metabolite SD/zBMI: 0.09; CI 0.04 to 0.15) were positively associated with BMI z-score, and urinary *p*-cresol sulphate (a microbial metabolite and uremic toxicant [73], metabolite SD/zBMI: − 0.10; CI − 0.16 to − 0.05) and pantothenate (vitamin B_5_—required for synthesis of coenzyme A, metabolite SD/zBMI: − 0.12; CI − 0.17 to − 0.07) were negatively associated with BMI z-score. Positive associations between urine 4-deoxyerythronic acid and valine levels and zBMI could be observed consistently in five of the six different study cohorts with the exception of MoBa (Additional file 1: Figures S5 and S6); effect sizes between urine 4-deoxyerythronic acid level and zBMI were 0.25 SD/unit score for BiB, 0.25 SD/unit score for EDEN, 0.25 SD/unit score for KANC, 0.00 SD/unit score for MoBa (not significant), 0.22 SD/unit score for Rhea and 0.19 SD/unit score for INMA. Interestingly, children from MoBa have the lowest BMI z-score amongst the six cohorts (Table 1).

Amongst serum metabolites, significant positive associations with BMI z-score included free carnitine, (metabolite SD/zBMI: 0.18; CI 0.13 to 0.24), short-chain acylcarnitines (C3, C5), seven amino acids including glutamate, BCAAs valine and leucine and sphingolipids (SM C16:0, SM C16:1, SM C18:1). A large number of phosphatidylcholine species (20) and four lysophosphatidylcholines (lysoPC a C14:0, lysoPC a C16:1, lysoPC a C18:1, lysoPC a C18:2) were also found to be strongly associated with BMI z-score in the study (Fig. 4 and Additional file 1: Table S8). Again, associations between serum metabolites and zBMI could be observed consistently in our study cohorts, for example both serum glutamate (Additional file 1: Figure S7) and carnitine (Additional file 1: Figure S8) levels were positively associated with zBMI in all six cohorts.

### Dietary intake

**Fig. 5:**
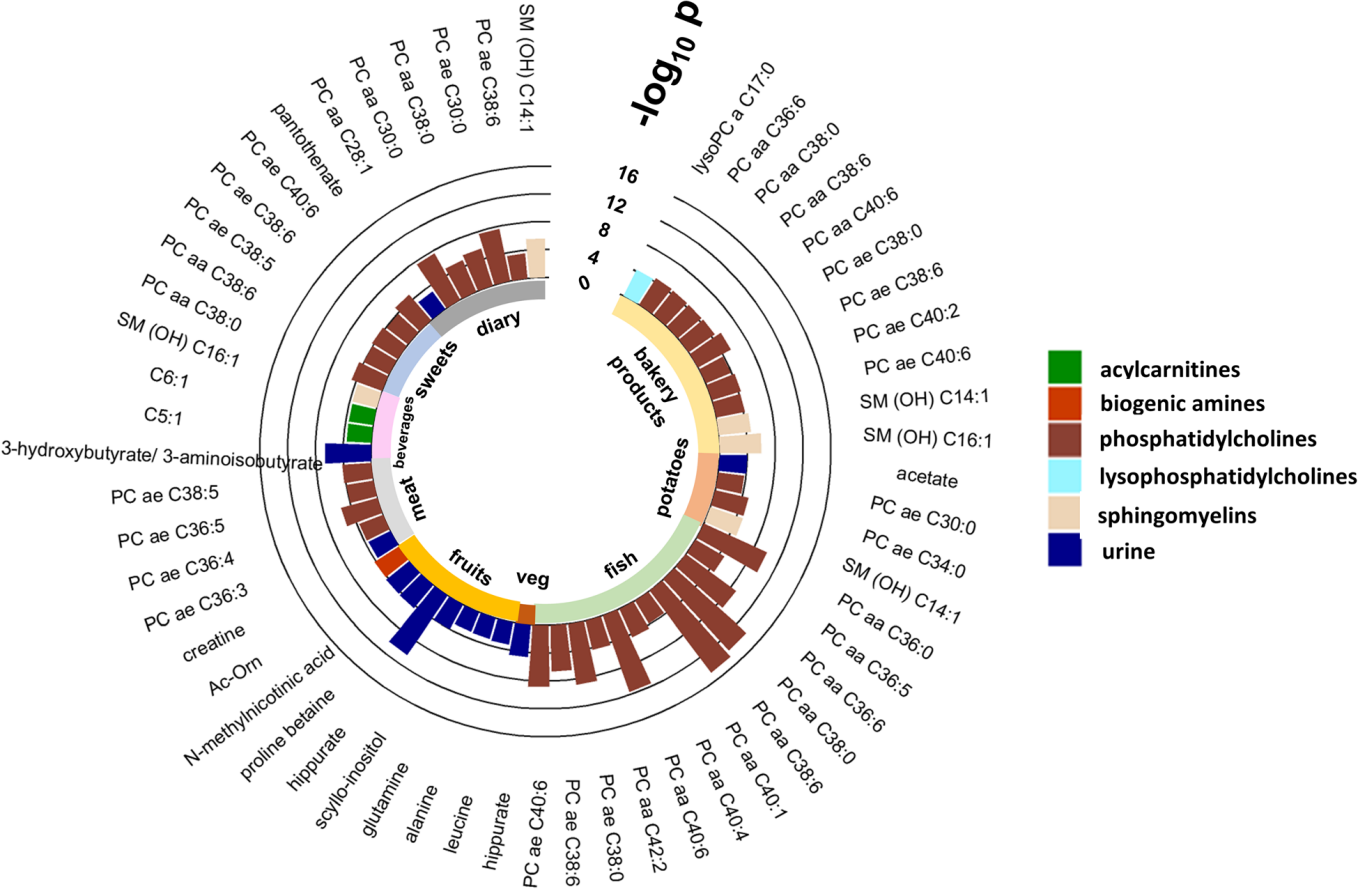
Metabolites associated with dietary intake frequencies (weekly). Weekly dietary frequency intake data of the 11 main food groups (cereal, meat, fish, dairy, lipids, potatoes, vegetables, fruits, sweets, bakery products, beverages) were collected via food frequency questionnaire, and multiple linear regression analysis followed by meta-analysis were performed on each metabolite—dietary factor pair. Regression models were adjusted for analytical batching, postprandial effect (for serum), sampling (urine), age, sex and zBMI score

Figure 5 and Additional file 1: Table S9 summarise the significant urine and serum metabolite associations with the 11 dietary food group intake after adjusting for multiple testing (*p* value threshold = 1.1 × 10^−3^ for urine and 2.8 × 10^−4^ for serum metabolites) and covariates including analytical batch and run order, age, sex, BMI z-score and postprandial interval for serum and urine sampling type for urine models. We identified 57 diet-metabolite associations and 40 of the 57 associations have *I*^2^ < 50%.

For urinary metabolites, we identified creatine to be positively associated with meat intake (SD per portion per week: 0.025; 95% CI 0.012 to 0.039). Hippurate was positively associated with both fruit (SD per portion per week: 0.026; 95% CI 0.018 to 0.034) and vegetable consumption (SD per portion per week: 0.021; 95% CI 0.011 to 0.031). Proline betaine, *N*-methylnicotinic acid and *scyllo*-inositol were positively associated with fruit intake, whilst glutamine, alanine and leucine were negatively associated with fruit intake. In addition, pantothenate and acetate were respectively found positively associated with dairy and potato intake.

For serum metabolites, we found 12 glycerophosphatidylcholine species to be associated with fish consumption (Fig. 5), 4 glycerophosphatidylcholine species (PC ae 36:3, PC ae 36:4, PC ae 36:5 and PC ae 38:5) to be positively associated with meat consumption and 5 glycerophosphatidylcholine species (PC aa C38:0, PC aa C38:6, PC ae C38:5, PC ae C38:6, PC ae C40:6) to be negatively associated with sweet consumption. In addition, we found acetylornithine to be positively associated with fruit intake, and two acylcarnitines (C5:1, C6:1) and one sphingolipid (SM (OH) C16:1) to be negatively associated with beverages (soft and fizzy drinks).

### Variance decomposition analysis of LC-MS/MS serum and NMR urine metabolic profiles

**Fig. 6:**
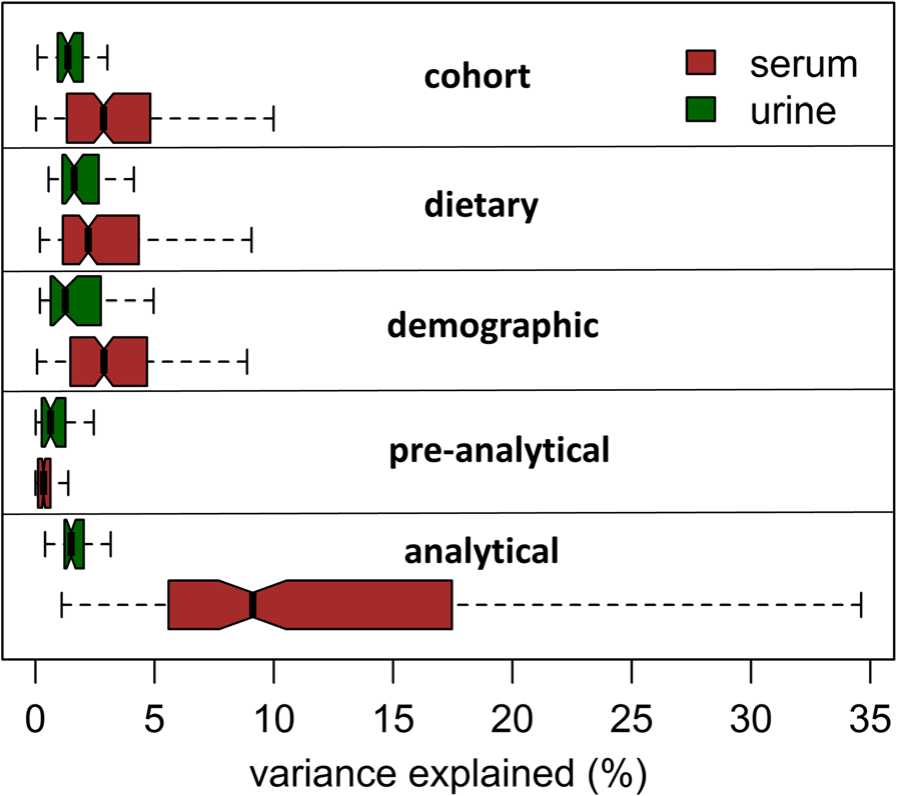
Variance decompositions of LC-MS/MS serum and NMR urine metabolic profiles. Using a partial *R*^2^ approach, regression models were performed on each of the 44 urinary metabolites and on each of the 177 serum metabolites. Variables included in the model: batch (analytical), run order (analytical), time of sampling (urine pre-analytical), postprandial interval (serum pre-analytical), sample processing time (pre-analytical), sex (demographic), age (demographic), BMI z-score (demographic), ethnicity (demographic), 11 dietary intake frequencies (dietary) and cohort

Using principal components analysis, we found that metabolites in LC-MS/MS serum metabolic profiles were inherently more collinear when compared to NMR urine profiles; only 6 principal components were required to describe half of the variance in the 177 serum metabolites as opposed to 12 principal components required to describe the same proportion of the variance in the 44 urinary metabolites (Additional file 1: Figure S9). Secondly, as metabolic profiles often capture information derivable from various sources that may be analysis-specific or individual-specific, we performed variance decomposition analysis to discover and compare the volume of information contained in the two metabolic datasets that were attributable to the various factors. Using a partial *R*^2^ approach, we partitioned the variance in the urinary and serum data according to the following 5 main categories: pre-analytical, analytical, demographic, dietary and cohort/country. The analysis was performed on each of the 44 urinary metabolites and on each of the 177 serum metabolites, and Fig. 6 illustrates the distributions of the percentages of variance explained by the 5 categories. Our data indicate that whilst analytical biases accounted for only a small fraction (median of 1.5%) of the explained variance in the NMR urinary profile, they accounted for a much larger portion of the explained variance (median of 9.1%) in the LC-MS/MS serum profile. Dietary information accounted for the largest proportion of the explained variance in the urinary metabolic profile (median of 1.6%), and overall, we found that demographic, dietary and information about country of origin are better reflected in the serum dataset, as these factors together explain a median of 9.0% amongst serum metabolites versus a median of 5.1% amongst urine metabolites (breakdown by individual metabolite can be found in Additional file 1: Tables S10 and S11).

### Serum and urine metabolic pairwise correlations

**Fig. 7:**
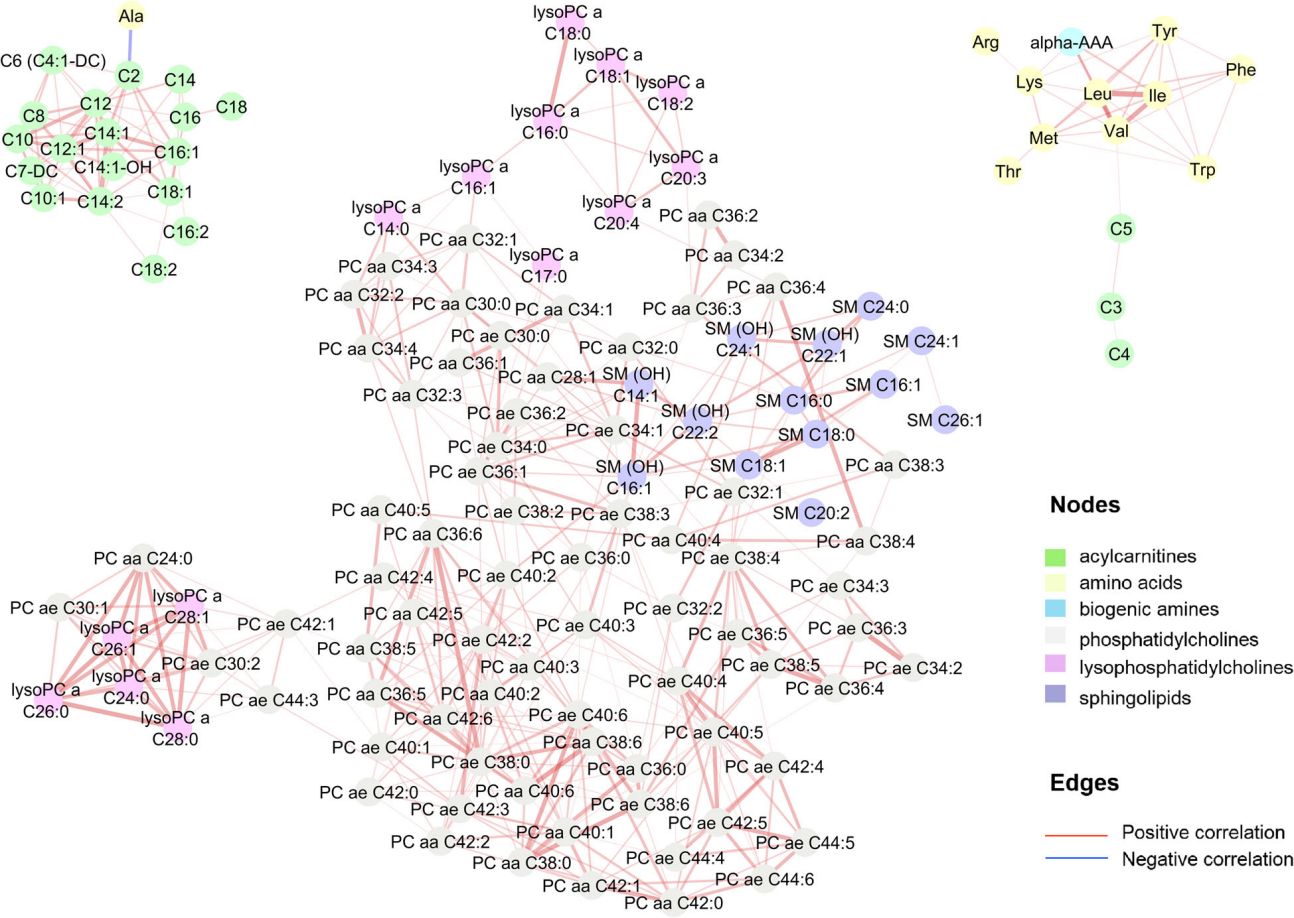
Serum metabolic correlation network diagram generated using MetScape (Cytoscape) based on metabolite pairwise correlations (“edge”) either < −  0.5 or > 0.65

**Fig. 8:**
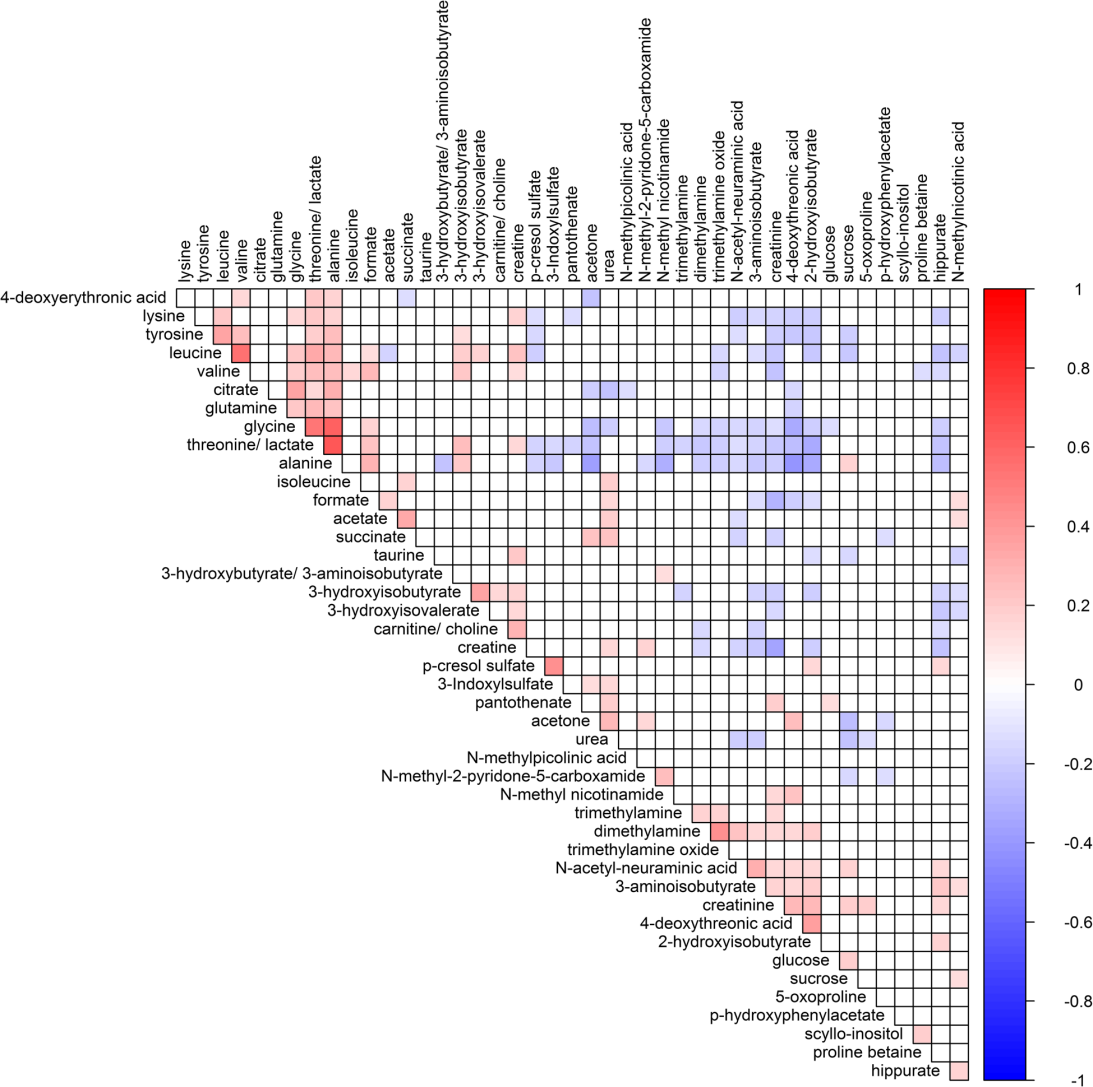
Urinary metabolic correlation heatmap diagram. Colour represents Pearson correlation coefficients and only significant correlations after Bonferroni correlations (*p* value threshold = 5.3 × 10^−5^) are shown

Metabolite inter-correlations often convey biological pathway information; thus, metabolite pairwise correlation analyses were performed separately for serum and urine datasets. Significant correlations were observed between serum metabolites which belong to the same compound classes (Fig. 7), and in particular, strong correlation clusters are found for glycerophospholipids species (maximum Pearson’s correlation coefficient *r* = 0.94), amino acids (maximum *r* = 0.97) and acylcarnitines (maximum *r* = 0.88). Other notable correlations included positive correlations between valine, leucine and isoleucine (all BCAA, *r* > 0.92), alpha-AAA (α-aminoadipic acid) with BCAA and lysine, positive correlations between valine and short chain acylcarnitines (C5, C3, C4, *r* = 0.65 between valine and C5) and negative correlations between alanine and acetylcarnitine (C2, *r* = − 0.54). Significant positive correlations between urine metabolites are shown as a heatmap in Fig. 8 (*p* value threshold of 5.3 × 10^−5^). Positive correlations included leucine with valine (*r* = 0.56), acetate with succinate (*r* = 0.32), formate with acetate (*r* = 0.17), trimethylamine oxide and dimethylamine (*r* = 0.44), 3-indoxylsulfate and *p*-cresol sulphate (*r* = 0.43), alanine and glycine and threonine/lactate (*r* = 0.52–0.65), 4-deoxyerythronic acid with alanine (*r* = 0.17) and threonine/lactate (*r* = 0.21), and creatine with carnitine/choline (*r* = 0.30). Significant negative correlations included 4-deoxythreonic acid with the following amino acids: threonine/lactate, alanine, tyrosine, glutamine and glycine (*r* = − 0.17 to − 0.42). Pairwise correlation between metabolite concentrations across the two biological fluid types were also examined (Additional file 1: Figure S10, *p* value threshold of 6.4 × 10^−6^). Significant correlations were found in 391/7788 serum-urine metabolite pairs. Significant positive correlations were found in the cases when a metabolite has been measured in both urine and serum. Specifically creatinine (*r* = 0.39), glycine (*r* = 0.35), alanine (*r* = 0.29), valine (*r* = 0.18), serum carnitine and urine carnitine/choline (*r* = 0.23), and serum threonine and urinary threonine/lactate (*r* = 0.26) are all individually strongly correlated across the two biological fluid matrices. Other notable correlations include serum threonine with urine 4-deoxyerythronic acid (*r* = 0.31), which is consistent with the proposition that threonine is the main source of 4-deoxyerythronic acid [74]. Urine *N*-methylnicotinic acid was correlated (*r* = 0.23) with serum Ac-Orn (acetylornithine), and additionally, we also found urine acetone and 4-deoxythreonic acid to be positively associated with multiple serum acylcarnitines, while urine alanine was negatively associated with multiple serum acylcarnitines (Additional file 1: Figure S10). Amongst the 391 significant serum-urine metabolite pairs, the median correlation *r*^*2*^ was 2.7% whilst across all 7788 serum-urine metabolite pairs the median correlation *r*^*2*^ was only 0.15% indicating that, even if a subset of serum-urine metabolic correlations are significant, information contained in our urine and serum profiles was largely orthogonal to one another.

## Discussion

Utilising two reproducible and well-characterised metabolic profiling platforms, ^1^H NMR spectroscopy and LC-MS/MS, we have characterised the urine and serum metabolic phenotypes in European children from six cohort populations representing different demographic and sample characteristics. Little is known regarding the normal concentration ranges of urinary and serum metabolites in healthy European children at present, and in this study, we have used a sample size of approximately 1200 individuals spread across six European countries and embedded the work in a population with rich metadata on diet, anthropometry and environmental exposure. ^1^H NMR spectroscopy and targeted LC-MS/MS (the Absolute*IDQ* p180 kit) were chosen for the analysis of urine, and serum samples correspondingly in this study, as they offer good sensitivity, broad dynamic range and metabolite coverage, are widely applied and have been used previously for epidemiological studies in the respective bio fluids [75].

### Sample handling and pre-analytical effects

Sample handling in such a large population and across six different centres would be expected to impact on metabolite levels. Stability of serum metabolites are considered lower when compared to those found in urine, and it has been reported that concentrations of many blood metabolites are altered by 12 h pre-storage delay at room temperature [76]. Thus, great care was taken when the study sample collection protocol was developed to help ensure that the sample processing time was kept short (< 2 h). Two separate studies have previously found that urine or serum samples stored at 4 °C for up to 24 h before being frozen were comparable to those frozen immediately [77, 78], and in our study, we have confirmed that neither urine nor serum sample processing time appear to bias our subsequent data analysis. Also, the design of the urine sample collection benefited from our previous pilot work [57] and we took advantage of a pooled sample design, combining the last sample before bedtime with the first morning void sample in the following day, to reduce diurnal variations. Morning or night void samples were only included in analyses as replacements for the pooled samples when pooled samples were missing (7% of the total). Levels of several metabolites, including citrate and *N*-methyl nicotinamide, were found to be significantly different between morning or night-time void samples; these are consistent with findings from our earlier pilot panel study which examined the diurnal and day-to-day variability of urine sampling [57]. Whilst fasting-state samples reduce temporal within-day sampling variability compared to non-fasted samples [75], such sample collections are not always feasible, as was the case for the HELIX project. Thus, most of the serum samples analysed were from non-fasting states with a median postprandial period of 3.3 h, and we have found large number of amino acids and acylcarnitines to be associated with postprandial intervals. Similarly, in a previous study of healthy female volunteers [79], using the Absolute*IDQ* p180 kit, significantly altered postprandial concentrations of amino acids and acylcarnitines were reported, likely as a result of changes in fatty acid oxidation and ketogenesis.

### Demographic factors, BMI and the child metabolome

Overall, we found the serum metabolite concentrations from the HELIX children population to be remarkably similar to reference values obtained in a study of healthy French adults [66]. However, there are some notable differences; for example, the serum creatinine level is lower in the HELIX children compared with adult populations, probably reflecting differences in lean muscle mass between adults and children [80]—a well-studied phenomenon [81] that was replicated in our study. Likewise, the urinary creatinine level was lower in the HELIX children population compared to reference values for adult populations [82, 83], and our cohort-stratified regression models also identified both urinary and serum creatinine to be positively associated with a child’s age, reaffirming creatinine as a valid indicator of muscle development in children [81, 82, 84].

Body anthropometry is an important predictor of molecular profiles and is of intense interest for disease risk stratification in epidemiological studies. The standardised BMI z-score calculated for a given age and sex has been established as a reliable measure in accessing obesity burden in child populations [85]. We observed positive associations between urinary and serum BCAAs and standardised BMI z-score, which have previously been reported in other children or young adult populations [43, 86, 87]. BCAAs are important nutrient signals [88], and increased circulating BCAAs levels have been suggested to predict future insulin resistance [43] as well as increased cardio-metabolic risk independent to adiposity in young adults [86]. Also, we identified two sphingolipids (SM C16:1 and SM C18:1) to be both higher in females and positively associated with BMI z-score, possibly reflecting differences in body fat composition and physical development between boys and girls. Also, two of the lysophosphatidylcholines (lysoPC a C16:1, lysoPC a C18:1) associated with BMI z-score in this study have recently been shown to be correlated to infant birth weight [89]. Moreover, out of the 41 serum metabolites found to be associated with BMI z-score in our HELIX children cohort, 14 metabolites (including kynurenine, glutamate, lysoPC a C18:1, lysoPC a C18:2) have also previously been reported in the EPIC study in an adult population, where the Absolute*IDQ* p180 kit was also used [90], demonstrating that many serum metabolic associations with BMI observed in adulthood can also be found in childhood.

A key finding of our study was novel evidence for a positive association between urinary 4-deoxyerythronic acid and child BMI z-score, a threonine catabolite [91, 92] found elevated during pregnancy [93]. Whilst very little is currently known about the biology of 4-deoxyerythreonic acid, it is present and has been found to be inversely associated with age in adults [27, 74, 94], and higher levels of this and related metabolites have been observed in children with early onset type I diabetes [95]. Threonine is an essential amino acid, and threonine dehydrogenase has been reported as a relatively minor (~ 10%) contributor to threonine oxidation in humans when compared to other species (up to 80%) [77]—indicating that exogenous sources or symbiotic microbial metabolism may be playing an important role in 4-deoxyerythronic acid exposure. Interestingly, it has been reported that formula-fed infants have a lower capacity to oxidise threonine than do infants fed breast milk [96] and that catabolism of threonine can lead to methylglyoxal production which contributes to the pathophysiology of obesity and diabetes [97] and can reduce health span in model systems [98]. Urinary 4-deoxyerythronic acid was found positively associated with child BMI in five of the six participating cohorts, with the exception of MoBa which has the lowest BMI z-score distribution amongst the six cohorts. It is possible that 4-deoxyerythronic acid association to BMI is more discernable in overweight populations. We report herein a correlation between serum threonine and urinary 4-deoxyerythreonic acid which supports the hypothesis that endogenous catabolism of threonine is a source of this metabolite. However, further work is required to understand the relationship between 4-deoxyerythronic acid and metabolic health.

### Habitual dietary intake and the child metabolome

We have confirmed in children a number of known diet-metabolite associations in adults, including meat (which has high creatine content) with urine creatine [99], vegetables and fruits with urine hippurate [100, 101], fruits with proline betaine and *scyllo*-inositol [47, 102]. It is also of note that all 12 metabolites associated with fish intake in the study were serum glycerophosphatidylcholine metabolites; oily fish in the diet alters glycerophospholipid composition and is an important nutrient source for polyunsaturated fatty acids [103, 104]. The extent to which metabolic phenotypes mediate the impact of dietary behaviour on childhood adiposity and cardiovascular indicators will be a focus of our future work. We anticipate that the metabolic phenotyping dataset acquired on the HELIX study population will provide a useful molecular resource to help elucidate the complex interactions between childhood environmental and dietary exposures and adverse health outcomes.

### Complementarity between the serum and urine metabolome

In the HELIX study, matched urine and serum samples across six European cohorts were collected according to well-defined protocols, providing a valuable resource for uncovering metabolic relationships across the two most accessible biological fluid types. Whilst NMR spectroscopy and LC-MS/MS-based metabolic profiling have been widely applied in epidemiological studies [34, 68, 83, 105, 106], our study is one of very few that allows comparison of the effects of pre-analytical, analytical, demographic, dietary and geographic variation between the two biofluid types from the same sample population. It has previously been reported that biological variations are more robustly captured in a blood metabolic profile compared to urine [107]. In our study, we confirm that the combined information from demographics, diet and cohort accounts for greater variance in the LC-MS/MS serum profile compared to NMR urine profile, even if the LC-MS/MS serum profile is more susceptible to analytical batch effects. However, with respect to dietary habits specifically, these are better reflected in the urinary metabolome presumably due to high metabolite turnover, and it has previously been reported in a colon adenoma case-control study (*n* = 253) that more metabolites in urine were uniquely associated with diet than in serum [47]. Our pairwise metabolite correlation analyses also potentially confer information about metabolic pathway activities: urinary acetate with formate and succinate (TCA cycle activity and gut bacterial metabolism); urinary creatine with choline/carnitine (meat diet); 3-indoxylsulfate and *p*-cresol sulphate (both sulphated uremic solutes produced by gut bacteria); urinary dimethylamine with trimethylamine, and trimethylamine oxide (amine derivatives), and urinary and serum valine with leucine (branched-chain amino acid metabolism). Our correlation analysis between metabolite concentrations across the two biological fluid types confirmed that for many compounds, metabolite concentrations between urine and serum are positively correlated and also confirmed metabolic pathway associations with serum threonine and urinary 4-deoxyerythronic acid (threonine catabolism) [74].

### Limitations

Our study had a number of important limitations. Firstly, the sample size from each of the six individual cohorts was relatively small (*n*~200) for observational studies, limiting the statistical power available to uncover novel metabolic associations, particularly when effect sizes were generally small. There were also notable differences in sample characteristics between the cohorts, particularly in age, with the median cohort child age varying from 6 to 11 years old, making it difficult to disentangle cohort level differences from other covariates in our variance decomposition analyses, as those confounders were heavily correlated. Our study also lacks 24-h dietary recall data, and serum samples were collected from non-fasting states. In addition, we acknowledge the inherent limitations in the use of food frequency questionnaire which include the potential for dietary intake misclassifications and that categorising distinct food sources into groups may be imperfect. For example, cocoa could be considered as a vegetable but was classified as sweets in this study. We intend to follow up the metabolite—diet associations identified in this study with detailed food subgroup analyses as part of a future publication.

To make certain the timely completion of an annotated metabolome resource, we have decided to acquire and to process the serum and urine metabolic data using analytical methods which quantify omnipresent metabolites that were typically detected well in this study. Whilst this approach had the advantage of improving the sensitivity and specificity of the quantitation and provide explicit metabolite identification, it limited the number of metabolites that were measured and resulted in only partial coverage of the serum and urine metabolome. Also, the serum metabolic assay only provided partial specificity in the assignment of lipid species as the locations of double bonds or the length of the fatty acid chains remain ambiguous. Supplementing the current study with other complementary metabolomic approaches such as untargeted LC-MS and GC-MS analyses in future would help enhance metabolite coverage and greatly augment the metabolome resource of healthy children available at present.

## Conclusions

We have characterised the major components of the urine and serum metabolome in the HELIX subcohort. Typically but not universally, metabolic associations with age, sex, BMI z-score and dietary habits were common to the six populations studied. Also, a novel metabolic association between threonine catabolism and BMI of children was identified. Inter-metabolite correlation analyses for both urine and serum metabolic phenotypes revealed potential pathway associations, and population-specific variance (demographic, dietary and country of origin) was better captured in the serum than in the urine metabolic profile. This study establishes a reference metabolome resource in multiple European populations for urine and serum from healthy children. This provides a critical foundation for future work to define the utility of metabolic profiles to monitor or predict the impact of environmental and other exposures on human biology and child health.

## Additional files

Additional file 1: Supplementary figures and tables. (PDF 1430 kb)

Additional file 2:  Histograms of serum metabolite distributions. (PDF 111 kb)

Additional file 3: Histograms of urinary metabolite distributions. (PDF 29 kb)

## Abbreviations

BCAA: Branched-chain amino acids
BiB: Born in Bradford, UK
BLD: Below the limit of detection
BMI: Body mass index
CI: Confidence interval
EDEN: Study of determinants of pre- and postnatal developmental, France
HELIX: The Human Early-Life Exposome project
HPLC: High-performance liquid chromatography
INMA: Infancia y Medio Ambiente, Environment and Childhood, Spain
IQR: Interquartile range
KANC: Kaunas Cohort, Lithuania
LC-MS/MS: Liquid chromatography tandem mass spectrometry
LOD: Limit of detection
LPC: Lysophosphatidylcholine
MoBa: The Norwegian Mother and Child Cohort Study, Norway
NMR: Nuclear magnetic resonance
PC: Phosphatidylcholine
PCA: Principal component analysis
QRILC: Quantile regression approach for left-censored missing
Rhea: Mother-Child Cohort in Crete, Greece
SM: Sphingomyelin
