# Supplementary material for: Determinants of the urinary and serum metabolome in children from six European populations
Source: BMC Med. 2018 Nov 8;16:202. doi: 10.1186/s12916-018-1190-8 (PMC6223046; doi:10.1186/s12916-018-1190-8)
Supplement: Supplementary file 1 — Supplementary figures and tables. (PDF 1430 kb) [file 12916_2018_1190_MOESM1_ESM.pdf]

Supplementary Figures and Tables

Determinants of the urinary and serum metabolome in children from six European populations

Chung-Ho E. Lau[1 † *], Alexandros P. Siskos[1, 2 †], Léa Maitre[3,4,5], Oliver Robinson[6], Toby J. Athersuch[1,6], Elizabeth J. Want[1], Jose Urquiza[3,4,5], Maribel Casas[3,4,5], Marina Vafeiadi[7], Theano Roumeliotaki[7], Rosemary R.C. McEachan[9], Rafaq Azad[9], Line S. Haug[10], Helle M. Meltzer[10], Sandra Andrusaityte[11], Inga Petraviciene[11], Regina Grazuleviciene[11], Cathrine Thomsen[10], John Wright[9], Remy Slama[8], Leda Chatzi[12], Martine Vrijheid[3,4,5], Hector C. Keun[2#], Muireann Coen[1,13 #*]

† Authors contributed equally to the work

# Joint senior authors

*Corresponding authors: Dr. Chung-Ho E. Lau (esmond.lau06@imperial.ac.uk) and Dr. Muireann Coen (m.coen@imperial.ac.uk)

Supplementary Figure 1 Distribution of postprandial time interval (between last meal and blood sample collection)

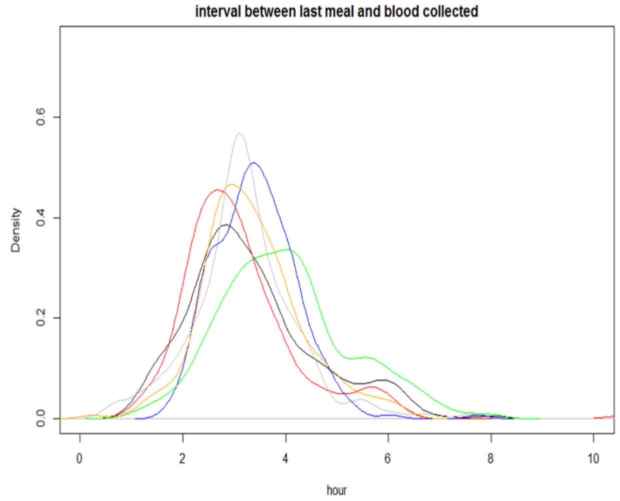

BiB; INMA; EDEN; MoBa; Rhea; KANC

Supplementary Figure 2 Effect of postprandial period on serum metabolite levels

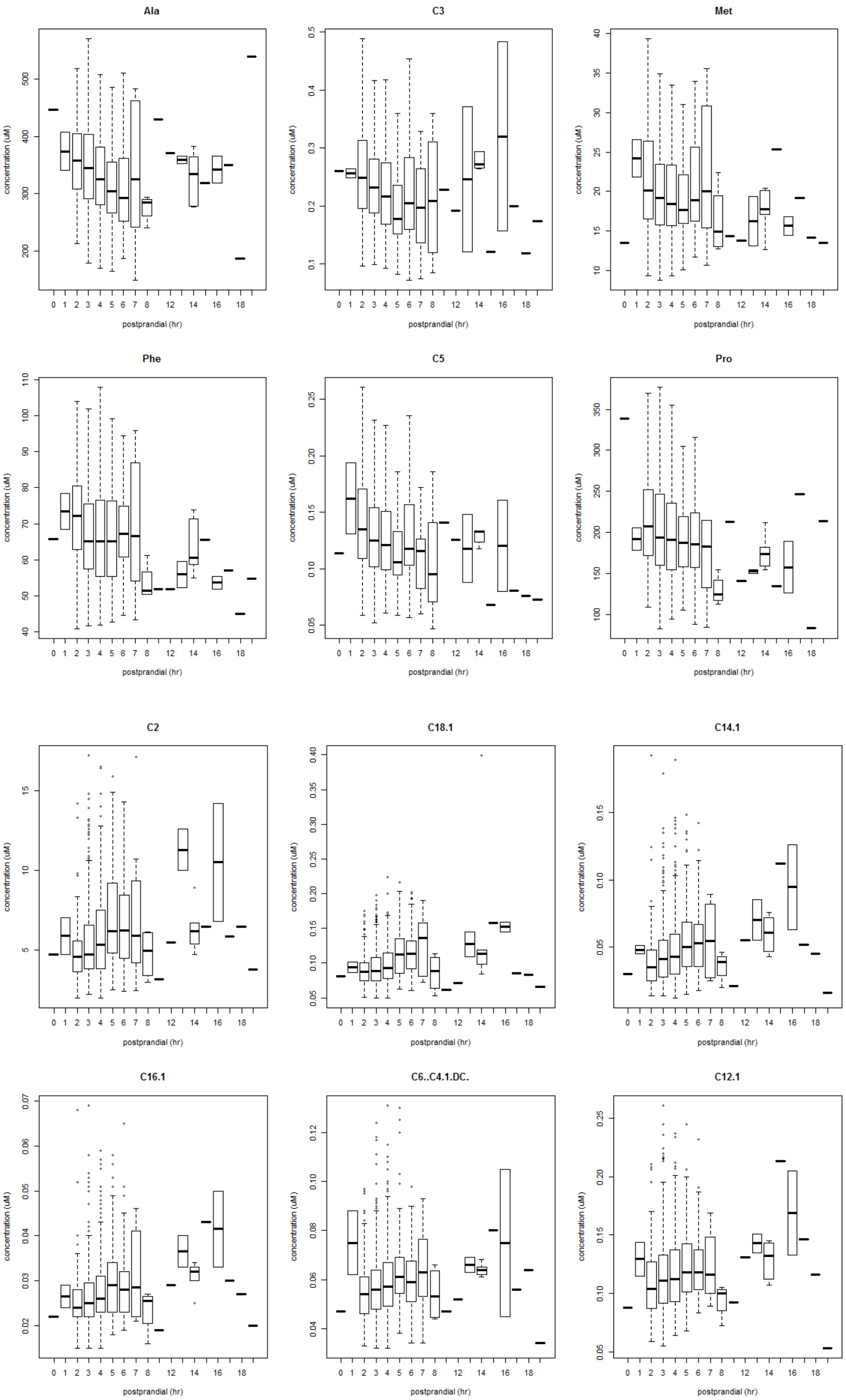

Supplementary Figure 3 Associations between urine creatinine and age by cohort. Coefficients represent SD per year

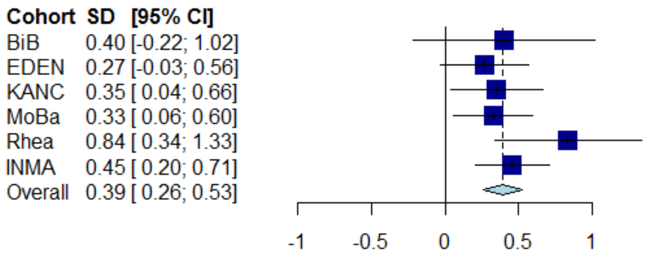

Supplementary Figure 4 Associations between serum creatinine and age by cohort. Coefficients represent SD per year

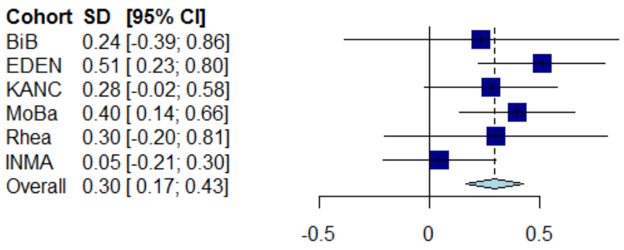

Supplementary Figure 5 Associations of urine 4-deoxyerythronic acid with zBMI by cohort. Coefficients represent SD per BMI z-score

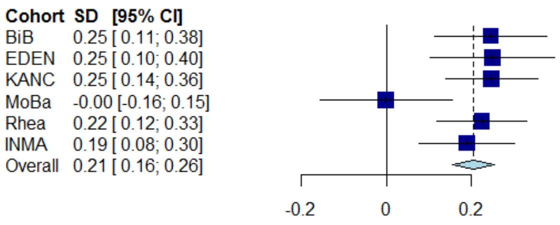

Supplementary Figure 6 Associations of urine valine with zBMI by cohort. Coefficients represent SD per BMI z-score

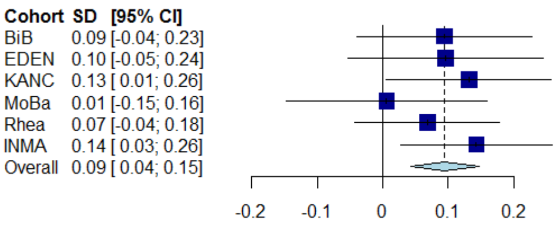

Supplementary Figure 7 Associations of serum glutamate with zBMI by cohort. Coefficients represent SD per BMI z-score

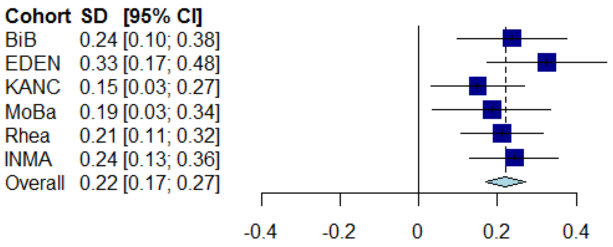

Supplementary Figure 8 Associations of serum carnitine (C0) with zBMI by cohort

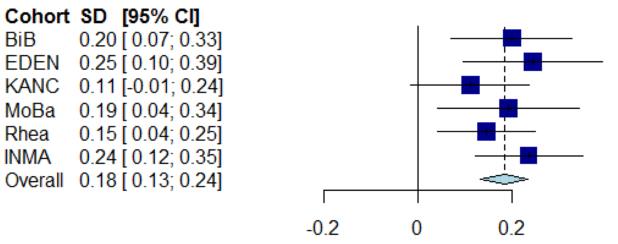

Supplementary Figure 9 PCA analysis - cumulative R^2 (variance explained) of the urine and serum data model. Data were log10 transformed, mean-centred and univariate scaled prior to modelling

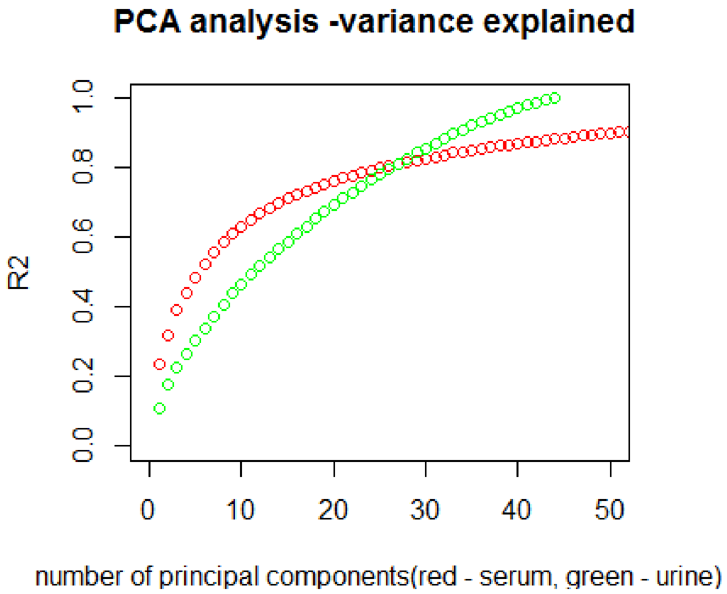

Supplementary Figure 10 Cross urine-serum metabolic correlation based on pairwise correlations across the two bio fluid types.

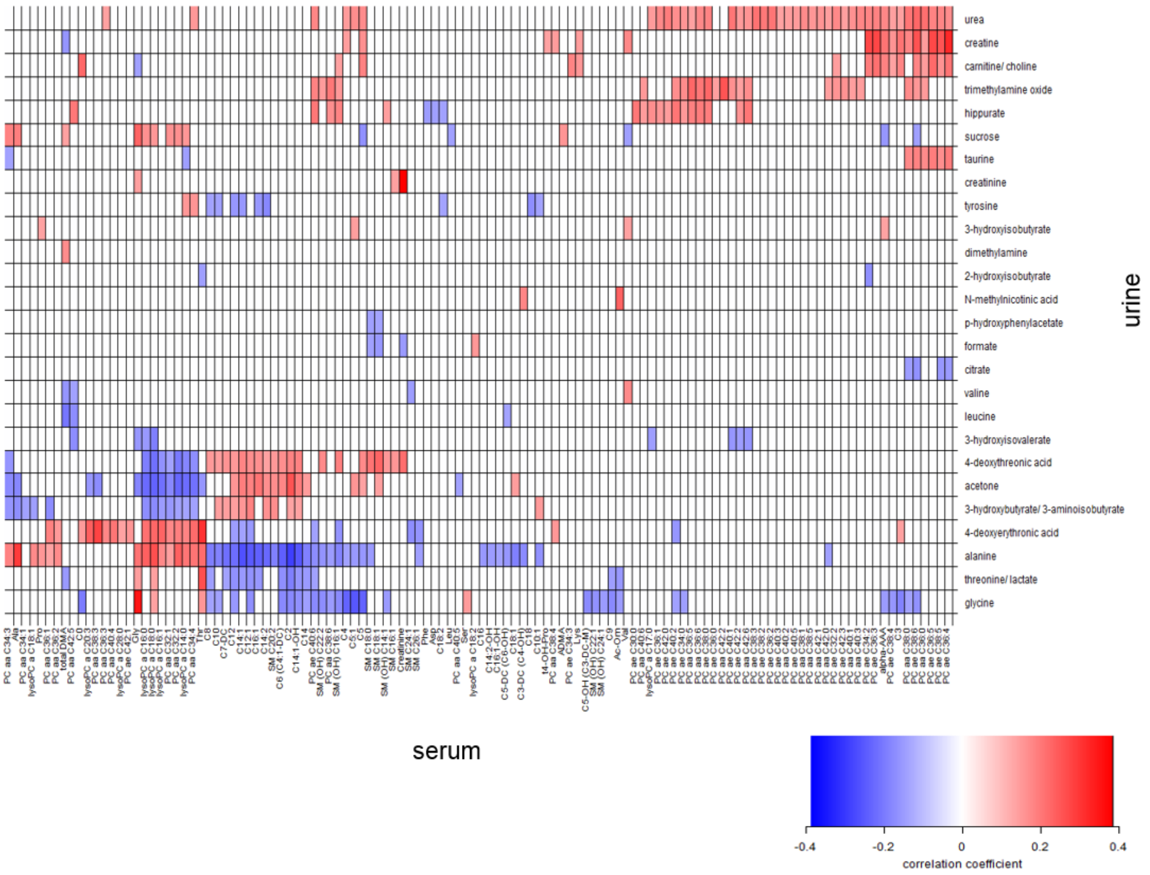

Supplementary Table 1 ^{1}H NMR spectroscopy urinary metabolite annotations and assignments

Metabolite	ChEBI ID	HMDB ID	Compound Class	Resonance (δ / ppm)	Evidence for assignment
leucine	25017	HMDB0000687	Amino acids, peptides, and analogues	0.97	TOCSY
isoleucine	24898	HMDB0000172	Amino acids, peptides, and analogues	1.02	TOCSY
valine	27266	HMDB0000883	Amino acids, peptides, and analogues	1.04	TOCSY
3-hydroxyisobutyrate	11805	HMDB0000023	Beta hydroxy acids and derivatives	1.08	HSQC
4-deoxyerythronic acid	86347	HMDB0000498	Sugar acids and derivatives	1.11	TOCSY, Spike-in
4-deoxythreonic acid	86391	HMDB0002453	Sugar acids and derivatives	1.24	TOCSY, Spike-in
threonine / lactate	26986/24996	HMDB0000167/ HMDB0000190	Amino acids, peptides, and analogues/ Alpha hydroxy acids and derivatives	1.33	STOCSY, HSQC
2-hydroxyisobutyrate	19641	HMDB0000729	Alpha hydroxy acids and derivatives	1.36	HSQC
alanine	16977	HMDB0000161	Amino acids, peptides, and analogues	1.48	STOCSY
lysine	25094	HMDB0000182	Amino acids, peptides, and analogues	1.74	STOCSY
acetate	30089	HMDB0000042	Carboxylic acids	1.93	HSQC
p-cresol sulfate	82914	HMDB0011635	Arylsulfates	2.35	HSQC, STOCSY
succinate	26806	HMDB0000254	Dicarboxylic acids and derivatives	2.41	HSQC
glutamine	28300	HMDB0000641	Amino acids, peptides, and analogues	2.46	STOCSY
citrate	30769	HMDB0000094	Tricarboxylic acids and derivatives	2.54	STOCSY
dimethylamine	17170	HMDB0000087	amines	2.73	STOCSY
trimethylamine	18139	HMDB0000906	Amines	2.87	HSQC
carnitine / choline	17126/15354	HMDB0000062/ HMDB0000097	Carnitines/ Cholines	3.23	HSQC
trimethylamine *N*-oxide	15724	HMDB0000925	Aminoxides	3.28	HSQC
scyllo-inositol	10642	HMDB0006088	Alcohols and polyols	3.37	HSQC
taurine	15891	HMDB0000251	Organosulfonic acids	3.43	STOCSY
glycine	15428	HMDB0000123	Amino acids, peptides, and analogues	3.58	HSQC
creatine	16919	HMDB0000064	Amino acids, peptides, and analogues	3.94	HSQC
creatinine	16737	HMDB0000562	Amino acids, peptides, and analogues	4.06	HSQC
hippurate	132966	HMDB0000714	Benzoic acids and derivatives	7.56	STOCSY
formate	15740	HMDB0000142	Carboxylic acids	8.47	HSQC
pantothenic acid	7916	HMDB0000210	Polyols	0.94	Spike-in
3-hydroxybutyrate / 3-aminoisobutyrate	37054/49096	HMDB0000357/HMDB0003911	Beta hydroxy acids and derivatives/ Amino acids, peptides, and analogues	1.19	TOCSY, Spike-in
3-hydroxyisovalerate	82957	HMDB0000754	Hydroxy fatty acids	1.27	STOCSY
N-acetyl neuraminic acid	45744	HMDB0000230	Carbohydrates and carbohydrate conjugates	2.06	Spike-in
acetone	15347	HMDB0001659	Ketones	2.24	HSQC
5-oxoproline	16010	HMDB0000267	Amino acids, peptides, and analogues	2.39	TOCSY, HSQC, Spike-in
3-aminoisobutyrate	49096	HMDB0003911	Amino acids, peptides, and analogues	2.62	TOCSY
proline betaine	35280	HMDB0004827	Amino acids, peptides, and analogues	3.30	HSQC
glucose	17234	HMDB0000122	Carbohydrates and carbohydrate conjugates	5.25	TOCSY, HSQC, STOCSY

Metabolite	ChEBI ID	HMDB ID	Compound Class	Resonance (δ / ppm)	Evidence for assignment
sucrose	17992	HMDB0000258	Carbohydrates and carbohydrate conjugates	5.42	TOCSY, HSQC, STOCSY
urea	16199	HMDB0000294	Organic carbonic acids and derivatives	5.81	tentative
N-methyl-2-pyridone-5-carboxamide	27410	HMDB0004193	Nicotinamides	6.67	TOCSY, STOCSY, Spike-in
p-hydroxyphenylacetate	18101	HMDB0000020	Phenols	6.88	STOCSY
tyrosine	18186	HMDB0000158	Amino acids, peptides, and analogues	6.91	TOCSY
3-Indoxylsulfate	43355	HMDB0000682	Arylsulfates	7.52	TOCSY, HSQC, STOCSY
N-methylpicolinic acid	69061	Not Available	Alkaloids and derivatives	8.72	TOCSY
N-methylnicotinic acid	50521	HMDB0000875	Alkaloids and derivatives	9.12	STOCSY
N_1-methyl-nicotinamide	16797	HMDB0000699	Nicotinamides	9.28	STOCSY

NB. Assignments were ambiguous for 3 of the metabolite signals: Threonine / Lactate (δ 1.33) – resonance peak contain signal contributions from both threonine and lactate. Carnitine / Choline (δ 3.23) - resonance peak contain signal contributions from both carnitine and choline. 3-hydroxybutyrate / 3-aminoisobutyrate (δ 1.19) - resonance peak contain signal contributions from both 3-hydroxybutyrate and 3-aminoisobutyrate. HMDB identifier for *N*-methylpicolinic acid cannot be located on the Human Metabolome Database website.

Supplementary Table 2 Non-detection rates of urinary metabolites and coefficient of variation (CV)

Signals	Non-detected (%)	Coefficient of variation (%)
creatinine	0%	7%
leucine	0%	7%
isoleucine	1%	14%
valine	0%	10%
3-hydroxyisobutyrate	0%	7%
4-deoxyerythronic acid	0%	8%
4-deoxythreonic acid	0%	6%
threonine/ lactate	0%	4%
2-hydroxyisobutyrate	0%	6%
alanine	0%	6%
lysine	0%	7%
acetate	0%	22%
p-cresol sulfate	0%	8%
succinate	7%	12%
glutamine	1%	5%
citrate	0%	7%
dimethylamine	1%	14%
trimethylamine	0%	7%
carnitine / choline	1%	12%
trimethylamine *N*-oxide	2%	6%
scyllo-inositol	4%	17%
taurine	1%	5%
glycine	0%	9%
creatine	0%	7%
hippurate	0%	6%
formate	0%	9%
pantothenic acid	0%	7%
3-hydroxybutyrate / 3-aminoisobutyrate	3%	10%
3-hydroxyisovalerate	0%	6%
N-acetyl neuraminic acid	1%	13%
acetone	1%	28%
5-oxoproline	1%	12%
3-aminoisobutyrate	2%	10%
proline betaine	8%	20%
glucose	0%	11%
sucrose	1%	6%
urea	0%	8%
N-methyl-2-pyridone-5-carboxamide	6%	12%
p-hydroxyphenylacetate	0%	7%
tyrosine	0%	10%
3-Indoxylsulfate	2%	15%
N-methylpicolinic acid	46%	27%
N-methylnicotinic acid	0%	13%
N_1-methyl nicotinamide	1%	20%

Supplementary Table 3 Coefficient of variation (CV) and percentage of measurments below the limit of detection (%BLD) per metabolite

Metabolite short name	Common name	ChEBI ID	Class	Assay	CV %	% BLD
Ala	L-Alanine	15570 / 16977	aminoacids	LC	7.5	0
Arg	L-Arginine	29016 / 15816 / 16467	aminoacids	LC	8.3	0
Asn	L-Asparagine	22653 / 17196 / 28159	aminoacids	LC	8.1	0
Asp	L-Aspartic acid	22660 / 17053 / 17364	aminoacids	LC	14.9	0
Cit	L-Citrulline	18211 / 16349 / 49007	aminoacids	LC	10.2	0
Gln	L-Glutamine	28300 / 17061 / 18050	aminoacids	LC	7.7	0
Glu	L-Glutamic acid	18237 / 15966 / 16015	aminoacids	LC	9	0
Gly	Glycine	15428	aminoacids	LC	7	0
His	L-Histidine	27570 / 15971 / 27947	aminoacids	LC	7.8	0
Ile	L-Isoleucine	38264 / 22359 / 24898 / 17191 / 43433	aminoacids	LC	7.6	0
Leu	L-Leucine	25017 / 15603 / 28225	aminoacids	LC	7.8	0
Lys	L-Lysine	25094 / 16855 / 18019	aminoacids	LC	9	0
Met	L-Methionine	16811 / 16643 / 16867	aminoacids	LC	12.2	0
Orn	L-Ornithine	18257 / 15729 / 16176	aminoacids	LC	8.6	0
Phe	L-Phenylalanine	28044 / 16998 / 17295	aminoacids	LC	8.4	0
Pro	L-Proline	26271 / 16313 / 17203	aminoacids	LC	7.9	0
Ser	L-Serine	17822 / 16523 / 17115	aminoacids	LC	8	0
Thr	L-Threonine	16857 / 38263 / 26986 / 28718 / 38262	aminoacids	LC	8	0
Trp	L-Tryptophan	27897 / 16296 / 16828	aminoacids	LC	8.5	0
Tyr	L-Tyrosine	18186 / 17895 / 28479	aminoacids	LC	8.4	0
Val	L-Valine	27266 / 16414 / 27477	aminoacids	LC	7.3	0
Ac-Orn	N-Acetylornithine	16543 / 44673	biogenic amines	LC	14.5	19.2
ADMA	Asymmetric dimethylarginine	17929	biogenic amines	LC	11.8	0
alpha-AAA	alpha-Aminoadipic acid	37024 / 37023 / 37025	biogenic amines	LC	21.8	5.6
Creatinine	Creatinine	16737	biogenic amines	LC	7.5	0
Histamine (#)	Histamine	18295	biogenic amines	LC	56.5	100
Kynurenine	Kynurenine	28683 / 16946 / 86262	biogenic amines	LC	9.6	0
Met-SO	Methionine Sulfoxide	17016 / 49031 / 49032 / 49033 / 49034	biogenic amines	LC	16.4	4.8
Putrescine	Putrescine	17148	biogenic amines	LC	44.8	5.1
SDMA	Symmetric dimethylarginine	25682	biogenic amines	LC	35.9	5.4
Serotonin	Serotonin	28790	biogenic amines	LC	24	0
Spermidine	Spermidine	16610	biogenic amines	LC	17.1	66.5
Spermine	Spermine	15746	biogenic amines	LC	36.6	3
t4-OH-Pro	trans-4-Hydroxyproline	18095 / 86499 / 20392	biogenic amines	LC	10	0
Taurine	Taurine	15891	biogenic amines	LC	7.6	0
total DMA	Total dimethylarginine	86468 / 17929 / 25682	biogenic amines	LC	14.1	0
c4-OH-Pro (#)	cis-4-Hydroxyproline	18095 / 20392	biogenic amines	LC	NA	99.9
Carnosine (#)	Carnosine	15727	biogenic amines	LC	274.9	97.9
DOPA (#)	DOPA	49168 / 49169 / 15765	biogenic amines	LC	NA	99.9
Dopamine (#)	Dopamine	18243	biogenic amines	LC	306.2	99.0
Nitro-Tyr (#)	Nitrotyrosine	86267	biogenic amines	LC	617.2	100.0
PEA (#)	Phenylethylamine	50048	biogenic amines	LC	612.3	97.7
C0	L-Carnitine	17126 / 11060 / 16347	acylcarnitines	FIA	7.6	0

Metabolite short name	Common name	ChEBI ID	Class	Assay	CV %	% BLD
C10	Decanoylcarnitine	68830 / 28717	acylcarnitines	FIA	8.6	16.9
C10:1	Decenoylcarnitine	86063 / 73048	acylcarnitines	FIA	10.3	68.6
C10:2	Decadienylcarnitine	Not Available	acylcarnitines	FIA	8.8	99.8
C12	Dodecanoylcarnitine	73054 / 77086	acylcarnitines	FIA	8.1	82.5
C12-DC	Dodecanedioylcarnitine	74121	acylcarnitines	FIA	9.4	100
C12:1	Dodecenoylcarnitine	89717 / 86065 / 73053	acylcarnitines	FIA	10.1	71.3
C14	Tetradecanoylcarnitine	73061 / 84634	acylcarnitines	FIA	13.8	99.5
C14:1	Tetradecenoylcarnitine	86066 / 83049 / 85449	acylcarnitines	FIA	11.9	1.2
C14:1-OH	Hydroxytetradecenoylcarnitine	86067 / 73062	acylcarnitines	FIA	13.4	98.8
C14:2	Tetradecadienylcarnitine	86069 / 73059 / 85451	acylcarnitines	FIA	12.1	30.6
C14:2-OH	Hydroxytetradecadienylcarnitine	86070 / 73108 / 85453	acylcarnitines	FIA	19.2	100
C16	Hexadecanoylcarnitine	73067 / 17490	acylcarnitines	FIA	10.4	0.1
C16-OH	Hydroxyhexadecanoylcarnitine	73070 / 86030	acylcarnitines	FIA	24	100
C16:1	Hexadecenoylcarnitine	86032 / 86031	acylcarnitines	FIA	8.9	93.3
C16:1-OH	Hydroxyhexadecenoylcarnitine	86033 / 85456	acylcarnitines	FIA	14.4	98.5
C16:2	Hexadecadienylcarnitine	86035 / 73065	acylcarnitines	FIA	22.5	99.4
C16:2-OH	Hydroxyhexadecadienylcarnitine	73109 / 86036	acylcarnitines	FIA	11.9	100
C18	Octadecanoylcarnitine	84644 / 73074	acylcarnitines	FIA	10.1	10.3
C18:1	Octadecenoylcarnitine	86475 / 72689 / 86038	acylcarnitines	FIA	9	30.9
C18:1-OH (#)	Hydroxyoctadecenoylcarnitine	86039 / 73076	acylcarnitines	FIA	56.7	100
C18:2	Octadecadienylcarnitine	86480 / 72715 / 85463	acylcarnitines	FIA	10.6	1.5
C2	Acetylcarnitine	73024 / 57589 / 86045	acylcarnitines	FIA	7.4	0
C3	Propionylcarnitine	28867 / 53210	acylcarnitines	FIA	15.9	0
C3-DC (C4-OH)	Hydroxybutyrylcarnitine	86047 / 73028 / 72995 / 84842	acylcarnitines	FIA	17.4	67.2
C3-OH	Hydroxypropionylcarnitine	86072	acylcarnitines	FIA	29.4	99.9
C3:1 (#)	Propenoylcarnitine	86073	acylcarnitines	FIA	65.5	100
C4	Butyrylcarnitine	7676 / 21949 / 73017 / 84838 / 84838	acylcarnitines	FIA	16.5	0
C4:1	Butenylcarnitine	88785	acylcarnitines	FIA	15.4	99.7
C6 (C4:1-DC)	Fumaryl-L-carnitine / Hexanoyl-L-carnitine	70749 / 84834 / 87264	acylcarnitines	FIA	10.1	79.8
C5	Valeryl-L-carnitine	86492 / 73025 / 70819 / 73026 / 86050 / 86049	acylcarnitines	FIA	9.4	1.4
C5-M-DC	Methylglutarylcarnitine	86486 / 70857	acylcarnitines	FIA	17.3	99.9
C5-OH (C3-DC-M)	Hydroxyvalerylcarnitine (Methylmalonylcarnitine)	86467 / 73031	acylcarnitines	FIA	8.7	100
C5:1	Tiglylcarnitine	71179 / 85520	acylcarnitines	FIA	17.2	100
C5:1-DC	Glutaconylcarnitine	86081 / 85518	acylcarnitines	FIA	21.7	99.9
C5-DC (C6-OH)	Glutaryl-L-carnitine / Hydroxyhexanoyl-L-carnitine	73040 / 82952 / 88772	acylcarnitines	FIA	17.7	98.5
C6:1	Hexenoylcarnitine	88762 / 86082	acylcarnitines	FIA	14.1	99.9
C7-DC	Pimelylcarnitine	86084 / 85525	acylcarnitines	FIA	13.5	99.7
C8	Octanoylcarnitine	73039 / 18102 / 86051	acylcarnitines	FIA	10.7	82.7
C9	Nonaylcarnitine	86493 / 70997 / 85527 / 86054	acylcarnitines	FIA	14.2	99.9
lysoPC a C14:0		64483 / 64489	glycerophospholipids	FIA	8.1	99.9
lysoPC a C16:0		64563 / 72998 / 76078	glycerophospholipids	FIA	11.1	0
lysoPC a C16:1		64560 / 73851 / 84752	glycerophospholipids	FIA	11.1	0

Metabolite short name	Common name	ChEBI ID	Class	Assay	CV %	% BLD
lysoPC a C17:0		72737 / 74340	glycerophospholipids	FIA	10.8	0
lysoPC a C18:0		64561 / 73858 / 76076	glycerophospholipids	FIA	11.4	0
lysoPC a C18:1		64566 / 72589 / 76083 / 86255	glycerophospholipids	FIA	11	0
lysoPC a C18:2		64549 / 28733 / 76084	glycerophospholipids	FIA	11	0
lysoPC a C20:3		64481 / 64488 / 74343 / 86256	glycerophospholipids	FIA	10.8	0
lysoPC a C20:4		64568 / 74344 / 76079 / 86259	glycerophospholipids	FIA	10.7	0
lysoPC a C24:0		74470 / 86260	glycerophospholipids	FIA	35.8	0
lysoPC a C26:0		74472	glycerophospholipids	FIA	56	0
lysoPC a C26:1		74473	glycerophospholipids	FIA	58	0.6
lysoPC a C28:0		85561	glycerophospholipids	FIA	37.5	0.3
lysoPC a C28:1		85562	glycerophospholipids	FIA	43	0
PC aa C24:0		85564 / 65211 / 75302 / 86276 / 86278 / 86280 / 86281 / 86282	glycerophospholipids	FIA	51	1
PC aa C26:0 (#)		Not Available	glycerophospholipids	FIA	42.5	60.1
PC aa C28:1		65293 / 86410	glycerophospholipids	FIA	10.9	0
PC aa C30:0		65303 / 75062 / 75304 / 86088	glycerophospholipids	FIA	8.2	0
PC aa C30:2		65301 / 86415	glycerophospholipids	FIA	56.4	14.6
PC aa C32:0		66850 / 72999 / 86089 / 86090	glycerophospholipids	FIA	12.7	0
PC aa C32:1		66849 / 73793 / 74000 / 84808 / 86092	glycerophospholipids	FIA	13.3	0
PC aa C32:2		66848 / 83717	glycerophospholipids	FIA	14.9	0
PC aa C32:3		66847 / 86095	glycerophospholipids	FIA	14.7	0
PC aa C34:1		64517 / 73001 / 74667 / 84570 / 85037 / 86097	glycerophospholipids	FIA	12.2	0
PC aa C34:2		64516 / 73002 / 84785 / 84809 / 84810 / 84811 / 86099 / 86100	glycerophospholipids	FIA	12.7	0
PC aa C34:3		64424 / 84567 / 84786 / 84789	glycerophospholipids	FIA	13.1	0
PC aa C34:4		64423 / 86101 / 86102	glycerophospholipids	FIA	13.7	0
PC aa C36:0		66858 / 75294 / 83718 / 86103 / 86106 / 86107	glycerophospholipids	FIA	13.4	0
PC aa C36:1		66857 / 76073 / 84575 / 84818	glycerophospholipids	FIA	11.1	0
PC aa C36:2		64433 / 74669 / 84574 / 84819 / 84820 / 84821 / 84822 / 84836 / 86112	glycerophospholipids	FIA	11.6	0
PC aa C36:3		64523 / 74670 / 84831 / 86117 / 86119 / 86121	glycerophospholipids	FIA	11.9	0
PC aa C36:4		64520 / 73003 / 77694 / 84794 / 84815 / 86123 / 86127 / 86129 / 86130 / 86133 / 86134	glycerophospholipids	FIA	11.8	0
PC aa C36:5		64504 / 64505 / 84816 / 86137	glycerophospholipids	FIA	12.2	0
PC aa C36:6		66856 / 86161 / 86162	glycerophospholipids	FIA	14.6	0
PC aa C38:0		66861 / 86163 / 86164 / 86166 / 86167 / 86168 / 86169	glycerophospholipids	FIA	10.7	0
PC aa C38:1		66860 / 86170 / 86171 / 86173 / 86174 / 86175	glycerophospholipids	FIA	32.2	0
PC aa C38:3		64446 / 74479 / 86176 / 86177	glycerophospholipids	FIA	11.1	0
PC aa C38:4		64526 / 74965 / 84573 / 84823 / 86178	glycerophospholipids	FIA	11.2	0
PC aa C38:5		64525 / 74671 / 84795 / 84796 / 86181	glycerophospholipids	FIA	11	0
PC aa C38:6		64519 / 74963 / 84563 / 84797 / 86182 / 86183 / 86184 / 86186 / 86187	glycerophospholipids	FIA	11.3	0
PC aa C40:1		66865 / 86188 / 86189 / 86190 / 86196 / 86197	glycerophospholipids	FIA	12	64.9
PC aa C40:2		66864 / 86198 / 86199	glycerophospholipids	FIA	13.9	0
PC aa C40:3		66863 / 86416	glycerophospholipids	FIA	13.8	0
PC aa C40:4		66862 / 74970 / 84565 / 86200	glycerophospholipids	FIA	10.2	0
PC aa C40:5		64524 / 73865 / 84151 / 86201 / 86203	glycerophospholipids	FIA	11	0
PC aa C40:6		64431 / 73868 / 84829 / 84830 / 86205	glycerophospholipids	FIA	10.4	0
PC aa C42:0		66971 / 86208 / 86210 / 86211 / 86212	glycerophospholipids	FIA	10.7	0

Metabolite short name	Common name	ChEBI ID	Class	Assay	CV %	% BLD
PC aa C42:1		66970 / 86213 / 86215 / 86216 / 86217 / 86219	glycerophospholipids	FIA	13.9	0
PC aa C42:2		66969 / 86418	glycerophospholipids	FIA	14.7	0
PC aa C42:4		66967 / 86422	glycerophospholipids	FIA	13.7	0
PC aa C42:5		66966 / 86220	glycerophospholipids	FIA	10.7	0
PC aa C42:6		66965 / 86221	glycerophospholipids	FIA	11	2.4
PC ae C30:0		85565	glycerophospholipids	FIA	15.9	0.1
PC ae C30:1		85566 / 86225	glycerophospholipids	FIA	53.5	0
PC ae C30:2		85567 / 86423	glycerophospholipids	FIA	32.9	0
PC ae C32:1		72347 / 86424	glycerophospholipids	FIA	14.2	0
PC ae C32:2		85568 / 86425	glycerophospholipids	FIA	16.3	0
PC ae C34:0		67077 / 86229	glycerophospholipids	FIA	12.8	0
PC ae C34:1		67076 / 84825	glycerophospholipids	FIA	12.9	0
PC ae C34:2		64544 / 86231	glycerophospholipids	FIA	13.3	0
PC ae C34:3		64541 / 86427	glycerophospholipids	FIA	13.2	0
PC ae C36:0		67067 / 86239 / 86240	glycerophospholipids	FIA	12.7	0
PC ae C36:1		67066 / 86432	glycerophospholipids	FIA	11.1	0
PC ae C36:2		67065 / 86504	glycerophospholipids	FIA	11.7	0
PC ae C36:3		64537 / 86436	glycerophospholipids	FIA	12.4	0
PC ae C36:4		67064 / 55430	glycerophospholipids	FIA	12.2	0
PC ae C36:5		64540 / 86437	glycerophospholipids	FIA	12.6	0
PC ae C38:0		67071 / 74482 / 86243	glycerophospholipids	FIA	11.5	0
PC ae C38:1		67070 / 86438	glycerophospholipids	FIA	29.3	0.5
PC ae C38:2		67069 / 86439	glycerophospholipids	FIA	11.9	0
PC ae C38:3		67068 / 86442	glycerophospholipids	FIA	10.7	0
PC ae C38:4		64480 / 86245	glycerophospholipids	FIA	12.1	0
PC ae C38:5		64445	glycerophospholipids	FIA	11.8	0
PC ae C38:6		64536 / 86248	glycerophospholipids	FIA	11.6	0
PC ae C40:1		85574 / 86443	glycerophospholipids	FIA	16.9	0
PC ae C40:2		72348 / 86444	glycerophospholipids	FIA	10.5	0
PC ae C40:3		85570 / 86445	glycerophospholipids	FIA	11.2	0
PC ae C40:4		85571 / 86447	glycerophospholipids	FIA	9.9	0
PC ae C40:5		85573 / 86448	glycerophospholipids	FIA	11.7	0
PC ae C40:6		64533 / 86252	glycerophospholipids	FIA	11.6	0
PC ae C42:0		86253 / 85582	glycerophospholipids	FIA	10.6	99.9
PC ae C42:1		85583 / 86449	glycerophospholipids	FIA	25	10.3
PC ae C42:2		85584 / 86450	glycerophospholipids	FIA	16	0
PC ae C42:3		85585 / 86451	glycerophospholipids	FIA	15	0
PC ae C42:4		85588 / 86452	glycerophospholipids	FIA	10.3	0
PC ae C42:5		85590	glycerophospholipids	FIA	9	0
PC ae C44:3		67085 / 86454	glycerophospholipids	FIA	28.3	0
PC ae C44:4		67084 / 86456	glycerophospholipids	FIA	13.7	0
PC ae C44:5		67083	glycerophospholipids	FIA	11.1	0
PC ae C44:6		67082	glycerophospholipids	FIA	10.2	0
SM (OH) C14:1		85597 / 90011	sphingolipids	FIA	9.1	0
SM (OH) C16:1		85626 / 90007	sphingolipids	FIA	10	0

Metabolite short name	Common name	ChEBI ID	Class	Assay	CV %	% BLD
SM (OH) C22:1		85599 / 90010	sphingolipids	FIA	15.4	0
SM (OH) C22:2		85600 / 90003	sphingolipids	FIA	15.6	0
SM (OH) C24:1		85601 / 90006	sphingolipids	FIA	16.2	0
SM C16:0		78646	sphingolipids	FIA	9.1	0
SM C16:1		85592 / 64487	sphingolipids	FIA	9.3	0
SM C18:0		83358	sphingolipids	FIA	10.9	0
SM C18:1		85593 / 84487 / 86086	sphingolipids	FIA	10.3	0
SM C20:2		85595	sphingolipids	FIA	13.9	0
SM C22:3 (#)		Not available	sphingolipids	FIA	62.9	35
SM C24:0		83360	sphingolipids	FIA	15.3	0
SM C24:1		83172 / 74535	sphingolipids	FIA	16.4	0
SM C26:0		83361	sphingolipids	FIA	27.4	0
SM C26:1		83362 / 84476	sphingolipids	FIA	27.1	0
H1	Hexose (Sugar)	4167 / 17234 / 17925 / 28061 / 4208 / 15903 / 28645 / 10295 / 47693 / 27667 / 37704 / 4139 / 18133 / 1416 / 17011 / 24973 / 33917 / 33921 / 33984 / 4194 / 80423 / 88378 / 88746 / 88747	sugars	FIA	7.2	0

NB. LC-MS/MS metabolite exclusion was based on a variable meeting two conditions 1) CV of over 30%; and 2) over 30% of the data are below LOD. 11 out of the 188 serum metabolites detected were excluded as a result.# represents metabolites which were excluded from subsequent data analyses. CV for c4-OH-Pro and DOPA are not available as peaks cannot be detected and/ or integrated reproducibly with the quality control samples. DOPA and c4-OH-Pro are counted as part of the 11 exlcuded metabolites.

Supplementary Table 4 Summary of urinary metabolite concentrations across the 6 cohorts

**µmol/mmol of creatinine (median with IQR)**	**BiB** (n = 199)	**EDEN** (n = 157)	**INMA** (n = 207)	**KANC** (n = 201)	**MoBa** (n = 229)	**Rhea** (n = 199)
creatinine (umol/L)	6120 (4760 - 7920)	7180 (6020 - 9060)	6080 (4630 - 7810)	5610 (4180 - 7100)	5630 (4370 - 6800)	5420 (4180 - 7180)
leucine	2.61 (2.1 - 3.26)	2.59 (2.14 - 3.06)	3.11 (2.39 - 3.86)	2.86 (2.33 - 3.42)	2.83 (2.32 - 3.45)	3.2 (2.56 - 3.92)
isoleucine	1.78 (1.26 - 2.64)	1.49 (1.13 - 1.89)	1.68 (1.35 - 2.02)	1.96 (1.4 - 2.64)	1.87 (1.25 - 2.51)	1.69 (1.32 - 2.08)
valine	3.74 (3.2 - 4.65)	3.54 (2.97 - 4.16)	4.13 (3.34 - 4.98)	3.96 (3.42 - 4.81)	3.84 (3.27 - 4.79)	4.46 (3.66 - 5.47)
3-hydroxyisobutyrate	11.9 (9.73 - 15.9)	12.3 (10 - 15.1)	13.7 (10.7 - 18.2)	12.6 (9.82 - 16.7)	12.7 (9.95 - 16.3)	14.3 (10.8 - 19.1)
4-deoxyerythronic acid	10.1 (8.33 - 12.7)	10.4 (8.78 - 13)	12 (9.45 - 14.8)	9.87 (7.67 - 12.2)	9.33 (7.47 - 11.2)	11.8 (9.33 - 15.1)
4-deoxythreonic acid	16.4 (12.9 - 20.1)	19.1 (15.5 - 25.2)	18.5 (15.5 - 22.4)	18.4 (14.5 - 23.6)	17.7 (13.9 - 24.9)	19.1 (14.7 - 24.4)
2-hydroxyisobutyrate	6.29 (5.14 - 7.57)	6.07 (5.15 - 7.07)	6.08 (5.09 - 7.17)	6.94 (5.79 - 8.08)	6.18 (5.22 - 7.62)	6.72 (5.55 - 8.32)
alanine	49.9 (39 - 65.4)	40.7 (33.1 - 56.3)	50.9 (33.8 - 64.2)	47.3 (33.8 - 60.9)	44.3 (34.1 - 56.7)	49.8 (37.5 - 72.3)
lysine	33.1 (25.8 - 44)	27.7 (22.3 - 39.6)	36.3 (26.1 - 49.9)	31.6 (25.5 - 42.8)	30.6 (23.6 - 41.6)	36.8 (27.4 - 51.9)
acetate	9.17 (6.4 - 11.6)	6.51 (5.26 - 8.59)	7.39 (5.67 - 9.73)	8.19 (6.22 - 10.4)	7.84 (5.91 - 10.7)	8.25 (6.8 - 10.8)
p-cresol sulfate	60.2 (30.8 - 89.7)	41.5 (27.4 - 62.5)	46.8 (29.1 - 72.5)	50.9 (29.5 - 82.2)	46.9 (24.7 - 68.8)	44.5 (24.1 - 75.2)
succinate	17.3 (11.3 - 27.7)	12.3 (7.44 - 16.9)	15 (10 - 23)	20.3 (13.9 - 28.3)	18 (12.3 - 25.7)	19.1 (13 - 27.1)
glutamine	134 (106 - 166)	125 (95.1 - 158)	134 (106 - 161)	134 (110 - 160)	131 (111 - 163)	144 (113 - 176)
citrate	575 (388 - 808)	505 (349 - 671)	426 (273 - 602)	418 (252 - 606)	451 (325 - 637)	488 (327 - 683)
dimethylamine	60.2 (53.2 - 70.8)	48 (43.9 - 52)	52.3 (47.4 - 60.2)	61.1 (53.7 - 66.6)	54.8 (50 - 64.3)	60.2 (52.5 - 66.1)
trimethylamine	4.32 (3.08 - 6.03)	3.49 (2.51 - 4.54)	4.39 (3.22 - 5.66)	4.71 (3.28 - 6.07)	3.87 (2.96 - 5.4)	4.13 (3.36 - 5.88)
trimethylamine *N*-oxide	55 (36.6 - 94.2)	66.1 (45.2 - 101)	59.7 (40.3 - 110)	55.3 (42.5 - 80.9)	71.8 (51.3 - 117)	54.2 (39.6 - 88.7)
scyllo-inositol	8.8 (6.38 - 12.5)	11.9 (7.78 - 18.4)	8.91 (5.9 - 13.2)	10.4 (7.37 - 14.2)	9.95 (7.15 - 15.3)	11.9 (9.27 - 16.9)
taurine	115 (66.2 - 176)	134 (88.9 - 189)	166 (121 - 234)	114 (71.3 - 181)	131 (76 - 191)	128 (83.8 - 186)
glyine	111 (78.7 - 158)	91.3 (70.6 - 127)	98 (69.8 - 131)	87.1 (62 - 131)	95.1 (71.6 - 125)	95.4 (68.3 - 141)
creatine	191 (91.8 - 341)	136 (44.8 - 269)	298 (171 - 510)	201 (100 - 372)	187 (89.3 - 425)	207 (124 - 354)
hippurate	199 (135 - 331)	194 (148 - 282)	152 (108 - 251)	295 (204 - 480)	322 (228 - 460)	173 (111 - 288)
formate	21.9 (16.1 - 29.8)	14.3 (10.9 - 20.6)	20.1 (14.6 - 26.7)	20.6 (15.6 - 27.6)	19.1 (13.8 - 24.6)	21.7 (16.6 - 31.1)

Supplementary Table 5 Summary of serum metabolite concentrations across the 6 cohorts

µmol/L (median with IQR)	**BiB** (n = 199)	**EDEN** (n = 157)	**KANC** (n = 201)	**MoBa** (n = 229)	**Rhea** (n = 199)	**INMA** (n = 207)
C0	33.9 (28.8 - 38.8)	36.6 (32.2 - 40.8)	35.3 (31 - 39.9)	35.3 (31 - 40.3)	34.2 (30.7 - 40.2)	36.4 (32.2 - 41.1)
C10	0.23 (0.186 - 0.278)	0.177 (0.149 - 0.237)	0.258 (0.212 - 0.299)	0.216 (0.177 - 0.268)	0.221 (0.184 - 0.257)	0.202 (0.168 - 0.242)
C10:1	0.11 (0.0835 - 0.142)	0.08 (0.067 - 0.097)	0.121 (0.098 - 0.148)	0.096 (0.077 - 0.116)	0.1 (0.083 - 0.123)	0.113 (0.088 - 0.142)
C10:2	0.069 (0.062 - 0.078)	0.068 (0.063 - 0.074)	0.063 (0.058 - 0.068)	0.067 (0.061 - 0.074)	0.06 (0.055 - 0.0655)	0.059 (0.056 - 0.065)
C12	0.107 (0.089 - 0.13)	0.086 (0.077 - 0.111)	0.122 (0.103 - 0.148)	0.103 (0.086 - 0.129)	0.109 (0.091 - 0.126)	0.098 (0.083 - 0.119)
C12-DC	0.231 (0.215 - 0.245)	0.233 (0.216 - 0.245)	0.236 (0.219 - 0.248)	0.23 (0.218 - 0.245)	0.232 (0.218 - 0.245)	0.232 (0.218 - 0.248)
C12:1	0.111 (0.092 - 0.134)	0.094 (0.081 - 0.113)	0.127 (0.103 - 0.152)	0.115 (0.092 - 0.141)	0.12 (0.102 - 0.137)	0.107 (0.093 - 0.127)
C14	0.057 (0.051 - 0.062)	0.055 (0.048 - 0.06)	0.06 (0.055 - 0.067)	0.056 (0.051 - 0.063)	0.057 (0.05 - 0.063)	0.056 (0.05 - 0.06)
C14:1	0.04 (0.03 - 0.0585)	0.03 (0.024 - 0.046)	0.048 (0.037 - 0.063)	0.042 (0.028 - 0.057)	0.048 (0.0335 - 0.064)	0.041 (0.029 - 0.0535)
C14:1-OH	0.017 (0.015 - 0.02)	0.016 (0.015 - 0.018)	0.019 (0.016 - 0.021)	0.017 (0.015 - 0.02)	0.018 (0.016 - 0.021)	0.017 (0.015 - 0.019)
C14:2	0.021 (0.016 - 0.03)	0.016 (0.013 - 0.021)	0.024 (0.019 - 0.032)	0.02 (0.014 - 0.027)	0.022 (0.016 - 0.027)	0.022 (0.0175 - 0.029)
C14:2-OH	0.02 (0.0175 - 0.022)	0.02 (0.017 - 0.022)	0.02 (0.018 - 0.023)	0.02 (0.016 - 0.022)	0.02 (0.017 - 0.022)	0.02 (0.0185 - 0.023)
C16	0.093 (0.08 - 0.11)	0.082 (0.072 - 0.095)	0.093 (0.079 - 0.11)	0.08 (0.066 - 0.095)	0.103 (0.09 - 0.119)	0.084 (0.071 - 0.0945)
C16-OH	0.044 (0.039 - 0.05)	0.044 (0.04 - 0.049)	0.046 (0.039 - 0.05)	0.044 (0.037 - 0.049)	0.045 (0.04 - 0.05)	0.046 (0.042 - 0.051)
C16:1	0.026 (0.022 - 0.03)	0.024 (0.021 - 0.027)	0.027 (0.024 - 0.032)	0.026 (0.022 - 0.031)	0.028 (0.024 - 0.033)	0.025 (0.022 - 0.029)
C16:1-OH	0.014 (0.012 - 0.016)	0.013 (0.012 - 0.015)	0.015 (0.013 - 0.016)	0.014 (0.012 - 0.016)	0.015 (0.013 - 0.017)	0.013 (0.012 - 0.015)
C16:2	0.015 (0.013 - 0.018)	0.013 (0.012 - 0.016)	0.015 (0.013 - 0.017)	0.015 (0.012 - 0.016)	0.015 (0.013 - 0.018)	0.015 (0.013 - 0.017)
C16:2-OH	0.026 (0.024 - 0.029)	0.025 (0.023 - 0.027)	0.027 (0.025 - 0.029)	0.026 (0.023 - 0.027)	0.026 (0.024 - 0.028)	0.026 (0.024 - 0.028)
C18	0.042 (0.036 - 0.051)	0.039 (0.034 - 0.046)	0.044 (0.037 - 0.05)	0.04 (0.034 - 0.046)	0.044 (0.038 - 0.052)	0.039 (0.033 - 0.045)
C18:1	0.096 (0.083 - 0.12)	0.078 (0.068 - 0.092)	0.092 (0.076 - 0.112)	0.087 (0.073 - 0.104)	0.116 (0.0915 - 0.138)	0.092 (0.0785 - 0.111)
C18:2	0.041 (0.0345 - 0.0505)	0.03 (0.026 - 0.034)	0.037 (0.031 - 0.043)	0.031 (0.027 - 0.037)	0.04 (0.033 - 0.046)	0.04 (0.034 - 0.049)
C2	4.76 (3.66 - 6.72)	4.17 (3.49 - 5.17)	5.73 (4.52 - 7.72)	4.97 (3.75 - 6.92)	6.15 (4.45 - 9.1)	4.99 (3.88 - 6.59)
C3	0.207 (0.16 - 0.258)	0.23 (0.185 - 0.283)	0.228 (0.179 - 0.277)	0.207 (0.166 - 0.263)	0.216 (0.17 - 0.293)	0.247 (0.198 - 0.31)
C3-DC (C4-OH)	0.034 (0.028 - 0.042)	0.029 (0.025 - 0.035)	0.032 (0.027 - 0.04)	0.03 (0.025 - 0.037)	0.035 (0.03 - 0.045)	0.029 (0.025 - 0.036)
C3-OH	0.023 (0.019 - 0.029)	0.022 (0.018 - 0.028)	0.022 (0.019 - 0.028)	0.023 (0.018 - 0.028)	0.023 (0.019 - 0.029)	0.022 (0.019 - 0.029)
C4	0.103 (0.08 - 0.133)	0.111 (0.088 - 0.138)	0.108 (0.086 - 0.14)	0.117 (0.091 - 0.149)	0.102 (0.082 - 0.138)	0.119 (0.0915 - 0.15)
C4:1	0.021 (0.018 - 0.023)	0.021 (0.018 - 0.024)	0.021 (0.018 - 0.023)	0.021 (0.018 - 0.024)	0.021 (0.019 - 0.024)	0.021 (0.018 - 0.023)
C6 (C4:1-DC)	0.058 (0.0485 - 0.065)	0.052 (0.044 - 0.061)	0.063 (0.053 - 0.073)	0.055 (0.048 - 0.065)	0.058 (0.0505 - 0.066)	0.055 (0.047 - 0.062)
C5	0.111 (0.0905 - 0.136)	0.124 (0.104 - 0.153)	0.119 (0.098 - 0.151)	0.119 (0.101 - 0.15)	0.124 (0.101 - 0.154)	0.133 (0.111 - 0.17)
C5-M-DC	0.029 (0.026 - 0.032)	0.028 (0.025 - 0.031)	0.029 (0.026 - 0.033)	0.03 (0.027 - 0.033)	0.03 (0.026 - 0.034)	0.028 (0.025 - 0.031)
C5-OH (C3-DC-M)	0.062 (0.058 - 0.067)	0.064 (0.059 - 0.069)	0.063 (0.059 - 0.068)	0.064 (0.059 - 0.069)	0.065 (0.06 - 0.0695)	0.065 (0.06 - 0.069)
C5:1	0.04 (0.036 - 0.045)	0.041 (0.036 - 0.045)	0.042 (0.037 - 0.046)	0.043 (0.038 - 0.049)	0.043 (0.039 - 0.047)	0.042 (0.038 - 0.046)
C5:1-DC	0.027 (0.024 - 0.0305)	0.027 (0.024 - 0.032)	0.027 (0.024 - 0.032)	0.028 (0.024 - 0.031)	0.027 (0.024 - 0.031)	0.027 (0.024 - 0.032)
C5-DC (C6-OH)	0.023 (0.021 - 0.026)	0.022 (0.02 - 0.024)	0.023 (0.021 - 0.026)	0.023 (0.02 - 0.026)	0.022 (0.02 - 0.025)	0.022 (0.02 - 0.024)
C6:1	0.022 (0.019 - 0.024)	0.02 (0.018 - 0.022)	0.021 (0.019 - 0.024)	0.021 (0.019 - 0.024)	0.02 (0.018 - 0.022)	0.02 (0.018 - 0.022)
C7-DC	0.055 (0.047 - 0.062)	0.048 (0.044 - 0.055)	0.054 (0.049 - 0.062)	0.051 (0.046 - 0.058)	0.051 (0.046 - 0.0575)	0.051 (0.046 - 0.0575)
C8	0.137 (0.118 - 0.161)	0.12 (0.107 - 0.142)	0.148 (0.129 - 0.167)	0.135 (0.114 - 0.16)	0.134 (0.118 - 0.152)	0.129 (0.11 - 0.146)
C9	0.061 (0.054 - 0.069)	0.056 (0.05 - 0.062)	0.063 (0.057 - 0.073)	0.066 (0.058 - 0.078)	0.058 (0.053 - 0.065)	0.055 (0.0495 - 0.061)
Ala	350 (292 - 400)	356 (305 - 404)	319 (271 - 381)	304 (260 - 358)	322 (273 - 384)	343 (300 - 392)
Arg	102 (89.4 - 118)	107 (94 - 120)	100 (86.7 - 116)	97.2 (88.1 - 112)	113 (98.5 - 130)	103 (91.2 - 120)
Asn	46.8 (40.8 - 54.4)	49.1 (42.5 - 56.4)	41 (35.6 - 49.9)	44.7 (39.7 - 50.5)	49.9 (42.3 - 60.2)	46.7 (40.4 - 54.5)
Asp	16 (12.8 - 18.9)	10.6 (8.8 - 12.7)	11.4 (9.84 - 13.2)	11.2 (9.54 - 13)	16.8 (14.2 - 20)	12 (10.2 - 14.5)
Cit	33.3 (28.7 - 37.5)	28.8 (24.8 - 32.4)	30.3 (26.9 - 34.7)	30.4 (26.5 - 34.6)	34.5 (29.9 - 40.8)	29.3 (25.6 - 33.6)
Gln	759 (680 - 864)	771 (708 - 847)	701 (638 - 775)	781 (735 - 858)	772 (700 - 849)	701 (635 - 778)
Glu	53.1 (36.4 - 70.1)	37.4 (26.2 - 49.3)	61.5 (51.1 - 72.5)	38.4 (29.5 - 45.1)	60.5 (50.9 - 71.2)	45.1 (37.3 - 57.6)
Gly	235 (201 - 273)	216 (194 - 239)	219 (187 - 250)	212 (190 - 244)	207 (181 - 236)	202 (178 - 231)
His	86.8 (79 - 96.4)	87.1 (80.2 - 96.8)	84 (77.2 - 94.3)	85.1 (79.4 - 92.5)	90.4 (80.8 - 97.4)	85.6 (76.5 - 95.4)
Ile	66.1 (57.2 - 82.7)	72.5 (60.1 - 94.8)	72.5 (59.7 - 93.9)	70.4 (61.2 - 83.9)	93.5 (74.3 - 123)	79.8 (62 - 104)
Leu	119 (105 - 150)	132 (112 - 164)	127 (106 - 159)	128 (112 - 149)	157 (130 - 204)	141 (114 - 170)

µmol/L (median with IQR)	**BiB** (n = 199)	**EDEN** (n = 157)	**KANC** (n = 201)	**MoBa** (n = 229)	**Rhea** (n = 199)	**INMA** (n = 207)
Lys	161 (132 - 194)	192 (169 - 229)	174 (145 - 208)	165 (144 - 195)	175 (144 - 222)	190 (162 - 228)
Met	18.1 (15.2 - 22.7)	20.3 (17.1 - 24.7)	18 (15 - 23)	17.9 (15.9 - 21.6)	20 (15.8 - 26.2)	19.7 (16 - 25.4)
Orn	61.2 (52.6 - 73)	53.1 (48.1 - 64.6)	55.4 (44.7 - 66.6)	53.5 (46 - 61.3)	67.5 (54.9 - 81.3)	57.8 (48 - 68.2)
Phe	65.6 (57.1 - 77.3)	64.2 (55.9 - 72.1)	61.3 (54.3 - 71.1)	61.6 (55.1 - 69.8)	75.1 (65.8 - 84.6)	67.8 (58.4 - 79.1)
Pro	189 (144 - 231)	192 (166 - 239)	186 (151 - 233)	184 (151 - 230)	205 (172 - 250)	193 (156 - 244)
Ser	135 (123 - 153)	124 (112 - 141)	135 (120 - 152)	130 (118 - 143)	145 (130 - 168)	129 (113 - 148)
Thr	107 (90.5 - 125)	127 (108 - 149)	105 (88.3 - 123)	107 (94.2 - 121)	116 (98.1 - 144)	123 (107 - 141)
Trp	63.4 (54.8 - 77.3)	67.9 (60.1 - 80.8)	66.5 (58.6 - 77.2)	69.4 (61 - 75.1)	77.3 (65.6 - 89.1)	72.1 (61.5 - 80.2)
Tyr	70.6 (59.2 - 89.9)	72.8 (62.8 - 88.8)	69.4 (59.5 - 88.7)	69.5 (57.6 - 81.1)	84.6 (68.4 - 102)	78.2 (63.8 - 90.6)
Val	208 (178 - 250)	230 (203 - 270)	223 (191 - 267)	222 (198 - 256)	272 (222 - 318)	247 (206 - 287)
Ac-Orn	0.569 (0.389 - 0.81)	0.571 (0.372 - 0.765)	0.476 (0.349 - 0.696)	0.482 (0.313 - 0.642)	1.2 (0.611 - 2.02)	0.642 (0.39 - 1.26)
ADMA	0.581 (0.528 - 0.636)	0.55 (0.501 - 0.613)	0.595 (0.529 - 0.656)	0.531 (0.495 - 0.585)	0.597 (0.544 - 0.666)	0.542 (0.49 - 0.607)
alpha-AAA	0.73 (0.554 - 0.97)	0.867 (0.669 - 1.16)	0.847 (0.663 - 1.08)	0.809 (0.631 - 0.989)	0.988 (0.708 - 1.37)	1.01 (0.752 - 1.31)
Creatinine	37.4 (33.4 - 42.8)	47.5 (42.6 - 52.4)	38.2 (34.5 - 42.2)	42 (37.8 - 45.6)	39.6 (35.7 - 46.8)	42.8 (37.4 - 47.6)
Kynurenine	2.4 (2.06 - 2.9)	2.4 (2.12 - 2.98)	2.59 (2.31 - 2.97)	2.5 (2.16 - 2.93)	2.53 (2.2 - 2.98)	2.47 (2.17 - 2.84)
Met-SO	1.03 (0.732 - 1.38)	0.734 (0.61 - 0.91)	1.23 (0.97 - 1.48)	0.778 (0.673 - 0.967)	1.28 (0.97 - 1.61)	0.945 (0.772 - 1.22)
Putrescine	0.106 (0.078 - 0.134)	0.092 (0.074 - 0.123)	0.096 (0.074 - 0.12)	0.099 (0.076 - 0.13)	0.111 (0.0875 - 0.139)	0.096 (0.071 - 0.12)
SDMA	0.395 (0.289 - 0.498)	0.384 (0.279 - 0.477)	0.426 (0.332 - 0.511)	0.4 (0.306 - 0.508)	0.399 (0.302 - 0.488)	0.323 (0.25 - 0.44)
Serotonin	1.34 (0.948 - 1.73)	0.775 (0.488 - 1.13)	0.697 (0.502 - 0.975)	0.995 (0.628 - 1.41)	1.16 (0.852 - 1.56)	0.923 (0.604 - 1.23)
Spermidine	0.163 (0.146 - 0.194)	0.164 (0.145 - 0.176)	0.155 (0.138 - 0.177)	0.163 (0.142 - 0.184)	0.174 (0.153 - 0.2)	0.152 (0.136 - 0.168)
Spermine	0.214 (0.2 - 0.228)	0.208 (0.197 - 0.222)	0.213 (0.203 - 0.228)	0.21 (0.192 - 0.224)	0.223 (0.207 - 0.236)	0.208 (0.18 - 0.218)
t4-OH-Pro	14.9 (12.9 - 18.4)	17.3 (13.6 - 20.6)	17.1 (14 - 21.9)	15.6 (13.1 - 19.2)	15.9 (13.1 - 20.7)	17.6 (14.2 - 22.6)
Taurine	89 (64.2 - 114)	54.2 (47.3 - 66.9)	53.2 (46.9 - 59.6)	61.6 (50.9 - 72.6)	90.7 (67.1 - 114)	58 (50.6 - 76.2)
total DMA	0.831 (0.763 - 0.929)	0.798 (0.72 - 0.878)	0.875 (0.796 - 0.932)	0.804 (0.739 - 0.894)	0.822 (0.764 - 0.911)	0.749 (0.678 - 0.832)
lysoPC a C14:0	2.94 (2.68 - 3.24)	3.09 (2.78 - 3.42)	2.94 (2.68 - 3.3)	3.03 (2.75 - 3.35)	2.93 (2.67 - 3.24)	2.75 (2.58 - 3.06)
lysoPC a C16:0	65.9 (58.6 - 81.4)	61.6 (54.2 - 71.9)	60.9 (52.3 - 70.3)	61.3 (52.6 - 69.5)	68.6 (59.8 - 77.8)	60.9 (54 - 70.8)
lysoPC a C16:1	1.71 (1.38 - 2.13)	1.8 (1.54 - 2.18)	1.62 (1.4 - 1.98)	1.78 (1.48 - 2.11)	1.69 (1.39 - 2.1)	1.59 (1.31 - 1.8)
lysoPC a C17:0	1.15 (0.965 - 1.41)	1.22 (1.04 - 1.41)	1.11 (0.904 - 1.37)	1.27 (1.1 - 1.46)	1.04 (0.878 - 1.26)	0.999 (0.823 - 1.16)
lysoPC a C18:0	21.3 (18.4 - 25.7)	19.8 (16.9 - 22.8)	20 (16.4 - 22.6)	19.8 (17.4 - 22.4)	21 (18.2 - 24.2)	20.6 (17.5 - 24)
lysoPC a C18:1	18.7 (14.9 - 23.5)	16.6 (13.7 - 20.1)	15.6 (13.1 - 19)	17.1 (14.2 - 19.9)	21.9 (18.2 - 26.4)	17 (13.8 - 20.6)
lysoPC a C18:2	39.8 (27.8 - 51.1)	33.8 (25.3 - 41.9)	33.3 (26.5 - 41.6)	32.3 (25.6 - 38.4)	44.5 (35.7 - 55.8)	38.7 (27.6 - 49.8)
lysoPC a C20:3	2.18 (1.62 - 2.76)	1.97 (1.55 - 2.6)	1.74 (1.4 - 2.18)	1.81 (1.44 - 2.25)	2.76 (2.29 - 3.24)	2.24 (1.72 - 2.64)
lysoPC a C20:4	5.42 (4.16 - 6.66)	5.05 (4.29 - 6.1)	4.91 (4.08 - 5.65)	4.54 (3.97 - 5.6)	6.48 (5.26 - 7.62)	5.89 (4.55 - 7.14)
lysoPC a C24:0	0.185 (0.152 - 0.226)	0.18 (0.146 - 0.226)	0.177 (0.149 - 0.217)	0.194 (0.159 - 0.256)	0.17 (0.141 - 0.208)	0.172 (0.139 - 0.22)
lysoPC a C26:0	0.335 (0.26 - 0.466)	0.328 (0.235 - 0.464)	0.303 (0.242 - 0.443)	0.307 (0.245 - 0.46)	0.34 (0.266 - 0.479)	0.314 (0.236 - 0.422)
lysoPC a C26:1	0.176 (0.128 - 0.234)	0.167 (0.122 - 0.23)	0.163 (0.127 - 0.23)	0.185 (0.133 - 0.245)	0.165 (0.126 - 0.234)	0.166 (0.121 - 0.226)
lysoPC a C28:0	0.36 (0.3 - 0.466)	0.35 (0.291 - 0.451)	0.34 (0.287 - 0.438)	0.357 (0.295 - 0.462)	0.362 (0.316 - 0.476)	0.351 (0.289 - 0.43)
lysoPC a C28:1	0.357 (0.29 - 0.442)	0.373 (0.315 - 0.475)	0.366 (0.296 - 0.456)	0.384 (0.32 - 0.473)	0.35 (0.284 - 0.446)	0.35 (0.279 - 0.408)
PC aa C24:0	0.148 (0.116 - 0.218)	0.146 (0.115 - 0.201)	0.146 (0.113 - 0.195)	0.148 (0.117 - 0.206)	0.152 (0.112 - 0.198)	0.154 (0.118 - 0.198)
PC aa C28:1	2.11 (1.74 - 2.52)	2.4 (2.07 - 2.81)	2.13 (1.87 - 2.62)	2.53 (2.21 - 2.83)	2.13 (1.84 - 2.54)	2.05 (1.74 - 2.46)
PC aa C30:0	3.32 (2.71 - 3.88)	3.67 (3.18 - 4.43)	3.41 (2.86 - 4.21)	3.59 (3.04 - 4.45)	3.41 (2.9 - 4.24)	2.94 (2.37 - 3.52)
PC aa C30:2	0.13 (0.053 - 0.202)	0.114 (0.049 - 0.187)	0.113 (0.036 - 0.199)	0.141 (0.06 - 0.195)	0.117 (0.053 - 0.206)	0.128 (0.044 - 0.21)
PC aa C32:0	20.9 (18.4 - 24.5)	21.3 (19.1 - 23.9)	21.4 (19.1 - 24.5)	21.5 (18.9 - 24)	23.5 (21.2 - 26.8)	20.6 (17.8 - 23.2)
PC aa C32:1	19.9 (14.9 - 25)	22.1 (18.3 - 27.9)	20.1 (16.5 - 25.6)	22 (17.3 - 28.4)	19.4 (15.2 - 25.2)	16.7 (12.4 - 21.2)
PC aa C32:2	6.4 (5.07 - 7.96)	6.87 (5.41 - 9.04)	6.42 (4.99 - 8.02)	6.62 (5.05 - 8.13)	6.11 (4.65 - 7.4)	6.06 (4.72 - 7.92)
PC aa C32:3	0.788 (0.68 - 0.966)	0.842 (0.711 - 1.03)	0.801 (0.702 - 0.918)	0.888 (0.771 - 1.08)	0.734 (0.632 - 0.84)	0.732 (0.638 - 0.86)
PC aa C34:1	323 (274 - 396)	325 (277 - 373)	304 (267 - 344)	326 (276 - 379)	369 (306 - 418)	289 (251 - 342)
PC aa C34:2	807 (699 - 948)	759 (656 - 856)	788 (685 - 882)	707 (624 - 806)	870 (754 - 994)	799 (704 - 914)
PC aa C34:3	26.8 (22.2 - 32.4)	24.1 (19.8 - 29.7)	23.5 (19.5 - 27.7)	25.2 (21.7 - 30.1)	21.3 (18 - 25)	19 (16.8 - 23.3)
PC aa C34:4	2.52 (1.96 - 3)	2.81 (2.27 - 3.61)	2.39 (1.91 - 2.95)	2.66 (2.15 - 3.29)	2.41 (1.94 - 3)	2.53 (1.99 - 3.06)
PC aa C36:0	3.6 (3.08 - 4.34)	4.08 (3.56 - 4.86)	3.87 (3.21 - 4.63)	4.58 (3.94 - 5.51)	3.58 (2.9 - 4.27)	3.89 (3.33 - 4.54)

µmol/L (median with IQR)	**BiB** (n = 199)	**EDEN** (n = 157)	**KANC** (n = 201)	**MoBa** (n = 229)	**Rhea** (n = 199)	**INMA** (n = 207)
PC aa C36:1	59.2 (49.7 - 72.2)	61.5 (52.3 - 72.3)	56.1 (46.8 - 66.8)	65.4 (54.3 - 76.2)	66.6 (55.6 - 78.2)	57.7 (48.8 - 67.3)
PC aa C36:2	423 (364 - 502)	408 (350 - 455)	419 (363 - 468)	398 (354 - 453)	440 (390 - 494)	442 (392 - 506)
PC aa C36:3	198 (174 - 233)	194 (164 - 219)	186 (159 - 212)	185 (165 - 215)	220 (194 - 252)	204 (178 - 234)
PC aa C36:4	255 (212 - 296)	257 (229 - 294)	246 (218 - 275)	231 (205 - 267)	283 (241 - 332)	277 (234 - 326)
PC aa C36:5	25.5 (19.5 - 33.8)	25.2 (20.3 - 32.4)	20.6 (16.2 - 26.7)	36.2 (28.5 - 54.2)	16.1 (12.6 - 20.7)	14.6 (11.1 - 19.8)
PC aa C36:6	1.11 (0.882 - 1.48)	1.42 (1.14 - 1.75)	1.07 (0.851 - 1.42)	1.77 (1.41 - 2.27)	0.897 (0.734 - 1.1)	1 (0.799 - 1.2)
PC aa C38:0	3.21 (2.59 - 3.91)	3.81 (3.14 - 4.45)	3.63 (3.12 - 4.44)	4.69 (3.99 - 5.43)	3.19 (2.66 - 3.8)	3.98 (3.2 - 4.58)
PC aa C38:1	1.21 (1.01 - 1.54)	1.24 (0.991 - 1.54)	1.2 (0.978 - 1.48)	1.67 (1.38 - 2.03)	1.16 (0.888 - 1.43)	1.14 (0.878 - 1.44)
PC aa C38:3	56.6 (46 - 66.7)	58.1 (49.2 - 69.9)	50.8 (42.9 - 58.8)	55.4 (48 - 65.3)	59.7 (52.1 - 71)	59.9 (51.3 - 70.4)
PC aa C38:4	129 (104 - 150)	133 (118 - 150)	126 (109 - 148)	124 (105 - 140)	137 (116 - 162)	149 (124 - 176)
PC aa C38:5	61.2 (50.7 - 73.6)	59.2 (52.4 - 67.4)	57 (48.9 - 67)	67.7 (58.1 - 78.3)	55.4 (47.4 - 63.8)	51.5 (43.2 - 62.8)
PC aa C38:6	80.8 (66.4 - 98.6)	95.2 (79.5 - 111)	80.5 (67.1 - 98.2)	118 (98.5 - 142)	69.8 (58.6 - 86.6)	80.7 (67.4 - 95.4)
PC aa C40:1	0.494 (0.441 - 0.568)	0.472 (0.428 - 0.532)	0.497 (0.455 - 0.551)	0.564 (0.503 - 0.628)	0.466 (0.414 - 0.51)	0.499 (0.444 - 0.562)
PC aa C40:2	0.391 (0.332 - 0.46)	0.354 (0.309 - 0.4)	0.361 (0.33 - 0.407)	0.404 (0.353 - 0.473)	0.356 (0.322 - 0.406)	0.367 (0.317 - 0.431)
PC aa C40:3	0.593 (0.496 - 0.677)	0.544 (0.488 - 0.61)	0.54 (0.471 - 0.6)	0.613 (0.557 - 0.696)	0.546 (0.49 - 0.625)	0.537 (0.469 - 0.61)
PC aa C40:4	3.73 (3.12 - 4.42)	3.69 (3.19 - 4.37)	3.8 (3.3 - 4.35)	3.54 (3.1 - 4.1)	4.05 (3.46 - 4.68)	4.12 (3.42 - 4.88)
PC aa C40:5	9.82 (7.92 - 11.7)	9.52 (8.08 - 11.2)	9.31 (8.11 - 10.7)	9.48 (8.09 - 11.3)	8.45 (7.38 - 9.86)	7.98 (6.58 - 9.59)
PC aa C40:6	24.7 (20.4 - 30.2)	30 (24.2 - 34.6)	24.6 (20.2 - 29.8)	36.2 (30.5 - 44.3)	20.3 (17.2 - 25)	25.2 (20.6 - 29.4)
PC aa C42:0	0.539 (0.444 - 0.65)	0.551 (0.485 - 0.634)	0.557 (0.484 - 0.64)	0.647 (0.555 - 0.764)	0.506 (0.432 - 0.604)	0.552 (0.474 - 0.653)
PC aa C42:1	0.286 (0.244 - 0.34)	0.288 (0.24 - 0.333)	0.29 (0.248 - 0.336)	0.328 (0.276 - 0.388)	0.261 (0.226 - 0.308)	0.308 (0.255 - 0.36)
PC aa C42:2	0.24 (0.204 - 0.277)	0.226 (0.201 - 0.252)	0.215 (0.188 - 0.25)	0.252 (0.229 - 0.293)	0.203 (0.183 - 0.24)	0.233 (0.196 - 0.264)
PC aa C42:4	0.171 (0.152 - 0.202)	0.172 (0.15 - 0.193)	0.174 (0.156 - 0.196)	0.171 (0.151 - 0.193)	0.177 (0.154 - 0.199)	0.181 (0.156 - 0.208)
PC aa C42:5	0.35 (0.292 - 0.428)	0.322 (0.281 - 0.372)	0.341 (0.305 - 0.384)	0.371 (0.326 - 0.431)	0.277 (0.236 - 0.31)	0.261 (0.226 - 0.3)
PC aa C42:6	0.554 (0.489 - 0.643)	0.517 (0.457 - 0.601)	0.52 (0.453 - 0.614)	0.675 (0.602 - 0.782)	0.45 (0.394 - 0.514)	0.451 (0.398 - 0.515)
PC ae C30:0	0.347 (0.294 - 0.422)	0.436 (0.373 - 0.505)	0.416 (0.357 - 0.495)	0.423 (0.348 - 0.492)	0.371 (0.318 - 0.443)	0.327 (0.278 - 0.396)
PC ae C30:1	0.24 (0.179 - 0.366)	0.289 (0.204 - 0.369)	0.268 (0.178 - 0.357)	0.291 (0.212 - 0.38)	0.258 (0.201 - 0.35)	0.254 (0.19 - 0.344)
PC ae C30:2	0.088 (0.072 - 0.112)	0.098 (0.084 - 0.12)	0.092 (0.078 - 0.11)	0.099 (0.084 - 0.115)	0.084 (0.07 - 0.105)	0.088 (0.075 - 0.105)
PC ae C32:1	4.55 (3.74 - 5.34)	4.99 (4.4 - 5.63)	4.82 (4.35 - 5.63)	4.72 (4.02 - 5.58)	5.3 (4.28 - 5.92)	4.73 (4.11 - 5.38)
PC ae C32:2	1.04 (0.851 - 1.24)	1.2 (1.04 - 1.35)	1.1 (0.983 - 1.29)	1.23 (1.08 - 1.42)	1.09 (0.944 - 1.31)	1.12 (0.968 - 1.29)
PC ae C34:0	1.93 (1.6 - 2.36)	2.35 (1.96 - 2.79)	2.12 (1.78 - 2.52)	2.36 (2.01 - 2.76)	2.02 (1.73 - 2.4)	1.83 (1.49 - 2.16)
PC ae C34:1	16 (13.1 - 18.6)	16.9 (15 - 19.4)	16.6 (14.7 - 19.1)	17.1 (15.1 - 19.1)	17.8 (15.3 - 19.8)	15.5 (13 - 17.4)
PC ae C34:2	19 (15.9 - 22.6)	20.3 (17.6 - 23.7)	21.8 (18.4 - 26)	19.4 (16.7 - 22.5)	21.7 (18.3 - 25.6)	22.5 (19 - 26.6)
PC ae C34:3	15.7 (13.2 - 19.2)	16.3 (13.9 - 18.8)	16.6 (14.3 - 19.6)	15.3 (12.9 - 18)	17.6 (14.4 - 20.6)	17.3 (13.8 - 20.4)
PC ae C36:0	1.18 (0.989 - 1.38)	1.27 (1.12 - 1.4)	1.16 (1.03 - 1.34)	1.36 (1.19 - 1.57)	1.24 (1.08 - 1.4)	1.16 (1 - 1.33)
PC ae C36:1	10.3 (8.49 - 12.6)	11.2 (9.94 - 13)	10.6 (8.89 - 12.1)	11.8 (10.2 - 13.4)	10.4 (8.72 - 12.2)	9.25 (8.06 - 10.7)
PC ae C36:2	21 (17.6 - 25.3)	21.8 (19.6 - 25.6)	22.8 (19.4 - 25.6)	23 (20.1 - 25.9)	20.7 (18 - 24.2)	20.4 (17.8 - 23.4)
PC ae C36:3	12.5 (10.6 - 15.2)	13.2 (11.7 - 14.9)	13.7 (11.8 - 16)	13.3 (11.7 - 15.2)	14.4 (12.2 - 17.3)	14.8 (12.6 - 17.3)
PC ae C36:4	24.1 (20.8 - 28.2)	29.2 (25.7 - 33.2)	30 (25.7 - 36.2)	24.9 (21.6 - 29.8)	31.7 (25.8 - 37)	33.7 (28.4 - 40.2)
PC ae C36:5	16.2 (13.2 - 18.8)	19.5 (15.9 - 23.1)	18.1 (15.2 - 21.9)	17.2 (14.8 - 20.8)	19 (15.6 - 22.4)	20.5 (16.5 - 23.7)
PC ae C38:0	2.29 (1.94 - 2.83)	2.66 (2.22 - 2.9)	2.22 (1.84 - 2.62)	3.27 (2.74 - 4.04)	1.84 (1.62 - 2.2)	2.03 (1.69 - 2.31)
PC ae C38:1	0.728 (0.566 - 0.995)	0.694 (0.563 - 0.876)	0.655 (0.513 - 0.821)	0.862 (0.677 - 1.05)	0.631 (0.463 - 0.838)	0.663 (0.484 - 0.876)
PC ae C38:2	2.91 (2.48 - 3.56)	2.81 (2.38 - 3.17)	2.94 (2.46 - 3.43)	3.08 (2.66 - 3.43)	2.98 (2.56 - 3.34)	2.79 (2.44 - 3.22)
PC ae C38:3	5.39 (4.38 - 6.36)	5.59 (4.98 - 6.53)	5.15 (4.53 - 6.05)	5.72 (4.97 - 6.44)	5.56 (4.66 - 6.33)	5.26 (4.54 - 5.92)
PC ae C38:4	17.8 (15.2 - 20.8)	20.1 (18 - 22.4)	20.6 (17.9 - 23.5)	18.8 (16.1 - 21.6)	20.7 (17.5 - 24.1)	20.9 (17.6 - 24)
PC ae C38:5	22.9 (19.8 - 27)	25.5 (22.9 - 28.9)	26.9 (23.2 - 30.9)	25.7 (22.1 - 29.6)	28 (23.6 - 32.9)	29.4 (24.7 - 35.2)
PC ae C38:6	8.35 (7 - 10.2)	10.5 (8.91 - 12.1)	9.7 (8.38 - 12.2)	11.4 (9.51 - 13.4)	8.94 (7.28 - 10.7)	10.5 (8.43 - 12.4)
PC ae C40:1	1.41 (1.19 - 1.76)	1.5 (1.31 - 1.73)	1.35 (1.09 - 1.55)	1.7 (1.45 - 1.93)	1.37 (1.17 - 1.67)	1.46 (1.26 - 1.73)
PC ae C40:2	1.62 (1.36 - 1.94)	1.66 (1.46 - 1.86)	1.59 (1.39 - 1.86)	1.94 (1.68 - 2.28)	1.43 (1.24 - 1.62)	1.3 (1.12 - 1.46)
PC ae C40:3	1.23 (1.02 - 1.43)	1.15 (1.04 - 1.3)	1.17 (1.03 - 1.29)	1.24 (1.13 - 1.41)	1.19 (1.07 - 1.34)	1.16 (1 - 1.32)
PC ae C40:4	2.7 (2.31 - 3.2)	2.87 (2.46 - 3.13)	2.92 (2.62 - 3.34)	2.68 (2.35 - 3.06)	3 (2.56 - 3.45)	2.89 (2.51 - 3.34)
PC ae C40:5	4.32 (3.62 - 4.98)	4.36 (3.81 - 4.93)	4.57 (4.09 - 5.23)	4.58 (4.09 - 5.26)	4.46 (3.84 - 5.02)	4.5 (3.74 - 5.13)

µmol/L (median with IQR)	**BiB** (n = 199)	**EDEN** (n = 157)	**KANC** (n = 201)	**MoBa** (n = 229)	**Rhea** (n = 199)	**INMA** (n = 207)
PC ae C40:6	5.13 (4.07 - 5.75)	5.89 (5.03 - 6.79)	5.57 (4.93 - 6.64)	7.01 (6.05 - 8.27)	4.71 (4.06 - 5.58)	5.24 (4.53 - 6.06)
PC ae C42:0	0.837 (0.79 - 0.928)	0.817 (0.759 - 0.874)	0.822 (0.763 - 0.886)	0.913 (0.854 - 0.984)	0.789 (0.745 - 0.858)	0.816 (0.758 - 0.88)
PC ae C42:1	0.493 (0.424 - 0.594)	0.465 (0.407 - 0.535)	0.466 (0.405 - 0.541)	0.5 (0.433 - 0.565)	0.474 (0.417 - 0.559)	0.525 (0.444 - 0.605)
PC ae C42:2	0.591 (0.494 - 0.719)	0.579 (0.499 - 0.65)	0.569 (0.485 - 0.658)	0.676 (0.565 - 0.771)	0.556 (0.487 - 0.636)	0.511 (0.436 - 0.588)
PC ae C42:3	0.83 (0.704 - 0.981)	0.772 (0.683 - 0.884)	0.76 (0.664 - 0.87)	0.919 (0.799 - 1.06)	0.765 (0.639 - 0.884)	0.77 (0.658 - 0.88)
PC ae C42:4	0.857 (0.734 - 1.03)	0.859 (0.742 - 0.986)	0.911 (0.81 - 1.04)	0.852 (0.753 - 0.986)	0.906 (0.746 - 1.04)	0.871 (0.736 - 1.01)
PC ae C42:5	1.98 (1.65 - 2.36)	2.03 (1.82 - 2.3)	2.09 (1.82 - 2.42)	2.14 (1.87 - 2.48)	2.07 (1.78 - 2.37)	2.13 (1.75 - 2.44)
PC ae C44:3	0.158 (0.13 - 0.184)	0.149 (0.129 - 0.179)	0.149 (0.125 - 0.168)	0.163 (0.137 - 0.186)	0.144 (0.119 - 0.17)	0.148 (0.126 - 0.179)
PC ae C44:4	0.324 (0.284 - 0.39)	0.33 (0.298 - 0.379)	0.333 (0.288 - 0.381)	0.347 (0.299 - 0.393)	0.347 (0.3 - 0.4)	0.319 (0.278 - 0.373)
PC ae C44:5	1.33 (1.09 - 1.6)	1.43 (1.21 - 1.69)	1.41 (1.2 - 1.63)	1.43 (1.23 - 1.73)	1.44 (1.22 - 1.68)	1.44 (1.16 - 1.68)
PC ae C44:6	0.968 (0.815 - 1.17)	0.979 (0.821 - 1.17)	1.07 (0.894 - 1.24)	1.07 (0.902 - 1.26)	0.945 (0.806 - 1.15)	1.13 (0.939 - 1.3)
SM (OH) C14:1	4.21 (3.4 - 4.86)	5.17 (4.48 - 6.01)	4.84 (4.05 - 5.6)	4.77 (4.18 - 5.4)	4.14 (3.61 - 4.95)	4.05 (3.4 - 4.54)
SM (OH) C16:1	2.14 (1.82 - 2.55)	2.77 (2.39 - 3.23)	2.58 (2.16 - 2.97)	2.71 (2.3 - 3.04)	2.19 (1.88 - 2.57)	2.19 (1.88 - 2.5)
SM (OH) C22:1	6.74 (5.59 - 7.98)	7.29 (6.24 - 8.5)	7.38 (6.22 - 8.91)	6.54 (5.6 - 7.85)	8.22 (6.95 - 9.55)	6.97 (5.92 - 8.32)
SM (OH) C22:2	5.48 (4.46 - 6.38)	6.2 (5.2 - 7.15)	5.66 (4.74 - 6.95)	5.87 (5.02 - 6.98)	5.67 (4.8 - 6.62)	5.27 (4.34 - 6.14)
SM (OH) C24:1	0.542 (0.452 - 0.65)	0.587 (0.487 - 0.71)	0.584 (0.473 - 0.71)	0.577 (0.474 - 0.672)	0.608 (0.528 - 0.697)	0.534 (0.434 - 0.64)
SM C16:0	89.2 (78.3 - 102)	92.5 (83.2 - 102)	91 (83.1 - 102)	87.8 (80.6 - 97.7)	95.7 (84.7 - 106)	92.2 (80.6 - 102)
SM C16:1	11 (9.57 - 12.6)	12.1 (10.7 - 13.6)	11.1 (9.67 - 12.8)	11 (9.92 - 12.5)	11.7 (10.4 - 13.4)	12 (10.6 - 13.2)
SM C18:0	15.1 (13.1 - 17.8)	17.2 (15.3 - 19.9)	16.4 (14.2 - 19)	16.8 (14.4 - 19.6)	16.6 (14.4 - 18.9)	16.2 (13.1 - 18.5)
SM C18:1	7.16 (6.12 - 8.16)	8.47 (7.55 - 9.62)	7.77 (6.87 - 9.12)	7.87 (6.92 - 9.27)	7.77 (6.68 - 8.9)	8.11 (6.86 - 9.07)
SM C20:2	0.304 (0.248 - 0.362)	0.317 (0.264 - 0.373)	0.334 (0.292 - 0.4)	0.325 (0.28 - 0.388)	0.325 (0.269 - 0.384)	0.346 (0.286 - 0.42)
SM C24:0	10.5 (8.8 - 12.2)	10.1 (8.62 - 12.2)	10.6 (9.09 - 12.5)	9.81 (8.32 - 11.5)	11.2 (9.64 - 12.8)	10.6 (8.95 - 12.6)
SM C24:1	22 (17.3 - 25.7)	21.3 (18.3 - 24.2)	20.9 (17.6 - 24.6)	21.1 (18.5 - 26.1)	20.2 (17.1 - 23.4)	19.6 (15.8 - 23.2)
SM C26:0	0.076 (0.0605 - 0.09)	0.079 (0.065 - 0.097)	0.081 (0.065 - 0.101)	0.088 (0.072 - 0.107)	0.084 (0.067 - 0.1)	0.077 (0.055 - 0.094)
SM C26:1	0.135 (0.114 - 0.168)	0.145 (0.118 - 0.169)	0.148 (0.119 - 0.18)	0.178 (0.14 - 0.213)	0.138 (0.11 - 0.164)	0.132 (0.109 - 0.162)
H1	3880 (3530 - 4200)	4200 (3910 - 4630)	4080 (3840 - 4380)	4010 (3740 - 4280)	4100 (3820 - 4390)	3990 (3710 - 4340)

Supplementary Table 6 Metabolites associated with age

metabolite	effect size (SD/ year)	lower CI	upper CI	p-value	I^2
creatinine (urine)	0.39	0.26	0.53	4.4E-09	0%
Creatinine (serum)	0.30	0.17	0.43	7.3E-06	23%

Supplementary Table 7 Metabolites associated with sex

metabolite	effect size (SD)	lower CI	upper CI	p-value	I^2
isoleucine (urine)	-0.24	-0.37	-0.12	1.1E-04	59%
5-oxoproline (urine)	0.23	0.11	0.36	2.3E-04	0%
tyrosine (urine)	0.43	0.31	0.55	3.7E-12	9%
C10	-0.23	-0.35	-0.11	2.6E-04	29%
C12	-0.29	-0.41	-0.17	1.8E-06	27%
C14:1	-0.34	-0.46	-0.22	5.8E-08	19%
C14:1-OH	-0.22	-0.34	-0.10	2.6E-04	19%
C14:2	-0.24	-0.36	-0.12	7.9E-05	17%
C16:1	-0.26	-0.38	-0.14	3.0E-05	15%
Lys	-0.24	-0.35	-0.12	1.1E-04	53%
Orn	-0.35	-0.47	-0.23	1.4E-08	18%
Ser	-0.26	-0.39	-0.14	2.1E-05	5%
Putrescine	-0.21	-0.33	-0.10	2.8E-04	55%
Serotonin	0.32	0.20	0.44	1.8E-07	49%
SM C16:1	-0.35	-0.46	-0.24	3.2E-10	0%
SM C18:0	-0.22	-0.33	-0.11	9.2E-05	0%
SM C18:1	-0.35	-0.46	-0.24	1.8E-10	0%
H1	0.23	0.11	0.35	2.4E-04	8%

Metabolites found higher in male children are shown as positive and metabolites found higher in female children are shown as negative

Supplementary Table 8 Metabolites associated with z-BMI score

metabolite	effect size (SD/ unit z-score)	lower CI	upper CI	p-value	I^2
valine (urine)	0.09	0.04	0.15	4.1E-04	0%
4-deoxyerythronic acid (urine)	0.21	0.16	0.26	8.1E-16	40%
p-cresol sulfate (urine)	-0.10	-0.16	-0.05	1.1E-04	63%
pantothenate (urine)	-0.12	-0.17	-0.07	2.0E-06	13%
C0	0.18	0.13	0.24	1.4E-12	0%
C18	-0.13	-0.18	-0.08	1.1E-06	14%
C3	0.15	0.10	0.20	7.6E-10	0%
C5	0.13	0.08	0.18	1.0E-06	0%
Asp	0.10	0.05	0.15	1.5E-04	20%
Glu	0.22	0.17	0.27	3.3E-17	0%
Leu	0.11	0.06	0.16	4.3E-05	0%
Lys	0.10	0.05	0.15	2.1E-04	0%
Phe	0.12	0.07	0.17	3.4E-06	23%
Pro	0.10	0.05	0.15	1.2E-04	0%
Val	0.12	0.07	0.18	2.5E-06	0%
ADMA	0.09	0.04	0.15	2.7E-04	0%
alpha-AAA	0.10	0.05	0.15	8.7E-05	0%
Kynurenine	0.10	0.05	0.15	1.1E-04	36%
lysoPC a C14:0	0.09	0.04	0.14	2.6E-04	0%
lysoPC a C16:1	0.14	0.09	0.19	1.1E-07	0%
lysoPC a C18:1	-0.09	-0.14	-0.05	1.8E-04	0%
lysoPC a C18:2	-0.12	-0.17	-0.07	4.9E-06	0%
PC aa C32:1	0.11	0.06	0.16	1.4E-05	0%
PC aa C32:3	0.10	0.05	0.15	5.9E-05	0%
PC aa C34:4	0.14	0.08	0.19	2.3E-07	0%
PC aa C36:5	0.10	0.05	0.15	1.5E-04	0%
PC aa C36:6	0.10	0.04	0.15	2.6E-04	0%
PC aa C38:1	-0.10	-0.15	-0.05	8.2E-05	38%
PC aa C38:3	0.20	0.15	0.25	5.6E-16	0%
PC aa C38:4	0.14	0.08	0.19	1.5E-07	0%
PC aa C40:6	0.10	0.05	0.15	1.2E-04	0%
PC ae C34:0	-0.10	-0.14	-0.05	1.8E-04	0%
PC ae C34:1	-0.09	-0.14	-0.04	2.2E-04	0%
PC ae C36:0	-0.10	-0.15	-0.05	2.4E-05	0%
PC ae C36:2	-0.12	-0.17	-0.07	2.6E-06	0%
PC ae C38:2	-0.10	-0.15	-0.05	5.6E-05	3%
PC ae C40:4	-0.11	-0.16	-0.06	2.4E-05	23%
PC ae C40:5	-0.10	-0.15	-0.05	1.1E-04	12%
PC ae C40:6	-0.10	-0.15	-0.05	1.5E-04	0%
PC ae C42:3	-0.11	-0.16	-0.06	1.6E-05	0%
PC ae C42:4	-0.14	-0.19	-0.09	1.7E-07	0%
PC ae C44:4	-0.11	-0.16	-0.05	5.7E-05	21%
SM C16:0	-0.10	-0.14	-0.05	7.3E-05	18%
SM C16:1	0.16	0.11	0.21	1.3E-11	0%
SM C18:1	0.16	0.11	0.20	6.0E-11	0%

Supplementary Table 9 Metabolites associated with dietary intake

metabolite-diet pair	effect size (SD/ times per week)	lower CI	upper CI	p-value	I^2
creatine (urine) with meat	0.025	0.012	0.039	2.9E-04	0%
pantothenate (urine) with dairy	0.009	0.004	0.015	7.3E-04	39%
acetate (urine) with potatoes	0.045	0.023	0.068	1.0E-04	0%
hippurate (urine) with vegetables	0.021	0.011	0.031	2.0E-05	4%
leucine (urine) with fruits	-0.014	-0.022	-0.006	5.3E-04	0%
alanine (urine) with fruits	-0.014	-0.022	-0.006	7.7E-04	28%
glutamine (urine) with fruits	-0.013	-0.021	-0.005	9.3E-04	0%
scyllo-inositol (urine) with fruits	0.014	0.007	0.021	9.0E-05	61%
hippurate (urine) with fruits	0.026	0.018	0.034	4.4E-11	52%
proline betaine (urine) with fruits	0.017	0.009	0.025	3.9E-05	0%
N-methylnicotinic acid (urine) with fruits	0.017	0.009	0.025	4.1E-05	61%
3-hydroxybutyrate/ 3-aminoisobutyrate (urine) with beverages	-0.049	-0.067	-0.030	2.2E-07	48%
PC ae C36:3 with meat	0.024	0.011	0.037	2.1E-04	0%
PC ae C36:4 with meat	0.031	0.018	0.044	3.5E-06	5%
PC ae C36:5 with meat	0.026	0.013	0.040	1.1E-04	0%
PC ae C38:5 with meat	0.027	0.014	0.040	5.6E-05	23%
PC aa C36:0 with fish	0.091	0.063	0.119	9.6E-11	56%
PC aa C36:5 with fish	0.062	0.034	0.090	1.5E-05	44%
PC aa C36:6 with fish	0.086	0.058	0.114	1.2E-09	0%
PC aa C38:0 with fish	0.107	0.080	0.134	1.3E-14	74%
PC aa C38:6 with fish	0.112	0.084	0.140	1.9E-15	59%
PC aa C40:1 with fish	0.053	0.026	0.081	1.5E-04	0%
PC aa C40:4 with fish	-0.057	-0.084	-0.030	3.4E-05	21%
PC aa C40:6 with fish	0.099	0.071	0.127	2.5E-12	51%
PC aa C42:2 with fish	0.057	0.030	0.084	4.6E-05	13%
PC ae C38:0 with fish	0.084	0.057	0.112	1.5E-09	40%
PC ae C38:6 with fish	0.074	0.046	0.102	2.3E-07	57%
PC ae C40:6 with fish	0.086	0.058	0.113	1.4E-09	34%
PC aa C28:1 with dairy	0.015	0.010	0.021	2.6E-08	59%
PC aa C30:0 with dairy	0.012	0.007	0.017	1.2E-05	70%
PC aa C38:0 with dairy	-0.013	-0.018	-0.007	2.9E-06	0%
PC ae C30:0 with dairy	0.015	0.010	0.020	2.2E-08	74%
PC ae C38:6 with dairy	-0.010	-0.016	-0.005	2.7E-04	0%
SM (OH) C14:1 with dairy	0.013	0.007	0.018	2.7E-06	71%
PC ae C30:0 with potatoes	-0.042	-0.064	-0.020	1.7E-04	0%
PC ae C34:0 with potatoes	-0.049	-0.071	-0.027	1.2E-05	29%
SM (OH) C14:1 with potatoes	-0.049	-0.070	-0.027	1.2E-05	52%
Ac-Orn with fruits	0.016	0.008	0.024	5.7E-05	0%
PC aa C38:0 with sweets	-0.021	-0.031	-0.011	2.2E-05	0%
PC aa C38:6 with sweets	-0.020	-0.030	-0.010	8.6E-05	0%
PC ae C38:5 with sweets	-0.020	-0.030	-0.011	4.5E-05	0%
PC ae C38:6 with sweets	-0.020	-0.030	-0.010	1.2E-04	0%
PC ae C40:6 with sweets	-0.022	-0.032	-0.012	1.4E-05	0%
lysoPC a C17:0 with bakery products	-0.026	-0.039	-0.013	5.7E-05	56%
PC aa C36:6 with bakery products	-0.028	-0.041	-0.015	2.7E-05	0%
PC aa C38:0 with bakery products	-0.027	-0.040	-0.014	3.3E-05	52%
PC aa C38:6 with bakery products	-0.028	-0.041	-0.014	3.5E-05	52%
PC aa C40:6 with bakery products	-0.027	-0.040	-0.015	2.9E-05	41%
PC ae C38:0 with bakery products	-0.030	-0.043	-0.017	3.9E-06	15%
PC ae C38:6 with bakery products	-0.025	-0.038	-0.012	1.3E-04	14%
PC ae C40:2 with bakery products	-0.027	-0.040	-0.014	4.3E-05	53%
PC ae C40:6 with bakery products	-0.026	-0.039	-0.013	6.6E-05	36%
SM (OH) C14:1 with bakery products	-0.027	-0.040	-0.015	2.4E-05	27%
SM (OH) C16:1 with bakery products	-0.031	-0.043	-0.018	9.7E-07	43%
C5:1 with beverages	-0.032	-0.050	-0.015	2.7E-04	0%
C6:1 with beverages	-0.030	-0.046	-0.014	2.6E-04	15%
SM (OH) C16:1 with beverages	-0.032	-0.050	-0.015	2.3E-04	0%

Supplementary Table 10 Variance decomposition analysis of urine metabolites (% variance explained)

	analytical	pre-analytical	demographic	dietary	cohort	residual
creatinine	2.5	1.1	7.0	1.8	2.0	85.7
leucine	1.5	0.4	1.4	3.1	2.3	91.4
isoleucine	1.3	0.0	0.5	0.6	0.5	97.1
valine	1.5	0.6	2.7	3.1	1.7	90.4
3-hydroxyisobutyrate	0.8	1.3	2.2	1.3	1.0	93.5
4-deoxyerythronic acid	1.4	0.8	9.0	2.5	3.0	83.4
4-deoxythreonic acid	1.5	1.5	3.8	1.1	1.4	90.7
threonine/ lactate	1.9	1.0	0.6	1.5	0.9	94.1
2-hydroxyisobutyrate	2.0	1.6	1.0	2.9	1.2	91.3
alanine	1.2	1.5	0.6	2.3	1.4	93.1
lysine	1.1	0.0	0.7	1.7	1.0	95.5
acetate	3.1	0.7	1.4	2.5	1.0	91.2
p-cresol sulfate	1.9	0.2	0.9	1.3	1.5	94.2
succinate	1.8	0.3	2.5	1.1	1.0	93.3
glutamine	2.2	0.3	0.4	1.1	0.2	95.9
citrate	1.9	2.4	2.8	1.6	3.9	87.5
dimethylamine	1.1	1.4	0.5	1.6	0.8	94.7
trimethylamine	1.2	0.2	1.1	0.9	0.9	95.7
carnitine/ choline	1.3	0.5	2.9	2.2	1.0	92.2
trimethylamine *N*-oxide	1.3	1.0	1.0	1.1	0.5	95.1
scyllo-inositol	15.8	0.1	0.8	1.5	1.4	80.4
taurine	3.7	0.4	1.9	3.1	1.4	89.6
glycine	1.7	1.2	0.5	1.6	4.1	90.8
creatine	1.5	1.2	1.1	2.6	3.0	90.6
hippurate	1.6	1.6	3.5	7.8	7.4	78.0
formate	1.0	0.2	3.6	1.0	0.8	93.5
pantothenate	1.0	0.2	6.7	2.8	0.7	88.7
3-hydroxybutyrate/ 3-aminoisobutyrate	1.5	1.6	0.2	3.7	2.1	90.9
3-hydroxyisovalerate	1.1	1.3	2.8	1.8	1.1	91.9
N-acetyl-neuraminic acid	2.6	2.4	0.7	2.3	1.1	90.8
acetone	3.2	1.1	3.3	1.2	4.0	87.2
5-oxoproline	1.3	0.4	1.2	1.4	1.3	94.4
3-aminoisobutyrate	2.1	0.2	0.6	2.7	1.4	93.0
proline betaine	0.5	0.5	0.3	2.7	1.7	94.3
glucose	1.0	1.1	0.7	1.1	0.6	95.6
sucrose	2.2	0.4	2.5	2.3	5.5	87.1
urea	2.1	0.3	1.8	2.9	1.4	91.4
N-methyl-2-pyridone-5-carboxamide	1.9	0.2	1.0	0.9	1.8	94.1
p-hydroxyphenylacetate	1.5	1.8	0.5	1.7	4.2	90.4
tyrosine	2.1	0.6	5.0	0.6	2.0	89.8
3-Indoxylsulfate	1.2	0.3	1.3	0.8	0.2	96.2
N-methylpicolinic acid	1.3	0.1	0.3	0.7	1.6	96.0
N-methylnicotinic acid	0.4	1.2	1.5	4.1	0.1	92.7
N-methyl nicotinamide	1.4	5.5	1.3	1.2	1.1	89.6

Supplementary Table 11 Variance decomposition analysis of serum metabolites (% variance explained)

	analytical	pre-analytical	demographic	dietary	cohort	residual
C0	3.6	0.7	6.9	0.4	0.3	88.0
C10	3.3	1.0	5.8	2.0	2.6	85.3
C10:1	3.2	1.2	7.4	2.5	5.7	80.0
C10:2	6.2	0.0	4.6	2.8	6.5	80.0
C12	3.7	1.2	8.1	1.8	3.0	82.3
C12-DC	35.8	0.0	0.3	0.6	0.1	63.2
C12:1	5.0	1.3	6.4	1.7	2.3	83.2
C14	42.6	0.4	2.6	0.9	1.4	52.0
C14:1	2.2	2.6	7.0	1.4	1.3	85.5
C14:1-OH	6.7	0.6	4.1	1.2	1.7	85.7
C14:2	2.6	2.0	6.2	2.3	3.5	83.3
C14:2-OH	57.3	0.0	0.4	0.5	0.5	41.3
C16	5.2	1.6	7.0	2.2	3.9	80.0
C16-OH	83.0	0.0	0.1	0.2	0.0	16.7
C16:1	3.0	2.8	4.9	1.3	0.8	87.2
C16:1-OH	17.8	0.7	3.1	1.1	1.3	75.9
C16:2	28.3	0.9	2.8	0.8	0.4	66.7
C16:2-OH	22.6	0.2	3.2	0.9	1.1	72.0
C18	6.1	0.4	5.2	2.3	0.6	85.3
C18:1	4.2	4.8	6.4	2.3	3.0	79.3
C18:2	4.3	2.1	9.5	3.3	6.9	73.8
C2	1.1	3.4	5.2	1.3	2.4	86.5
C3	16.7	1.4	4.7	0.8	2.3	74.1
C3-DC (C4-OH)	16.6	0.5	3.8	2.3	1.1	75.7
C3-OH	68.0	0.0	0.1	0.4	0.2	31.4
C4	14.7	0.5	1.3	1.6	1.6	80.4
C4:1	64.0	0.0	0.2	0.6	0.3	34.8
C6 (C4:1-DC)	7.4	1.1	4.6	1.7	2.5	82.8
C5	2.6	1.3	4.4	2.5	1.1	88.1
C5-M-DC	55.4	0.1	0.9	0.7	0.3	42.7
C5-OH (C3-DC-M)	17.4	0.6	1.6	1.7	0.4	78.4
C5:1	10.6	0.1	0.7	3.2	0.7	84.7
C5:1-DC	59.3	0.3	0.2	0.7	0.2	39.3
C5-DC (C6-OH)	34.6	0.3	1.5	1.3	0.4	61.9
C6:1	24.0	0.1	3.1	3.8	1.8	67.2
C7-DC	39.3	0.6	2.4	0.6	0.6	56.6
C8	9.8	1.0	4.6	1.6	2.1	81.0
C9	14.5	0.0	2.6	6.4	7.0	69.5
Ala	3.9	2.0	2.6	2.1	2.3	87.1
Arg	4.8	0.5	0.8	1.9	4.4	87.7
Asn	4.7	0.8	1.3	2.4	4.6	86.2
Asp	5.6	4.3	13.5	4.3	11.3	61.1
Cit	6.5	0.0	3.8	1.6	4.3	83.8
Gln	5.4	0.0	3.2	2.5	7.6	81.2
Glu	2.1	0.5	23.8	2.8	5.4	65.5
Gly	6.2	0.3	2.5	2.4	4.8	83.8
His	9.2	0.9	0.5	1.7	1.0	86.7
Ile	1.9	0.3	3.8	4.0	5.6	84.4
Leu	2.1	0.3	3.5	4.3	4.3	85.4
Lys	6.0	1.1	5.4	1.6	1.9	84.0
Met	11.5	0.8	0.9	1.5	1.6	83.7
Orn	4.9	0.4	4.7	2.5	4.8	82.7
Phe	4.7	1.0	2.9	4.5	4.2	82.6
Pro	2.9	1.5	1.9	0.6	2.1	91.1
Ser	7.0	0.6	5.4	0.9	2.9	83.2
Thr	4.4	0.7	5.3	1.5	3.5	84.7
Trp	9.4	0.9	3.0	2.7	3.2	80.9
Tyr	2.4	0.4	2.5	2.4	3.4	88.8
Val	3.3	0.3	5.1	4.0	4.2	83.2
Ac-Orn	3.6	0.1	2.0	7.4	12.6	74.3
ADMA	7.2	0.3	5.3	1.1	3.2	82.9
alpha-AAA	12.2	0.4	3.6	3.3	3.5	77.1
Creatinine	3.8	0.6	14.1	2.0	1.7	77.7
Kynurenine	4.6	0.6	1.5	1.2	1.4	90.7
Met-SO	6.9	0.3	17.3	2.7	4.2	68.6
Putrescine	14.1	0.5	1.9	1.0	1.1	81.5
SDMA	59.7	0.1	1.3	0.4	0.5	37.9
Serotonin	2.6	1.3	4.9	1.5	5.2	84.5
Spermidine	29.6	2.4	0.9	1.0	1.7	64.4
Spermine	51.2	0.3	0.3	0.3	0.8	47.0
t4-OH-Pro	5.6	0.1	1.9	2.6	0.4	89.4
Taurine	2.5	3.4	8.9	4.4	13.9	66.9
total DMA	32.5	0.1	4.7	0.9	3.9	58.0
lysoPC a C14:0	17.5	0.8	1.6	3.4	2.5	74.2
lysoPC a C16:0	13.2	0.3	1.0	2.9	3.8	78.7
lysoPC a C16:1	8.3	0.5	2.8	2.3	4.4	81.7

lysoPC a C17:0	9.5	1.6	3.1	5.6	5.0	75.1
lysoPC a C18:0	12.6	0.2	0.3	2.0	2.3	82.6
lysoPC a C18:1	8.3	0.3	3.4	5.2	9.1	73.8
lysoPC a C18:2	6.0	0.6	3.1	3.5	6.5	80.2
lysoPC a C20:3	5.2	0.1	3.0	9.6	8.8	73.4
lysoPC a C20:4	8.0	0.0	2.5	6.0	7.6	75.9
lysoPC a C24:0	19.8	0.1	0.9	0.8	1.7	76.8
lysoPC a C26:0	20.5	0.1	0.2	1.1	0.3	77.9
lysoPC a C26:1	25.5	0.0	0.3	0.6	0.4	73.1
lysoPC a C28:0	18.6	0.1	0.2	1.2	0.3	79.6
lysoPC a C28:1	20.4	0.2	0.7	1.1	1.3	76.4
PC aa C24:0	17.7	0.0	0.4	0.6	0.1	81.2
PC aa C28:1	7.8	0.4	3.5	6.9	4.1	77.3
PC aa C30:0	5.4	0.2	1.1	4.8	5.1	83.4
PC aa C30:2	26.5	0.2	0.2	0.6	0.0	72.4
PC aa C32:0	17.9	0.1	1.8	1.1	4.6	74.6
PC aa C32:1	6.3	0.1	2.0	3.4	7.1	81.1
PC aa C32:2	6.5	0.2	2.1	2.0	1.1	88.0
PC aa C32:3	17.6	0.2	2.9	4.9	4.7	69.6
PC aa C34:1	12.9	0.1	1.0	2.6	6.0	77.3
PC aa C34:2	15.4	0.1	2.3	2.8	4.6	74.9
PC aa C34:3	8.4	0.4	2.7	5.1	7.5	75.9
PC aa C34:4	6.1	0.2	6.2	1.7	0.6	85.3
PC aa C36:0	7.7	0.3	5.3	9.1	3.9	73.7
PC aa C36:1	8.3	0.3	1.3	1.8	4.7	83.5
PC aa C36:2	13.3	0.1	0.4	1.5	2.1	82.6
PC aa C36:3	13.0	0.2	1.1	4.6	3.2	77.9
PC aa C36:4	11.3	0.0	2.2	3.0	4.9	78.5
PC aa C36:5	3.3	0.3	3.1	18.8	19.8	54.6
PC aa C36:6	4.5	0.3	8.3	15.4	12.3	59.3
PC aa C38:0	5.9	0.5	7.2	14.7	5.6	66.0
PC aa C38:1	4.7	0.4	3.7	8.8	6.7	75.7
PC aa C38:3	8.0	0.3	8.6	3.5	1.7	77.8
PC aa C38:4	8.1	0.0	5.2	2.5	3.5	80.6
PC aa C38:5	7.4	0.2	1.2	6.7	6.4	78.0
PC aa C38:6	4.3	0.6	6.7	15.5	9.6	63.3
PC aa C40:1	9.2	0.9	2.2	8.9	4.5	74.3
PC aa C40:2	8.7	0.4	1.7	4.1	1.8	83.3
PC aa C40:3	15.2	0.5	1.6	5.4	3.9	73.5
PC aa C40:4	6.1	0.1	3.6	4.0	1.7	84.4
PC aa C40:5	5.2	0.0	1.6	3.9	5.2	84.0
PC aa C40:6	2.9	0.5	9.7	14.9	11.7	60.4
PC aa C42:0	5.2	0.5	3.2	7.0	4.2	80.1
PC aa C42:1	6.5	0.6	2.1	6.4	2.6	81.8
PC aa C42:2	8.4	0.4	2.9	6.2	2.5	79.7
PC aa C42:4	10.5	0.1	0.8	0.8	0.3	87.6
PC aa C42:5	8.0	0.2	3.1	11.8	15.0	62.0
PC aa C42:6	6.2	0.6	3.4	14.9	18.6	56.3
PC ae C30:0	9.8	0.9	3.3	6.1	8.3	71.7
PC ae C30:1	26.4	0.2	0.9	0.6	0.5	71.4
PC ae C30:2	18.0	0.5	2.7	1.3	1.6	75.9
PC ae C32:1	19.4	0.0	3.2	0.8	2.7	73.9
PC ae C32:2	23.2	0.2	5.9	2.3	1.9	66.4
PC ae C34:0	9.6	0.2	3.9	6.1	6.8	73.4
PC ae C34:1	17.4	0.1	3.2	1.4	3.8	74.2
PC ae C34:2	12.4	0.1	2.7	1.9	4.6	78.3
PC ae C34:3	12.9	0.1	0.7	1.6	2.2	82.5
PC ae C36:0	18.4	0.7	3.4	3.2	4.7	69.7
PC ae C36:1	11.5	0.2	1.7	5.8	6.6	74.2
PC ae C36:2	11.7	0.8	2.5	3.8	2.4	78.9
PC ae C36:3	13.0	0.0	3.9	2.7	2.4	77.9
PC ae C36:4	8.4	0.0	7.9	5.9	8.3	69.5
PC ae C36:5	8.8	0.0	4.1	3.1	4.1	79.9
PC ae C38:0	6.4	0.5	5.3	18.4	13.4	55.9
PC ae C38:1	7.7	0.2	1.5	1.8	2.7	86.1
PC ae C38:2	10.5	0.3	2.0	2.1	0.3	84.8
PC ae C38:3	9.6	0.4	0.9	2.6	1.1	85.5
PC ae C38:4	9.9	0.2	5.9	2.2	3.3	78.5
PC ae C38:5	9.6	0.0	6.8	4.7	4.1	74.8
PC ae C38:6	6.3	0.3	7.5	8.9	3.8	73.2
PC ae C40:1	10.8	0.2	3.8	6.1	1.9	77.1
PC ae C40:2	5.2	0.4	1.9	13.4	15.2	63.9
PC ae C40:3	9.1	0.3	0.9	1.5	1.4	86.8
PC ae C40:4	10.4	0.1	2.8	1.2	1.3	84.1
PC ae C40:5	8.2	0.3	3.1	1.3	0.5	86.7
PC ae C40:6	4.9	0.8	7.0	12.9	10.0	64.4
PC ae C42:0	14.6	0.8	1.5	10.5	7.3	65.3
PC ae C42:1	14.7	0.2	1.1	1.8	1.1	81.2
PC ae C42:2	8.2	0.6	1.9	6.5	7.5	75.3
PC ae C42:3	10.3	1.1	2.8	4.6	3.2	77.8
PC ae C42:4	8.5	0.2	3.0	0.7	0.3	87.4

PC ae C42:5	7.1	0.5	3.2	0.7	0.1	88.4
PC ae C44:3	15.1	0.3	1.5	0.7	2.1	80.3
PC ae C44:4	7.5	0.4	2.3	1.1	1.0	87.6
PC ae C44:5	5.8	0.2	3.6	0.5	0.2	89.7
PC ae C44:6	6.0	0.2	1.6	2.0	3.0	87.1
SM (OH) C14:1	10.6	0.8	4.2	7.1	7.6	69.7
SM (OH) C16:1	13.6	0.8	6.0	6.8	8.1	64.6
SM (OH) C22:1	38.0	0.1	1.9	1.6	4.6	53.9
SM (OH) C22:2	39.2	0.2	1.0	2.7	3.9	52.9
SM (OH) C24:1	33.1	0.2	0.7	1.2	2.9	61.8
SM C16:0	22.0	0.2	1.3	0.7	1.1	74.7
SM C16:1	18.9	0.0	8.7	1.0	1.5	69.9
SM C18:0	22.3	0.1	3.5	0.7	1.7	71.8
SM C18:1	20.2	0.1	9.5	0.9	1.3	68.0
SM C20:2	21.6	0.0	1.7	0.8	2.0	73.9
SM C24:0	44.6	0.5	1.4	0.9	0.6	52.1
SM C24:1	42.5	0.2	0.5	2.1	2.5	52.2
SM C26:0	22.1	0.6	1.6	2.0	2.8	70.9
SM C26:1	20.5	0.6	1.1	5.2	5.4	67.2
H1	4.9	0.5	4.3	0.6	2.9	86.9

Supplementary Table 12 Modeled results with Box-Cox transformation and QRILC imputation - metabolites associated with age

metabolite	effect size (SD/ year)	lower CI	upper CI	p-value	I^2
creatinine (urine)	0.39	0.26	0.53	4.7E-09	0%
Creatinine (serum)	0.30	0.17	0.43	4.0E-06	19%

Supplementary Table 13 Modeled results with Box-Cox transformation and QRILC imputation - metabolites associated with sex

metabolite	effect size	lower CI	upper CI	p-value	I^2
isoleucine (urine)	-0.28	-0.41	-0.16	6.2E-06	67%
glutamine (urine)	0.21	0.09	0.34	6.9E-04	0%
citrate (urine)	-0.21	-0.33	-0.09	4.8E-04	21%
5-oxoproline (urine)	0.23	0.10	0.36	3.7E-04	0%
tyrosine (uirne)	0.44	0.32	0.56	3.8E-13	0%
C10	-0.23	-0.35	-0.11	2.6E-04	26%
C12	-0.29	-0.42	-0.17	1.7E-06	18%
C14:1	-0.34	-0.46	-0.21	7.0E-08	12%
C14:1-OH	-0.23	-0.35	-0.11	2.1E-04	15%
C14:2	-0.24	-0.36	-0.12	9.2E-05	13%
C16:1	-0.26	-0.38	-0.14	2.4E-05	0%
Lys	-0.24	-0.36	-0.12	9.4E-05	53%
Orn	-0.35	-0.47	-0.23	2.1E-08	20%
Ser	-0.26	-0.39	-0.14	2.2E-05	8%
Putrescine	-0.27	-0.38	-0.15	6.4E-06	39%
Serotonin	0.32	0.20	0.44	1.6E-07	54%
SM C16:1	-0.35	-0.46	-0.24	3.2E-10	0%
SM C18:0	-0.22	-0.33	-0.11	9.6E-05	0%
SM C18:1	-0.36	-0.46	-0.25	1.4E-10	0%
H1	0.23	0.11	0.35	2.4E-04	7%

Metabolites found higher in male children are shown as positive and metabolites found higher in female children are shown as negative

Supplementary Table 14 Modeled results with Box-Cox transformation and QRILC imputation - metabolites associated with BMI z-score

metabolite	effect size (SD/ unit z-score)	lower CI	upper CI	p-value	I^2
valine (urine)	0.10	0.05	0.15	2.0E-04	0%
4-deoxyerythronic acid (urine)	0.21	0.16	0.26	5.7E-16	30%
p-cresol sulfate (urine)	-0.11	-0.16	-0.06	3.9E-05	57%
pantothenate (urine)	-0.12	-0.17	-0.07	2.2E-06	11%
C0	0.19	0.14	0.24	3.2E-13	0%
C18	-0.13	-0.18	-0.08	8.1E-07	21%
C3	0.15	0.10	0.20	7.4E-10	0%
C5	0.13	0.08	0.19	4.0E-07	0%
Asp	0.10	0.05	0.15	1.2E-04	13%
Glu	0.22	0.17	0.27	3.7E-17	0%
Ile	0.11	0.05	0.16	7.5E-05	0%
Leu	0.12	0.07	0.17	4.9E-06	0%
Lys	0.10	0.05	0.15	1.3E-04	0%
Phe	0.13	0.07	0.18	1.4E-06	12%
Pro	0.10	0.05	0.15	1.3E-04	0%
Tyr	0.10	0.05	0.15	2.7E-04	0%
Val	0.13	0.08	0.19	4.1E-07	0%
ADMA	0.10	0.04	0.15	2.5E-04	0%
alpha-AAA	0.10	0.05	0.15	8.7E-05	0%
Kynurenine	0.10	0.05	0.15	1.1E-04	37%
lysoPC a C16:1	0.14	0.09	0.19	9.5E-08	0%
lysoPC a C18:1	-0.09	-0.14	-0.04	1.9E-04	0%
lysoPC a C18:2	-0.12	-0.17	-0.07	4.9E-06	0%
PC aa C32:1	0.11	0.06	0.16	1.7E-05	0%
PC aa C32:3	0.10	0.05	0.15	5.2E-05	0%
PC aa C34:4	0.13	0.08	0.19	2.4E-07	0%
PC aa C36:5	0.10	0.05	0.15	1.4E-04	0%
PC aa C36:6	0.10	0.04	0.15	2.6E-04	0%
PC aa C38:1	-0.10	-0.15	-0.05	1.4E-04	32%
PC aa C38:3	0.20	0.15	0.25	5.7E-16	0%
PC aa C38:4	0.13	0.08	0.19	1.5E-07	0%
PC aa C40:6	0.10	0.05	0.15	1.2E-04	0%
PC ae C34:0	-0.09	-0.14	-0.04	1.9E-04	0%
PC ae C34:1	-0.09	-0.14	-0.04	2.6E-04	0%
PC ae C36:0	-0.10	-0.15	-0.05	2.3E-05	0%
PC ae C36:2	-0.12	-0.17	-0.07	2.0E-06	0%
PC ae C38:2	-0.10	-0.15	-0.05	5.2E-05	0%
PC ae C40:4	-0.11	-0.16	-0.06	2.1E-05	21%
PC ae C40:5	-0.10	-0.15	-0.05	1.0E-04	12%
PC ae C40:6	-0.10	-0.15	-0.05	1.5E-04	0%
PC ae C42:3	-0.11	-0.16	-0.06	1.6E-05	0%
PC ae C42:4	-0.14	-0.19	-0.09	1.6E-07	0%
PC ae C44:4	-0.11	-0.16	-0.05	6.7E-05	23%
SM C16:0	-0.10	-0.14	-0.05	7.3E-05	17%
SM C16:1	0.16	0.12	0.21	1.3E-11	0%
SM C18:1	0.15	0.11	0.20	8.9E-11	0%

Supplementary Table 15 Modeled results with Box-Cox transformation and QRILC imputation - metabolites associated with dietary intake

metabolite-diet pair	effect size (SD/ times per week)	lower CI	upper CI	p-value	I^2
creatine (urine) with meat	0.027	0.013	0.040	1.3E-04	0%
pantothenate (urine) with dairy	0.009	0.004	0.015	7.5E-04	37%
hippurate (urine) with vegetables	0.021	0.011	0.031	2.6E-05	0%
leucine (urine) with fruits	-0.014	-0.022	-0.006	6.0E-04	0%
threonine/ lactate (urine) with fruits	-0.013	-0.021	-0.005	9.6E-04	19%
alanine (urine) with fruits	-0.014	-0.022	-0.006	7.7E-04	29%
succinate (urine) with fruits	0.015	0.007	0.023	1.7E-04	30%
glutamine (urine) with fruits	-0.020	-0.028	-0.013	3.4E-07	0%
scyllo-inositol (urine) with fruits	0.018	0.011	0.025	3.0E-07	58%
hippurate (urine) with fruits	0.026	0.018	0.034	4.4E-11	50%
3-aminoisobutyrate (urine) with fruits	0.015	0.007	0.023	1.9E-04	24%
proline betaine (urine) with fruits	0.023	0.015	0.031	1.5E-08	19%
N-methylnicotinic acid (urine) with fruits	0.018	0.010	0.026	1.6E-05	60%
3-hydroxybutyrate/ 3-aminoisobutyrate (urine) with beverages	-0.034	-0.053	-0.016	3.2E-04	0%
PC ae C36:3 with meat	0.024	0.011	0.037	2.3E-04	0%
PC ae C36:4 with meat	0.031	0.018	0.044	3.5E-06	0%
PC ae C36:5 with meat	0.026	0.013	0.040	1.1E-04	0%
PC ae C38:5 with meat	0.027	0.014	0.040	5.7E-05	20%
PC aa C36:0 with fish	0.091	0.064	0.119	9.0E-11	56%
PC aa C36:5 with fish	0.063	0.035	0.091	1.3E-05	43%
PC aa C36:6 with fish	0.087	0.059	0.114	7.9E-10	0%
PC aa C38:0 with fish	0.108	0.081	0.135	9.1E-15	74%
PC aa C38:6 with fish	0.113	0.085	0.140	1.2E-15	57%
PC aa C40:1 with fish	0.055	0.027	0.082	1.1E-04	0%
PC aa C40:4 with fish	-0.057	-0.084	-0.030	3.5E-05	19%
PC aa C40:6 with fish	0.100	0.072	0.128	1.5E-12	48%
PC aa C42:0 with fish	0.052	0.024	0.080	2.4E-04	0%
PC aa C42:2 with fish	0.057	0.029	0.084	5.3E-05	3%
PC ae C32:2 with fish	0.047	0.022	0.072	2.3E-04	36%
PC ae C38:0 with fish	0.086	0.058	0.113	8.7E-10	37%
PC ae C38:6 with fish	0.074	0.046	0.102	1.9E-07	56%
PC ae C40:6 with fish	0.087	0.059	0.115	9.0E-10	31%
Gly with dairy	-0.010	-0.016	-0.005	2.8E-04	31%
PC aa C28:1 with dairy	0.015	0.010	0.020	3.5E-08	59%
PC aa C30:0 with dairy	0.012	0.007	0.017	1.6E-05	69%
PC aa C38:0 with dairy	-0.013	-0.018	-0.008	2.1E-06	0%
PC ae C30:0 with dairy	0.015	0.009	0.020	3.0E-08	73%
PC ae C38:6 with dairy	-0.010	-0.016	-0.005	2.2E-04	0%
SM (OH) C14:1 with dairy	0.013	0.007	0.018	3.4E-06	71%
PC ae C30:0 with potatoes	-0.042	-0.064	-0.020	1.7E-04	0%
PC ae C34:0 with potatoes	-0.048	-0.070	-0.026	1.7E-05	29%
SM (OH) C14:1 with potatoes	-0.046	-0.067	-0.024	4.0E-05	50%
Ac-Orn with fruits	0.016	0.008	0.023	7.5E-05	0%
PC aa C38:0 with sweets	-0.022	-0.032	-0.012	1.3E-05	0%
PC aa C38:6 with sweets	-0.022	-0.032	-0.011	3.1E-05	0%
PC ae C38:5 with sweets	-0.021	-0.031	-0.011	2.3E-05	23%
PC ae C38:6 with sweets	-0.021	-0.031	-0.011	5.2E-05	0%
PC ae C40:6 with sweets	-0.023	-0.033	-0.013	8.4E-06	0%
lysoPC a C17:0 with bakery products	-0.026	-0.039	-0.013	6.4E-05	49%
PC aa C36:6 with bakery products	-0.027	-0.040	-0.014	4.2E-05	0%
PC aa C38:0 with bakery products	-0.026	-0.039	-0.014	5.0E-05	51%
PC aa C38:6 with bakery products	-0.027	-0.040	-0.014	4.3E-05	54%
PC aa C40:6 with bakery products	-0.027	-0.040	-0.014	4.3E-05	41%
PC ae C38:0 with bakery products	-0.030	-0.042	-0.017	4.5E-06	18%
PC ae C38:6 with bakery products	-0.025	-0.037	-0.012	1.9E-04	11%
PC ae C40:2 with bakery products	-0.027	-0.039	-0.014	4.5E-05	53%
PC ae C40:6 with bakery products	-0.025	-0.038	-0.013	9.5E-05	35%
SM (OH) C14:1 with bakery products	-0.027	-0.039	-0.014	3.6E-05	14%
SM (OH) C16:1 with bakery products	-0.030	-0.042	-0.018	1.6E-06	32%
C5:1 with beverages	-0.033	-0.050	-0.015	2.1E-04	1%
C6:1 with beverages	-0.031	-0.047	-0.015	1.6E-04	20%
