# Supplementary figures and images for: Determinants of the urinary and serum metabolome in children from six European populations
Source: BMC Med. 2018 Nov 8;16:202. doi: 10.1186/s12916-018-1190-8 (PMC6223046; doi:10.1186/s12916-018-1190-8)

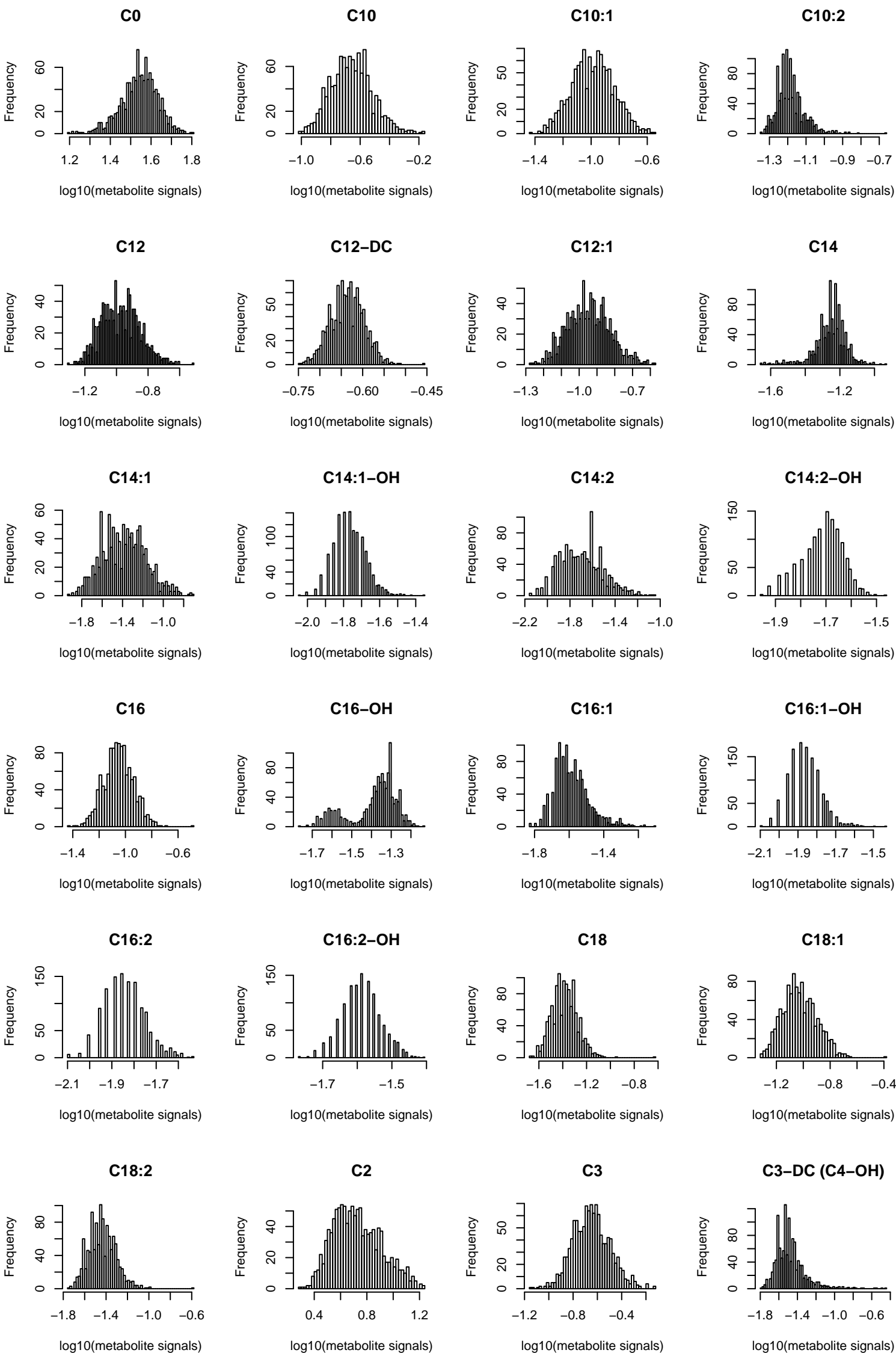

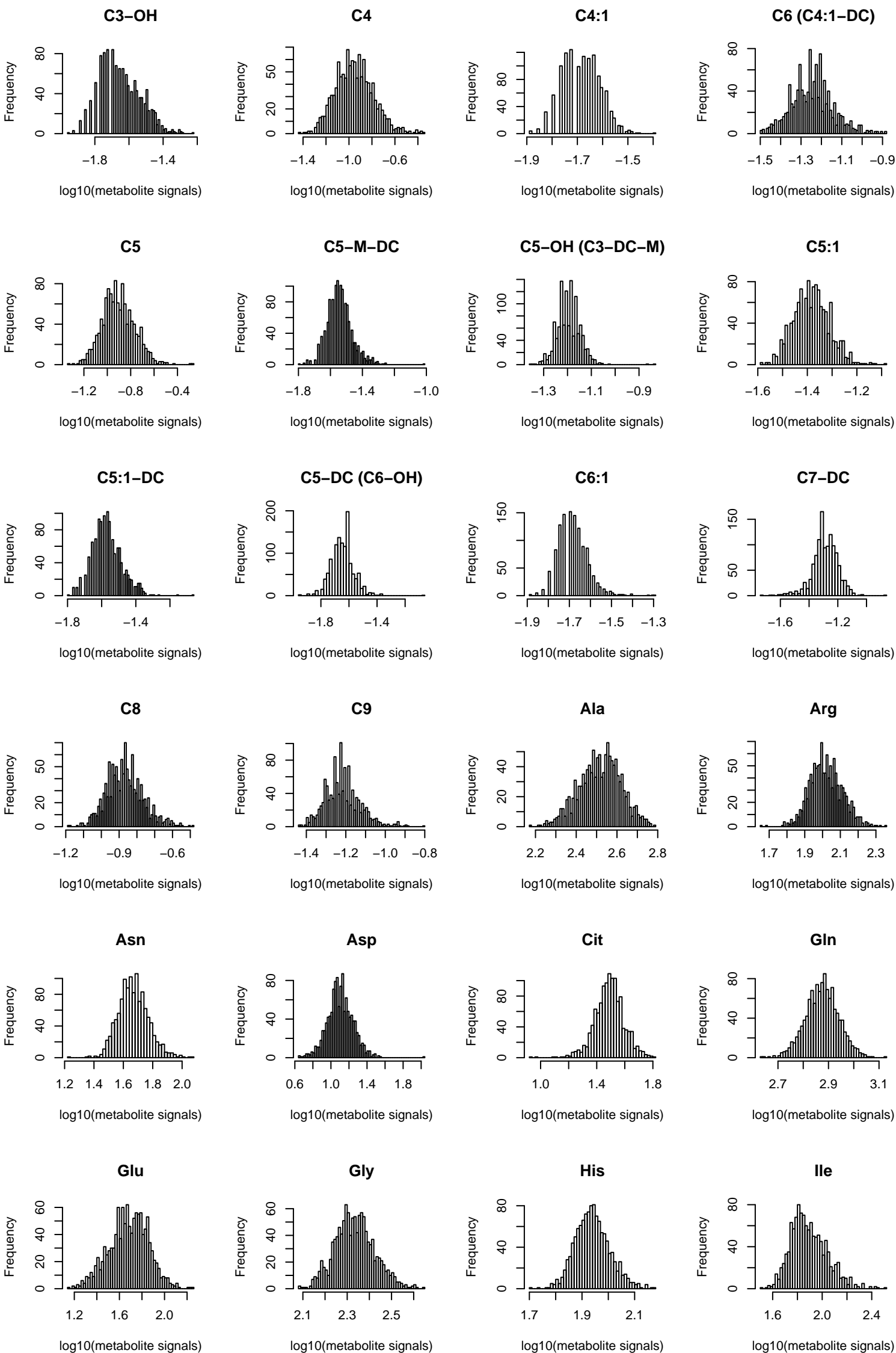

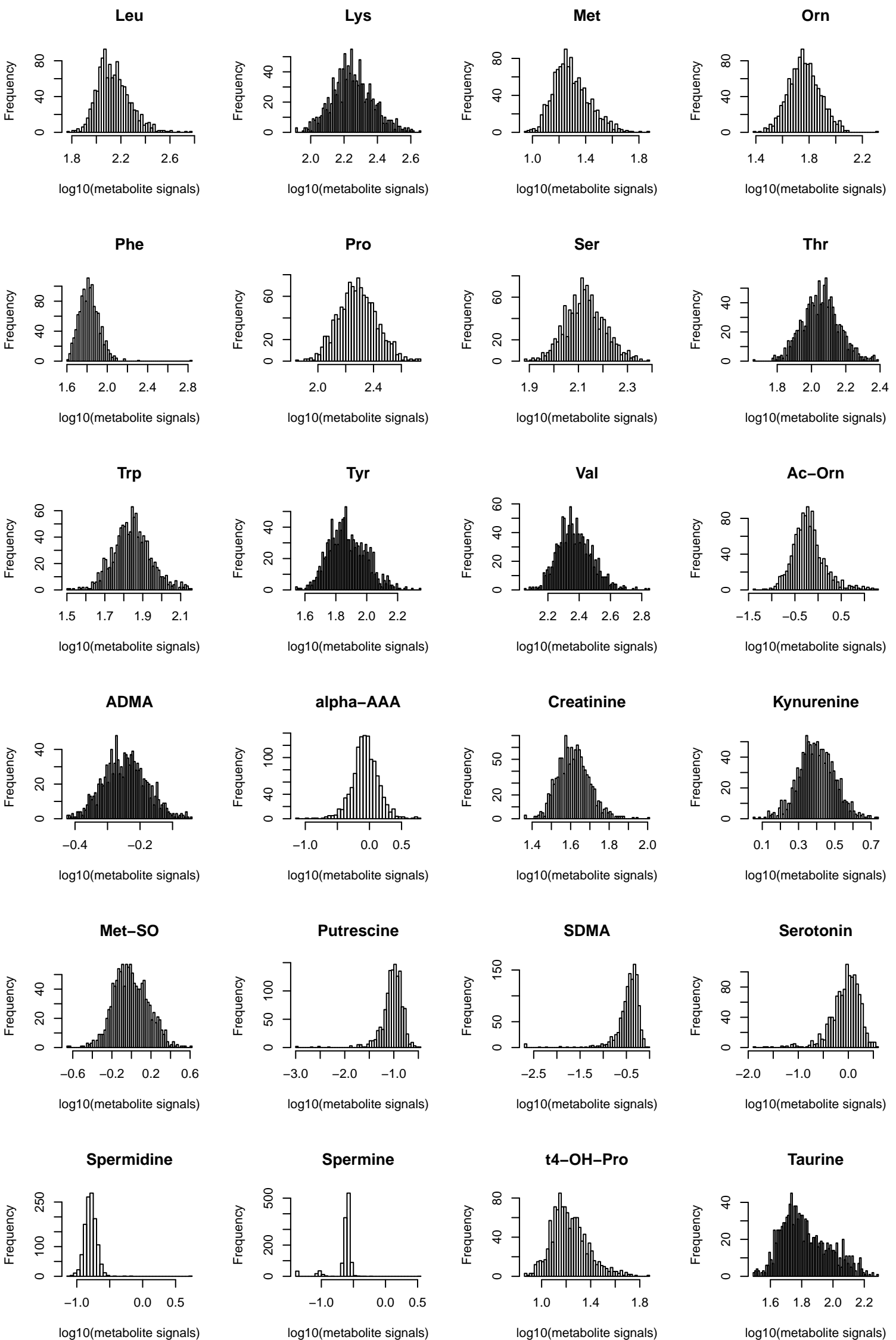

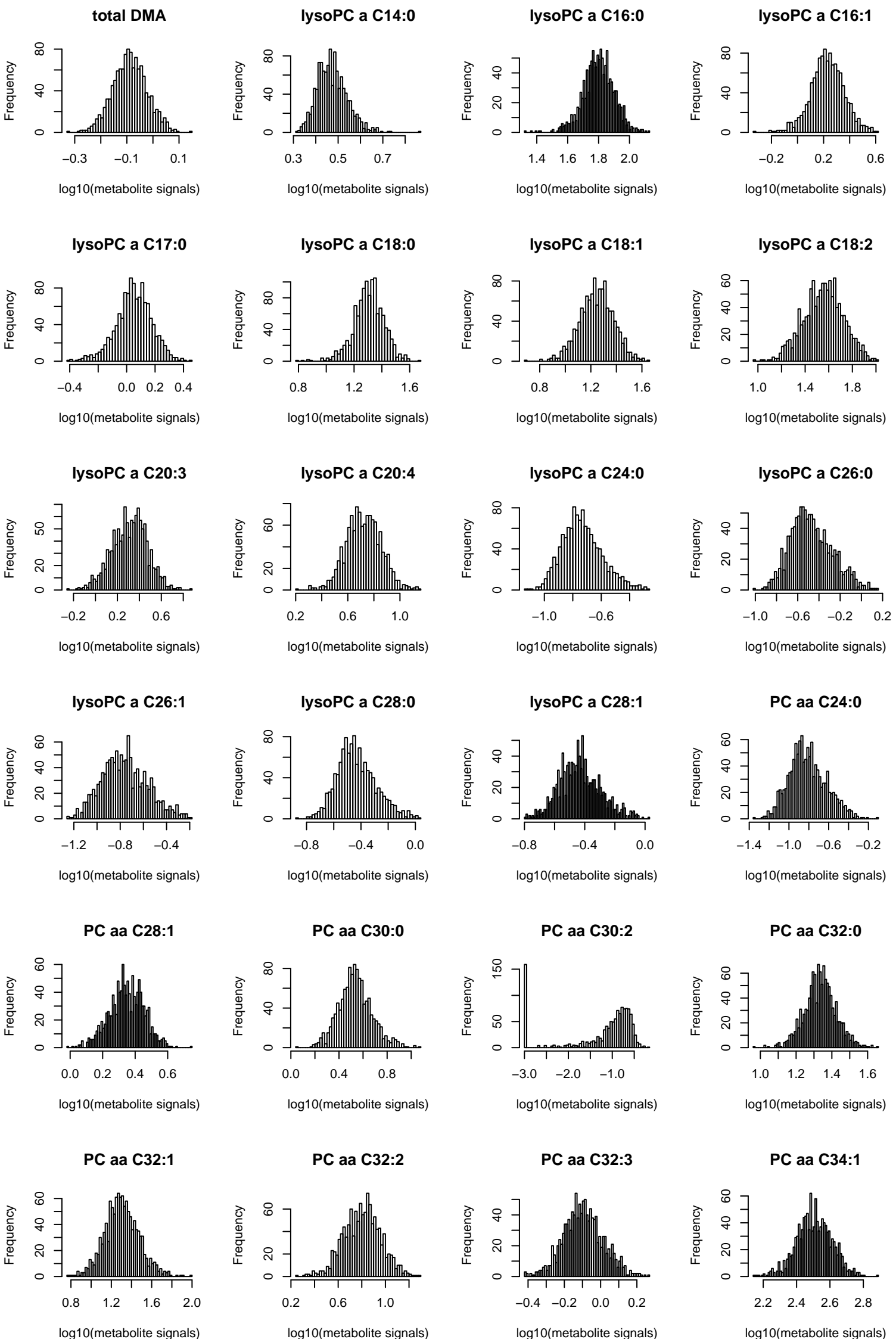

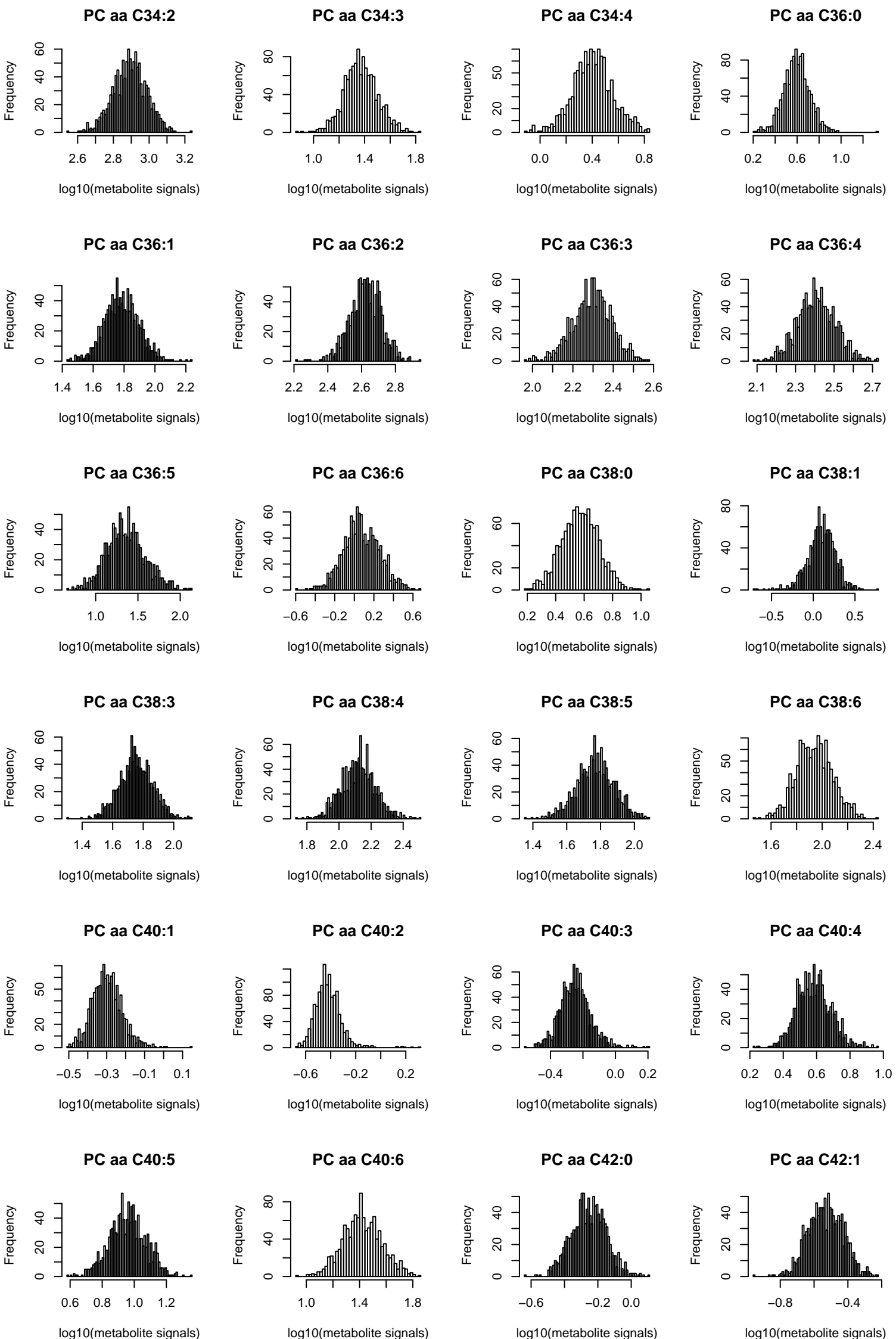

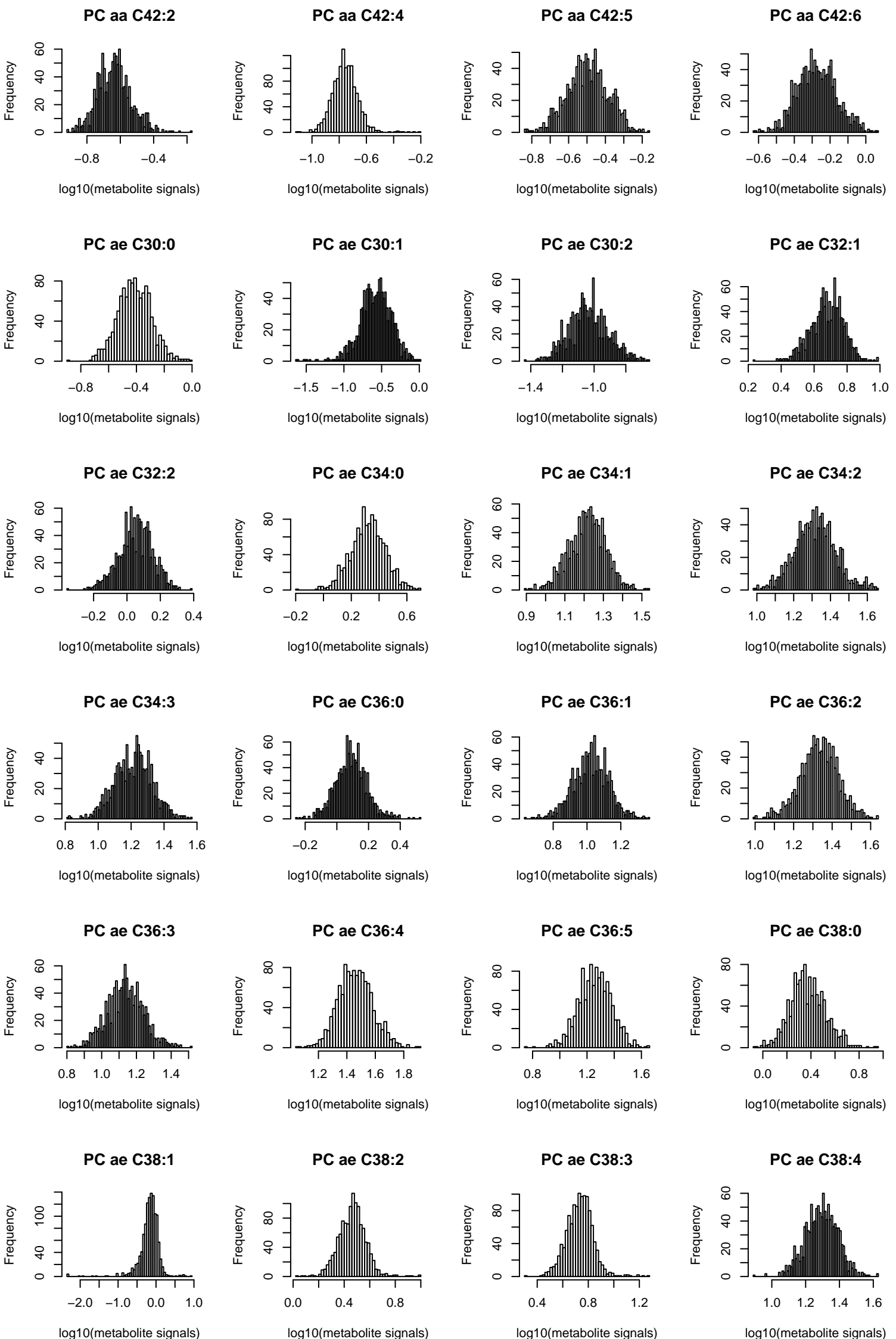

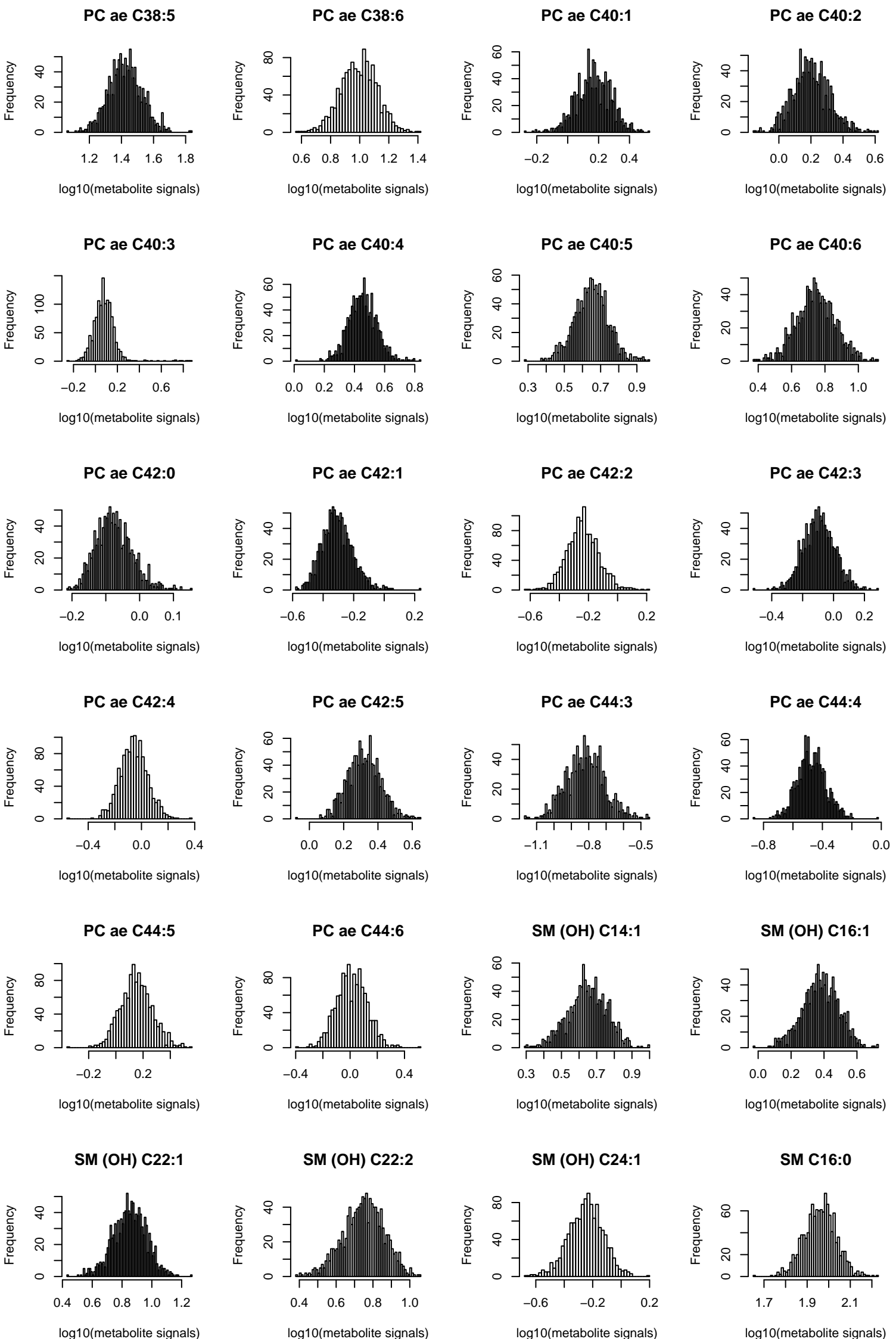

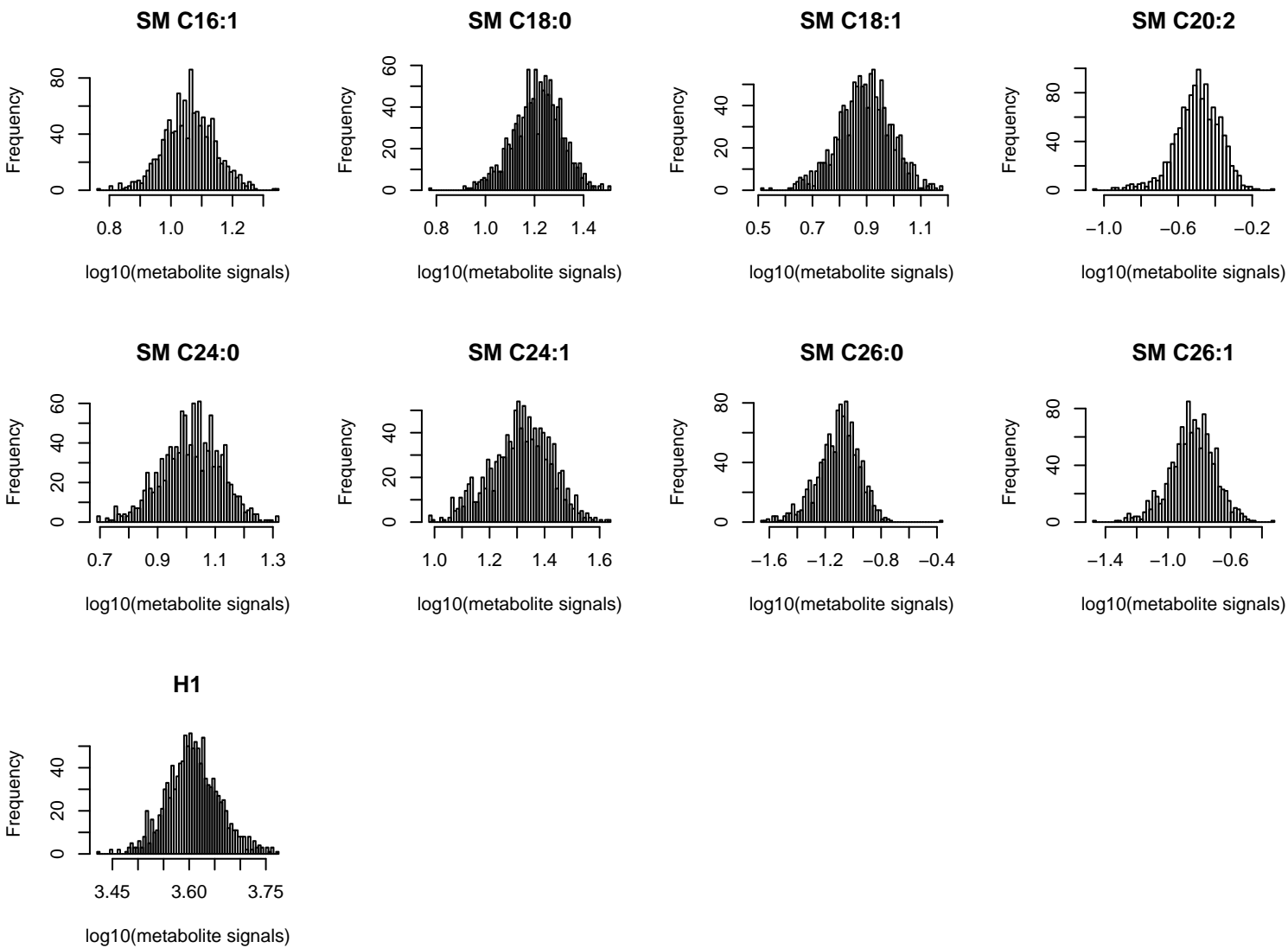

Supplement: Supplementary file 2 — Histograms of serum metabolite distributions. (PDF 111 kb) [file 12916_2018_1190_MOESM2_ESM.pdf]

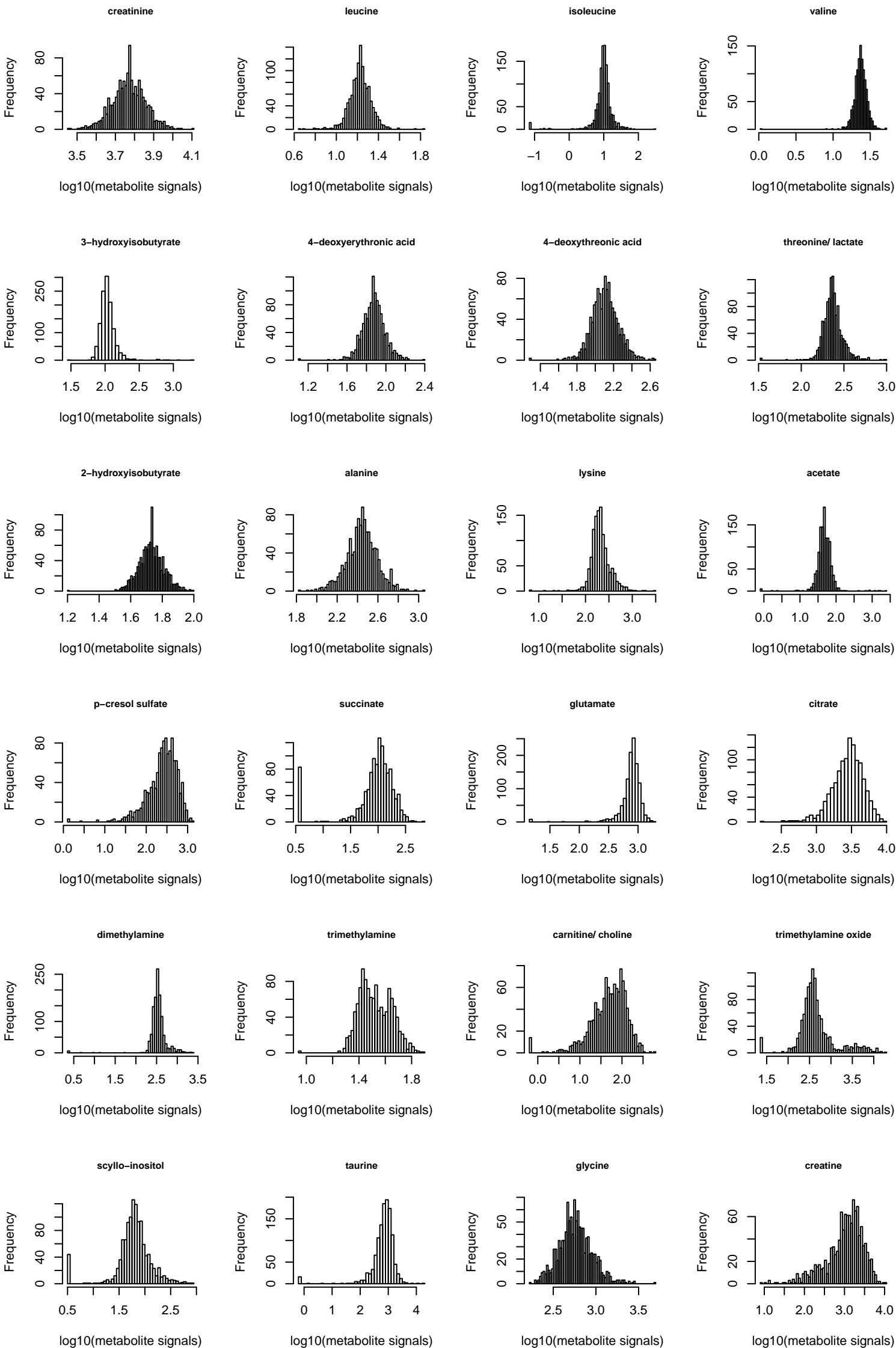

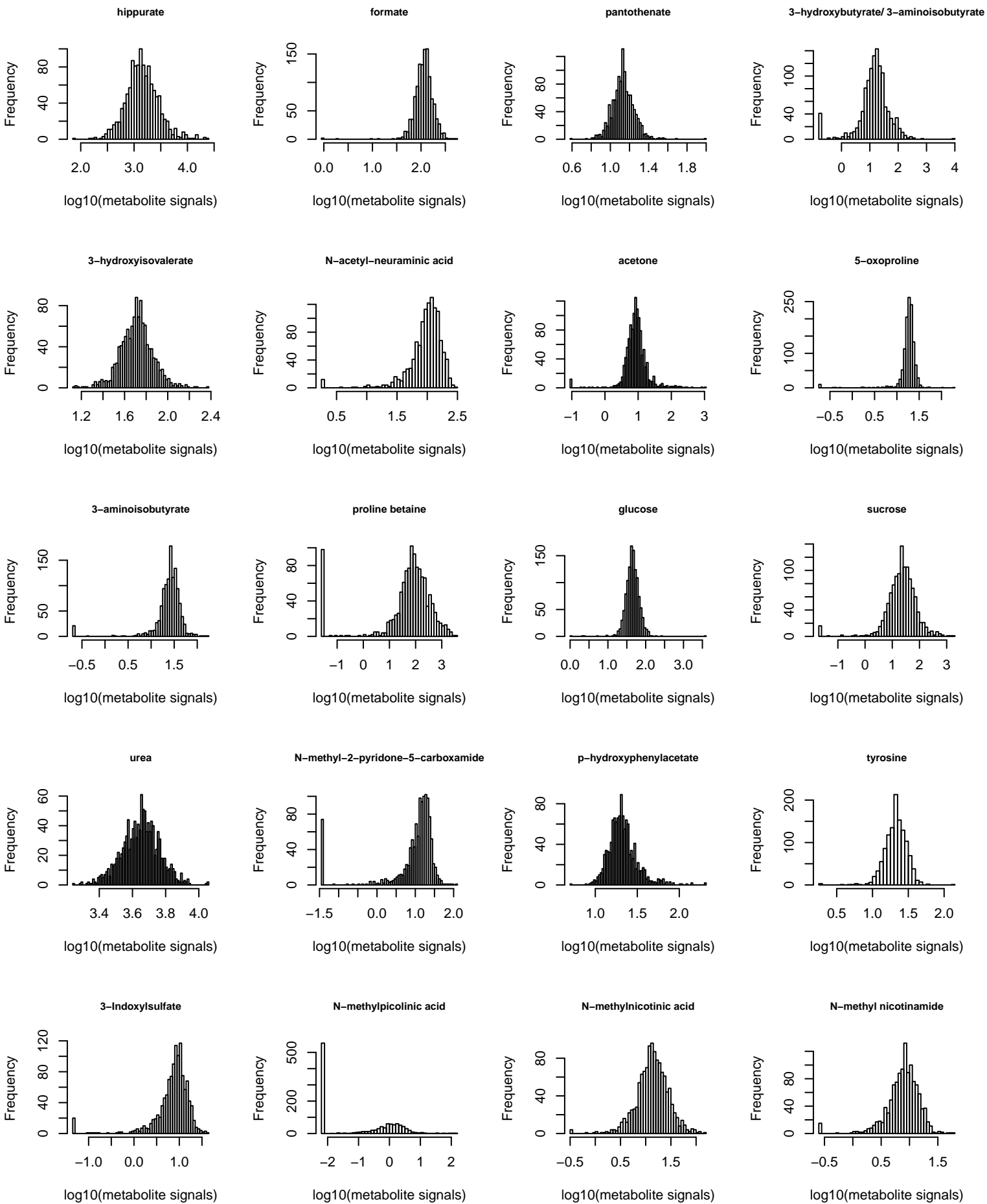

Supplement: Supplementary file 3 — Histograms of urinary metabolite distributions. (PDF 29 kb) [file 12916_2018_1190_MOESM3_ESM.pdf]
